# drLumi: An open-source package to manage data, calibrate, and conduct quality control of multiplex bead-based immunoassays data analysis

**DOI:** 10.1371/journal.pone.0187901

**Published:** 2017-11-14

**Authors:** Hector Sanz, John J. Aponte, Jaroslaw Harezlak, Yan Dong, Aintzane Ayestaran, Augusto Nhabomba, Maxmillian Mpina, Obiang Régis Maurin, Núria Díez-Padrisa, Ruth Aguilar, Gemma Moncunill, Agnandij Selidji Todagbe, Claudia Daubenberger, Carlota Dobaño, Clarissa Valim

**Affiliations:** 1 ISGlobal, Barcelona Ctr. Int. Health Res. (CRESIB), Hospital Clínic - Universitat de Barcelona, Barcelona, Spain; 2 Department of Biostatistics, Indiana University Fairbanks School of Public Health, Indianapolis, Indiana, United States of America; 3 Centro de Investigação em Saúde de Manhiça (CISM), Maputo, Mozambique; 4 Ifakara Health Institute, Bagamoyo, Tanzania; 5 Centre de Recherches Médicales de Lambaréné (CERMEL), Albert Schweitzer Hospital, Lambaréné, Gabon; 6 Institut für Tropenmedizin, University of Tübingen, Tübingen, Germany; 7 Swiss Tropical and Public Health Institute, Basel, Switzerland; 8 University of Basel, Basel, Switzerland; 9 Department of Immunology and Infectious Diseases, Harvard School of Public Health, Boston, Massachusetts, United States of America; 10 Department of Osteopathic Medical Specialties, Michigan State University, College of Osteopathic Medicine East Lansing, Michigan, United States of America; London School of Hygiene and Tropical Medicine, UNITED KINGDOM

## Abstract

Multiplex bead-based immunoassays are used to measure concentrations of several analytes simultaneously. These assays include control standard curves (SC) to reduce between-plate variability and normalize quantitation of analytes of biological samples. Suboptimal calibration might result in large random error and decreased number of samples with analyte concentrations within the limits of quantification. Suboptimal calibration may be a consequence of poor fitness of the functions used for the SC, the treatment of the background noise and the method used to estimate the limits of quantification. Currently assessment of fitness of curves is largely dependent on operator and that may add additional error. Moreover, there is no software to automate data managing and quality control. In this article we present a R package, drLumi, with functions for managing data, calibrating assays and performing quality control. To optimize the assay the package implements: i) three dose-response functions, ii) four approaches for treating background noise and iii) three methods for estimating limits of quantifications. Other implemented functions are focused on the quality control of the fitted standard curve: detection of outliers, estimation of the confidence or prediction interval, and estimation of summary statistics. With demonstration purpose, we apply the software to 30 cytokines, chemokines and growth factors measured in a multiplex bead-based immunoassay in a study aiming to measure correlates of risk or protection from malaria of the RTS,S malaria vaccine nested in the Phase 3 randomized controlled trial of this vaccine.

## Introduction

Multiplex bead-based immunoassays are used to measure concentrations of several cytokines and chemokines, antibodies, or other proteins simultaneously saving sample volume, money, and time. One of the common technologies used for several assays is Luminex systems [1]. These assays have been widely applied in biomarker studies, or more generally in studies searching correlates with diagnosis, immunity, and vaccine-induced protection [2–5]. Generally, in these assays, sets of magnetic beads are pre-gated on a known fluorescence signal, thus allowing multiplex analyte detection. When the assay kits are prepared, each set of beads with a corresponding fluorescence signal is coated with antibodies targeting to capture a specific analyte. The different sets of beads, targeting each analyte of interest, are mixed with the subject’s liquid samples in plates and are allowed to capture all existing analytes in the samples. A second antibody is then added to the plate that binds to the analyte captured by the antibodies adhered to the beads, making a sandwich with the analyte to be quantitated. The assay final raw output is the median fluorescence intensity (MFI) over all beads containing the corresponding analyte that are captured by the reader [1,2,6].

Commonly, to calibrate inherent assay variability and quantitate analyte concentrations, each plate includes wells containing increasing serial dilutions of a reference sample with known concentration of each analyte, also called standard samples. MFIs from these wells are used to fit a sigmoidal (often a 5 parameter log-logistic) curve, called standard curve, with the MFI as a function of the concentration of the analyte in the standard samples. To calibrate each subject’s response, the MFI of the subject sample is converted to analyte concentration using the standard curve. The minimum and maximum concentrations of an analyte that can be reliably quantitated are called lower and higher limits of quantification (LLOQ and HLOQ), respectively. Additionally, to estimate the background noise, negative controls containing no analyte, also called blank controls, are included in each assay plate. The MFI of these negative or blank controls is usually subtracted from the MFI of the standard samples before fitting the standard curve in an attempt to control inherent noise.

When pre-processing the data from the assay, several analytical factors may result in sub-optimal calibration and ultimately lead to failure to quantitate a large amount of samples because concentrations are outside the limits of quantification (LOQ), including a poorly fitted standard curve and the method used to estimate the LOQs. Additionally, the approach used to account for the background noise when estimating standard curves can also affect the fitting of the standard curves and impact the assay calibration and number of samples quantitated. Ultimately, when analyzing assay data, these problems may result in: substantial amount of missing data in some analytes; excessive “noise” in the data decreasing analytical precision and “study power”; and differential error of measurement and biases.

Several packages are available in the R statistical environment [7] to fit nonlinear models and analyze dose-response data, including drc [8] and nCal [9]. These packages allow fitting models using a variety of parametric curves to calibrate samples. drLumi package was customized to multiplex bead immunoassays based on Luminex^®^ technology, although it could be easily expanded to other assays. drLumi adds to the functionality of the aforementioned packages via: i) prompt reading and automating management of data from multiplex immunoassay; ii) producing metrics to quality assurance/quality control (QA/QC) of the assay; iii) providing four alternative approaches to manage background noise; and iv) containing three approaches to estimate LOQs using standard curves estimated through the four- and five-parameter log-logistic and exponential models. The functionality added by the package helps to increase accuracy and precision of the assay in several ways including: i) decreases operator errors when manually managing data or empirically curating curves and identifying outliers; ii) allows real-time monitoring and correction of problems with operators or instruments; iii) provides approaches to fit more precise standard curves allowing more precise quantification of analytes in each plate; iv) allows more precise quantification of analytes by defining a boundary MFI to be used in quantification that optimizes precision.

All functions in the package are written in the R programming language and several of them rely on other R packages easily downloadable, e.g. reshape and Hmisc. The R environment is open-source and available online under the GNU General Public License (GPLv2). This article provides an overview on the usage of the main functions of the package and examples of the implemented methods with a real-life dataset.

## Methods

drLumi is based on the following core functions: *data_selection*, *scluminex*, *loq* and generic *plot* and *summary* functions. Through these functions, the package can automatically read and format the assay data to be used in the pre-processing or lower-level analyses. However, the data used to estimate standard curves do not need to be produced by the package and the user may prefer to provide to the package a dataset managed elsewhere. Additional core functions of drLumi are dedicated to pre-processing the data, i.e., to the estimation of standard curves and calibration of “real-life” test samples with the corresponding curves, and to the automation of QA/QC of the estimated standard curve and the assay in general.

Also, it is possible to flag samples once they are identified as errors due to wells contamination or pipetting errors, for example, and the package will not use those samples for fitting the standard curve. This allows tracking all fitting process. The methods used by each of those functions are further discussed below.

### Automatic data extraction and management

Generally, the instrument that reads the assay is connected to software that produces a report including the MFI of standard samples, negative controls and test samples. drLumi has implemented two specific functions for managing data when the software that produces this report (a raw data) is the xPONENT^®^ software (S1 File). The package reads this report as a dataset and using the *data_selection* function extracts and formats the variables and samples from this report into analyzable datasets. The xPONENT software 3.1 version produces two types of datasets from every assay: i) in one the unit is a bead analyte-specific and associated with this bead is its fluorescence intensity (one dataset for each analyte); ii) in a second dataset the unit is a sample with the MFI over all beads detected. The *lum_import* function allows importing both types of CSV raw datasets. If MFI sample data are imported, a list with all identified datasets and variables is returned and if bead data are imported, all bead files are combined in one dataset. When applying the *lum_export* function to a *lum_import* class object, the function returns all datasets in a proper format to work with and allows exporting the data into several CSV files (for fluorescence-type data) or a compressed file (for bead-type data).

### Models for the calibration curve

Given the underlying biological assumption of these assays, the MFI as a function of concentration is expected to be non-linear, more specifically, sigmoidal with a saturation point after a concentration threshold. The 5-parameter log-logistic (5-parameter LL) functions have often been used to represent this sigmoidal function because they allow the upper and lower asymptotes to have different lengths. However, depending on the specific assay, the 5-parameter LL does not fit well the data or even does not converge. For instance, depending on the number of wells/points used to estimate the curve or when the data fall entirely in one asymptotic region [10], the 4-parameter LL fit may be more appropriate. Also, when several analytes are multiplexed, some standard curves used to calibrate the assay may never reach the upper asymptote while others do reach it, given the maximum concentration of controls for the different analytes. This may happen because the assay and corresponding dilutions are planned to be optimal for the majority of but not necessarily for all analytes. In these cases, an exponential fit may be more appropriate for the standard curve.

Since data from immunoassays are often heteroscedastic, the three non-linear models are fit using logarithm base 10 transformed data to stabilize the variance [11]. Using base 10 logarithms, the parameterization of the five-parameter logistic function implemented in the package [10] is

$$f\left(x;b,c,d,e,f\right)=c+\frac{d-c}{{\left(1+10^{b(x-e)}\right)}^{f}}$$

where *c* is the lower asymptote, *d* is the upper asymptote, *e* is the concentration that produces a response halfway between *c* and *d*, *b* is the slope around the *e* parameter and *f* is the asymmetry for the slope. The asymmetry parameter allows the length of lower and upper asymptotes to be different.

The four-parameter logistic function, has the same expression as the five-parameter when the asymmetry parameter is fixed to be one:

$$f\left(x;b,c,d,e\right)=c+\frac{d-c}{1+10^{b\left(x-e\right)}}$$

The exponential growth function is parameterized as:

$$f\left(x;y_{0},b\right)=y_{0}10^{\frac{x}{b}}$$

where *b* is the growth rate and *y0* is the response in the absence of the analyte.

### Treatment of blank controls

Blank controls are defined in this text as samples estimating the background noise level. Often in multiplex immunoassays, these samples contain only buffer (solution used in all plates wells) and no analyte. In average, across several runs of an unbiased assay (no systematic background signal), the MFI of blank controls shall be close to 0. However, real life assays have random error and thus, the MFI of blank control wells varies across plates.

**Fig 1:**
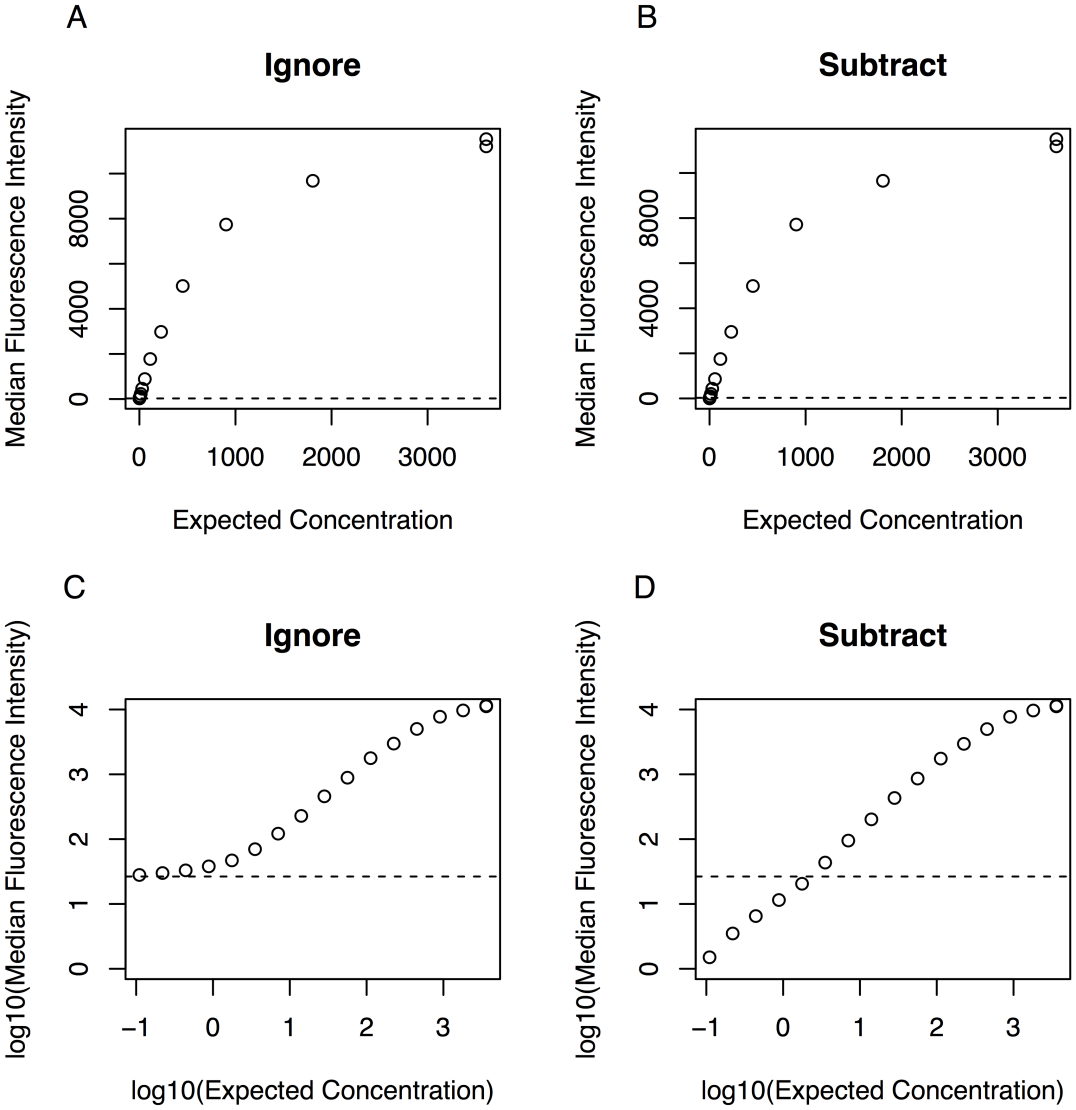
Cartoon showing the influence of the Subtract method into the standard samples. Background noise calculated as the geometric mean of the blank controls is shown as a horizontal dashed line. (A) Standard curve plotting the MFI in the **original scale** as a function of concentration; (B) Standard curve plotting the MFI in the **original scale** as a function of concentration **after subtracting** the MFI of the blank control from all standard samples; (C) Standard curve plotting the MFI in the **log10 scale** as a function of concentration; and (D) Standard curve plotting the MFI in the **log10 scale** as a function of concentration **after subtracting** the MFI of the blank control from all standard samples.

The *scluminex* function implements four methods to account for the background noise estimated by the blank controls when fitting standard curves. These estimates can be based on the geometric mean (GM) of the blank control, if more than one blank control well is included in the assay, or be based on a single blank control well. Traditionally, in immunoassays, the MFI of blank control is subtracted off from the MFI of the standard or test well. Thus, the package implements the “Subtract” method in which, the background noise measured by the blank control MFI is subtracted from every observed point of the standard curve before fitting the curve. Subtracting the blank controls may oftentimes result in poor fitness of the standard curves and adversely affect assay output, as illustrated in cartoons showed in Fig 1. Heuristically, the lower asymptote of a curve represents the absence of analyte and shall approximate the MFI produced by the background noise. Subtracting off the background from the curve is equivalent to removing the lower asymptote. Curves without the lower asymptote will have large variances (lower reliability) reflecting the fact that they are poorly fit. In practice, the lower asymptote estimated with a few highly diluted wells including nearly 0 analyte concentrations shall correspond to the background noise (absence of analyte). The observed MFI in the blank control well in a plate shall be equal to the true MFI of the background signal (expected to be 0 in an unbiased assay) plus a random error. The random error of the background signal in a given plate cannot be assumed to be the same of the random error in other wells containing more concentrated samples, particularly in a non-linear function. Subtracting the MFI of the blank control well from all other wells may add and not subtract error from the assay because it relies on an assumption that the random error was the same in all plate wells. Moreover, this subtraction for values close to the asymptote level may generate negative MFIs that after logarithm transformation will be excluded from the curve fitting. The actual assay data cannot be lower than 0.

In the second method, “Ignore”, the background is ignored, and therefore the blank controls are not taken into account when fitting the standard curve. This allows the left tail of the curve, when the dilution of the reference sample is very high, to asymptotically approach the true background value. Adjustments for assay background in this case, are handled by the lower limits of quantification of the assay. In the third method, “Include”, the background is included as another point when fitting the standard curve using the GM of the blank controls. The expected concentration of the blank control well to be added to the curve is assumed to be equal to the minimum expected concentration value of the standard divided by the dilution factor. This method may result in inappropriately extending the standard curve and may be used in very specific circumstances since it is fully dependent on the blank controls characteristics. On one hand, if the blank controls are contaminated, obtaining an abnormally large value, for example, the lower asymptote estimation of the standard curve will be totally biased (getting a poorer standard curve). On the other hand, if there are not enough dilutions of the standard samples, the extra standard sample using the background information will influence data similarly to an outlier due to the fact that the standard points have not reached the lower asymptote. Therefore, this method is especially useful when the standard curves have enough dilutions and the blank controls are reliably measured. Finally, the fourth implemented method, “Constraint”, consist in constraining the lower asymptote of the model to the background noise. In this last method one less parameter will be estimated (*c* parameter for the five and four-parameters log-logistic functions and *y*_*0*_ for the exponential growth function).

### Plots and statistics useful for QA/QC

Calling the R generic *plot* function to a *scluminex* object creates a standard curve or residuals plot either for all analytes or for one specific analyte. When plotting the standard curve, depending on the user’s specification, the confidence interval or the prediction interval can be shown. The confidence interval corresponds to the prediction interval of the regression and is estimated using all wells of the standard reference of a plate using the t-Student distribution. While reliable curves with narrow confidence intervals have all points lying on or close to the fitted curve, unreliable curves have large intervals and observed values are more distant from the curve. The plot also includes the GM of the blank controls if background information was given to the *scluminex* function.

If after examining the appropriate statistics the user identifies an outlier among the observed data points and decides to flag it so that the point is excluded from the curve fitting, the function includes the flagged point in the plot (as an empty circle) although the point is not used in the estimation of the plotted curve. This way all steps in the QA/QC process are recorded. An important feature of the package is to provide an objective assessment to judge points of the standard curve that may result in poor fitness because of mistakes in the assay. Generally, decisions about outlying observations are based on subjective assessment of investigators. In the package, the standardized residuals of the curve can be plotted as a function of the fitted values and points beyond a user-specified cutoff are marked with the well position in order to easily identify outliers. The function *get_outliers* allows identifying residuals that are beyond a specified cutoff and all information related to this point is shown (analyte, plate and well). Moreover, a normal quantile-quantile plot of the standardized residuals can also be obtained. Additional functions of the package to support QA/QC are the *get_outliers* function and the *intra_icc* function.

### Methods to estimate the limits of quantification (LOQ)

The methods implemented for estimation of the LOQs are based on standard curves. Heuristically, ideal LOQs would restrict the quantification of concentration to samples with MFI within the portion of the standard curve that is “approximately” linear, since this is the portion of the curve in which mapping of MFI to concentration can be done more reliably. In the extreme, MFIs located at the asymptote of the curves cannot be mapped to a unique concentration value. The three approaches included in the package have been described in the literature [12–14] and are called: “Derivative”, “Interval”, and “Coefficient of Variation”. Each method focuses on a specific aspect of the standard curve.

The Derivative method is based on the second order derivative of the standard curve model [12]. The maximum and minimum values of the second order derivative are the HLOQ and LLOQ, and are estimated through the roots of the third derivative. The package calculates the derivatives analytically for the three models implemented to fit standard curves.

The Interval method is based on the coefficients associated with the asymptotes of the models [13]. The LLOQ is the concentration value at the intersection of the lower boundary of the prediction interval of the standard curve and the upper boundary for the confidence interval for the coefficient of the lower asymptote estimated by the model (if this confidence interval is estimable). Similarly, the HLOQ is the concentration value at the intersection between the upper boundary of the prediction interval of the standard curve and the lower boundary of the confidence interval for the coefficient of the upper asymptote estimated by the model (if this confidence interval is estimable). The level of confidence can be specified by the user.

The Coefficient of Variation method is based on the estimation of the coefficient of variation of the fitted concentration values [14,15]. Using the Delta method, the package estimates the standard error (SE) of the fitted concentration conditional on the observed MFI [16], and then calculates the coefficient of variation (CV) as

$$\sqrt{e^{{\left(SE\left(x\right)\text{ln}\left(10\right)\right)}^{2}-1}}$$

where *SE(x)* is the standard error of the fitted concentration. For a specific coefficient of variation cutoff, the LLOQ and HLOQ are calculated as the fitted concentration values whose coefficient of variation is lower or equal to the specified cutoff (by default 20%).

In practice, the Derivative method constrains the quantification of concentrations through the standard curve to the nearly linear part of the curve. The Interval method constrains the LOQ to values that are statistically significantly different from the asymptote values. The Coefficient of Variation method results in LOQs with variability lower than the specified CV. The choice among methods depends on the assay and the analyte. Ideally one wants to choose an approach that allows quantitating the maximum number of samples while keeping the LOQs of all plates above the background noise and limits of detection (LODs). A more detailed documentation of all these features with additional examples and figures of all functions are included in the vignette of the package (https://CRAN.R-project.org/package=drLumi).

### Package availability and requirements for installation

The drLumi package itself is available as supporting file (S2 File). In addition, it is freely available at https://CRAN.R-project.org/package=drLumi. The package requires the R software version 3.2.2 or above and some additional packages that will be installed with the package, if the user does not have them installed in their R environment. Additional instructions about installation and use of the package can be found at the URL: https://cran.r-project.org/web/packages/drLumi/vignettes/drLumi.pdf

## Results and discussion

**Fig 2:**
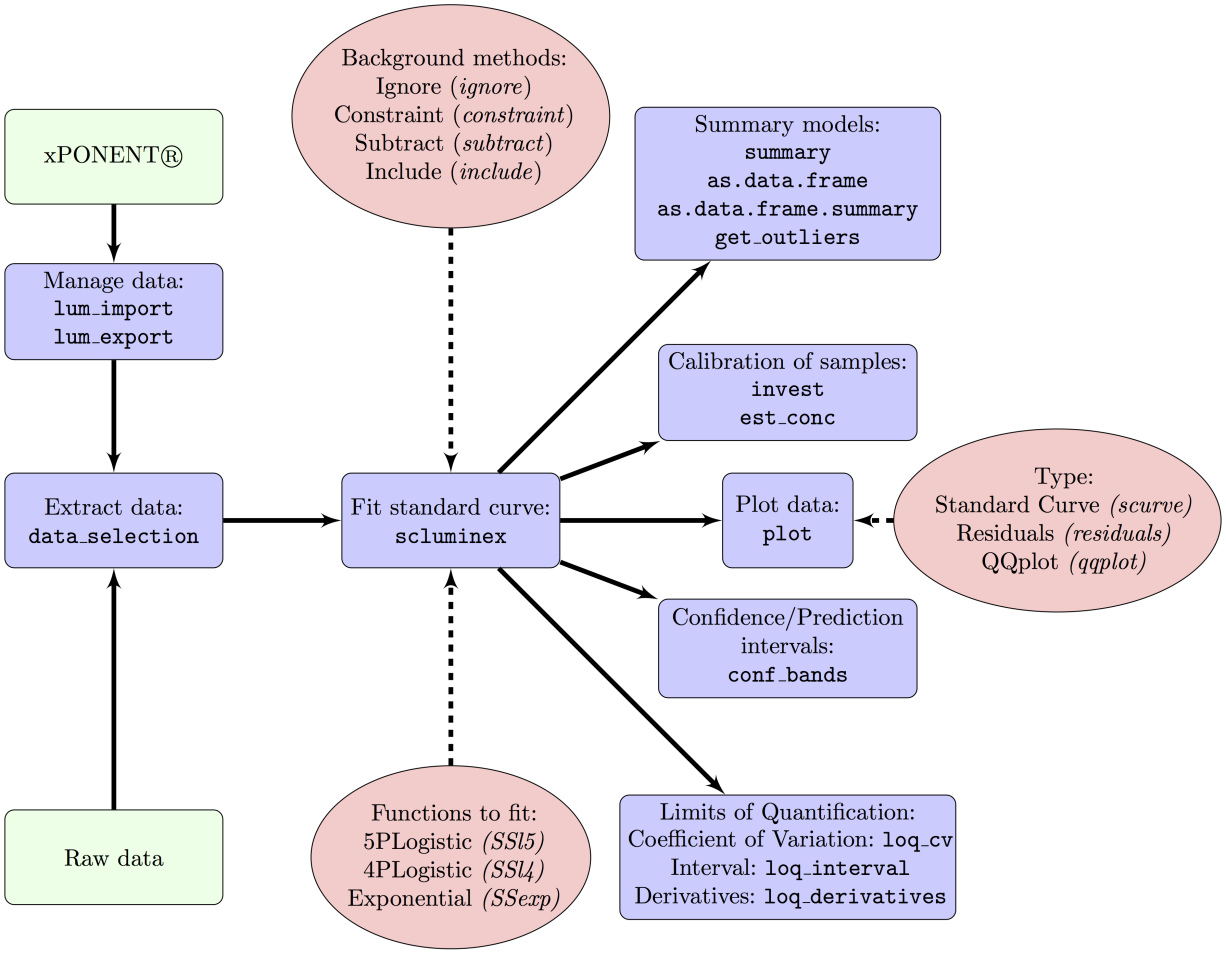
Schematic representation of the functions and arguments included in the drLumi package. The names of the functions are shown inside blue boxes, options and names of arguments of the functions are shown inside red ellipses. The green boxes show the starting points of the flow depending on the origin of the raw data.

**Fig 3:**
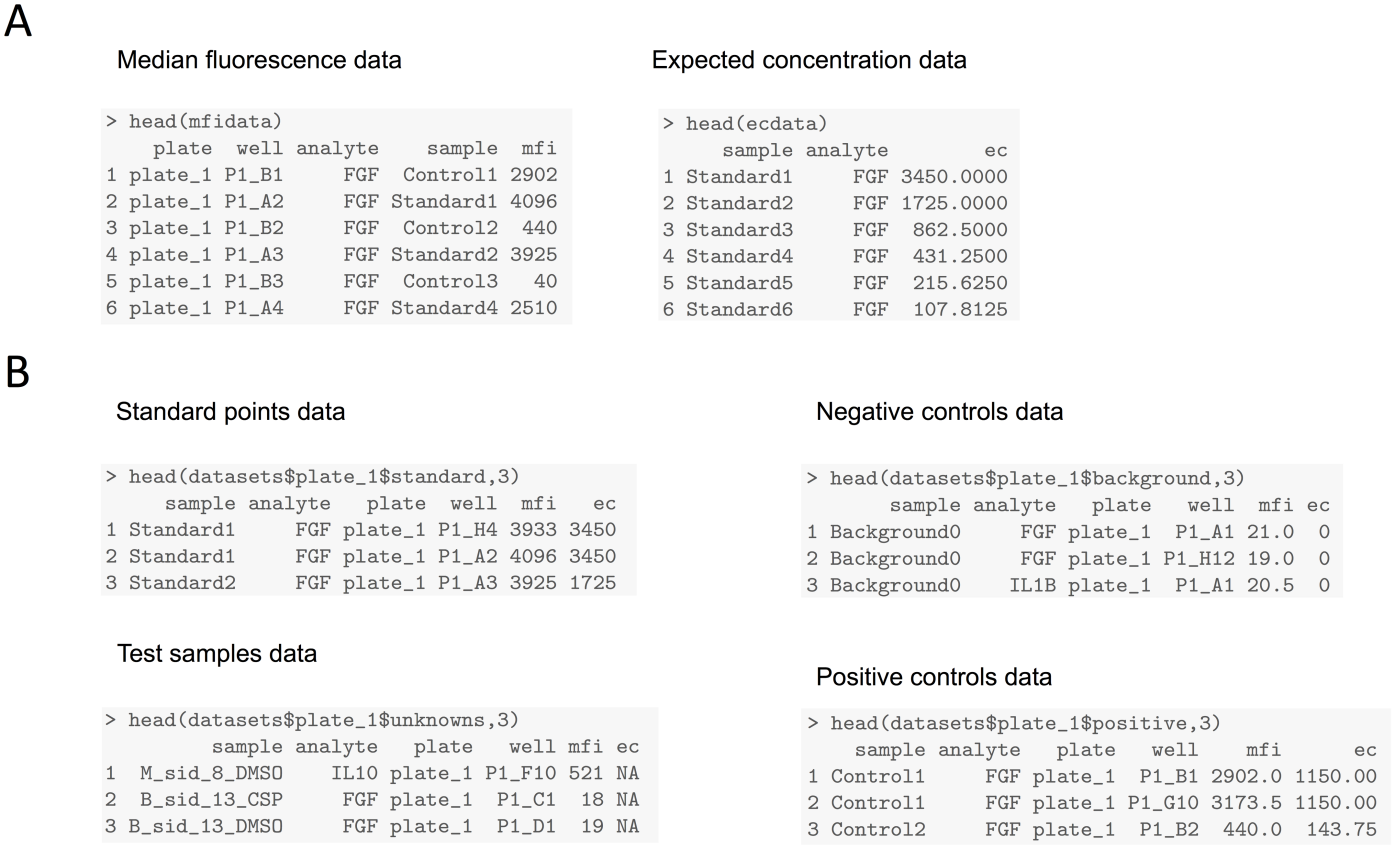
Format of the input dataset used by the package. (A) Dataset user can create for the package; (B) package automated data management: datasets produced by the package when data is automatically extracted from the assay machine.

Fig 2 shows the functions and methods implemented in the package. The main function of the package, *scluminex*, is used to fit the calibration curve and allows the estimation of the concentration of the test samples. This function requires a dataset with MFI and expected concentration of the standard samples organized in the following columns: the identification of the plate/experiment; the well position in the plate; the name of the analyte; the sample identification; the MFI; and the expected concentration. The *scluminex* function has default names for all the columns but the user can specify the name depending on the dataset (Fig 3A). Optionally, another dataset with the negative controls can be used to include their information when estimating the calibration curve. The columns of this dataset should have the same names as the standard dataset (Fig 3B). As explained in the methods, the user can provide to *scluminex* the data adequately pre-formatted or the package can automatically generate the necessary data for *scluminex* through use of *lum_import*, when the software used in the device that generates data is the xPONENT^®^ 3.1.

The function *scluminex* can fit standard curves based on the three parametric nonlinear least squares models described above [11]: 5-parameter LL, 4-parameter LL, and 2-parameter exponential growth. Although the parameterization of the models is in logarithm scale, as described previously, the user should input MFIs and theoretical concentrations in the original scale. The package fits these three models sequentially. For example, first a 5-parameter LL model is fit that may not converge, i.e., the numerical algorithm cannot attain a maximum for the optimization function and estimate parameters. In case of non-convergence, the 4-parameter LL is automatically fitted (and the user is informed about the non-convergence and change of model); if the 4-parameter LL also does not converge, the exponential growth model is automatically fitted. Therefore, models across analytes can differ. The user can change this default option and specify the order of the models to be fitted (if more than one is specified) or specify only one model that will be used for all data. It is arguable, however, if a user would want to specify a single model to fit all data that it could add error to the data. For instance, if standard curves of a given analyte in 10 plates are fit and in 7 of them the 5-parameter function converged and in the remaining 3 of them only the 4-parameter LL4 converged (or even the exponential model converged), the user could re-fit the standard curve of the 10 plates using the simpler model by setting an argument in the function (*lfct*), i.e., the 4-parameter (or the exponential model). However, in doing so the user is unnecessarily using a “worse” standard curve to calibrate the assay in the first 7 plates in which the 5-parameter LL5 converged and potentially not calibrating so well the test samples. Additional arguments to allow users to customize the assay are available including some characteristics of the nonlinear least squares algorithm, such as, the maximum number of iterations allowed.

**Fig 4:**
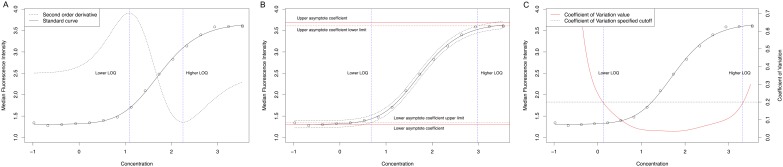
Estimation of the limits of quantification (LOQ), based on the “Derivative” (A), the “Interval” (B), and the “Coefficient of Variation” methods (C). In A solid and dashed black lines show the standard curve and the second order derivative, respectively. Dashed blue lines show the limits of quantification. In B, solid and dashed red lines show the asymptote coefficients and the 95% confidence interval, respectively. Dashed blue lines show the LOQ, and dashed black lines show the 95% prediction interval of the standard curve. In C, solid red and black lines show the coefficient of variation (CV) estimated for each fitted concentration and the standard curve, respectively. Dashed blue lines show the LOQ, and the dashed black line shows the user specified CV cutoff.

The coefficients of the standard curves, i.e. the parameters that determine the sigmoidal fit by analyte (for example, the lower and upper asymptotes, the slope and the halfway value between asymptotes in a four-parameters logistic function), with the corresponding standard errors and t-tests to assess whether the coefficients are different from zero can be summarized for each analyte applying the *as*.*data*.*frame* function to a *summary*.*scluminex* object. These statistics may help investigators to assess the fitness of the curve. For instance, curves with slope not statistically significantly different from 0 may be flat and suggest problems with the specific plate. Other useful statistics to assess the fitness of the curve and perform QA/QC of the assay are calculated: adjusted R^2^ [17], the Neill test [18] (which is an ANOVA-based lack-of-fit test) and the Akaike information Criterion (AIC) [19]. An additional function of the package to support QA/QC is the *intra_icc* function that estimates the intraclass correlation coefficient, for instance, to assess reliability across operators. This function calls the *icc* function from the irr package [20] and all arguments of this function can be specified. Estimation of LOQs using the three different approaches is illustrated in Fig 4.

To demonstrate the use of the drLumi package, a toy dataset based on real data from a study about correlates of protection from malaria with the RTS,S/AS01E malaria vaccine has been analyzed [21]. This trial is registered with ClinicalTrials.gov, number NCT00866619. The figures and statistics were generated with the package (code available in the package *inst/extdata/toydata*.*R* folder). Four analytes were created based on a 4-parameter LL model emulating a real study assay to exemplify different scenarios. Therefore, each generated analyte is exemplifying a potential scenario that can be found in a real multiplex immunoassay in which one antigen can present outliers and the others do not. The four scenarios (analytes) were analyzed using the drLumi package, in a single dataset as in usual multiplex immunoassays. Two blank controls were used for the estimation of the background noise. Although drLumi could fit standard curves using any number of dilutions determined by the number of data-points specified for the curves in the dataset or the plate map, the assays that motivated the toy data had curves with 16 serial dilutions. Therefore, the toy example had 16 serial dilutions (1:2) and the first dilution (highest concentration) was duplicated (thus, a total of 17 points).

**Fig 5:**
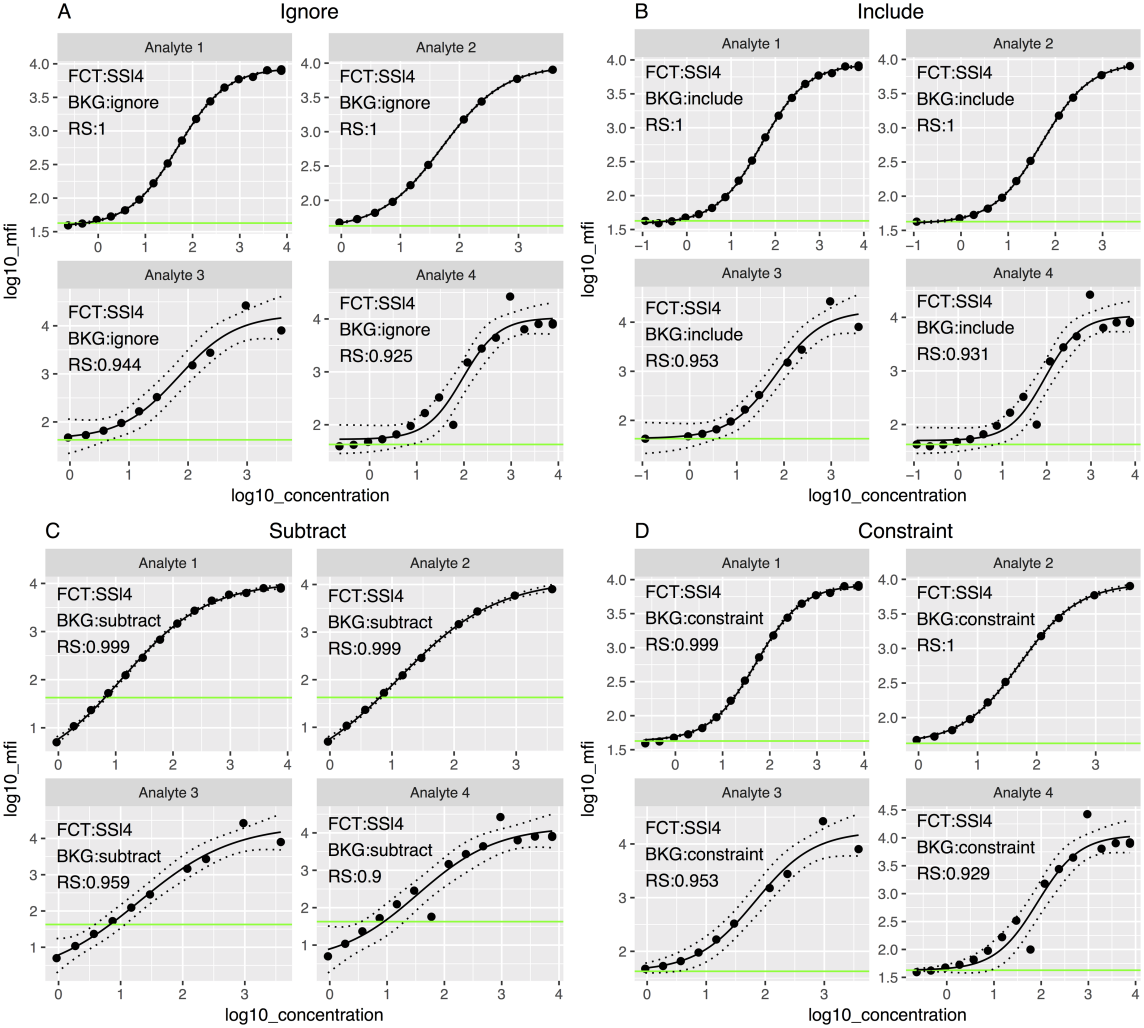
Standard curves based on the four-parameter log-logistic model (FCT) fitted using the four alternative approaches to treat background noise (BKG) in the four emulated datasets. The dashed lines represent the confidence interval of the curve, and the green line represents the geometric mean of the blank controls. RS = R-square. (A) Standard curve fitted using “Ignore” background noise method; (B) standard curve fitted including the background noise as an extra point when fitting the curve, using “Include” background method; (C) standard curve fitted subtracting the background noise using the “Subtract” method; (D) standard curve fitted constraining the lower asymptote to the background noise using the “Constraint” method.

In the first scenario (Analyte 1, Fig 5A), all 17 points were used in the fit of the curve; there were no outliers. In the second scenario (Analyte 2), instead of observing 17 points, only 10 points were analyzed. In the third scenario (Analyte 3), only 10 points were observed and 1 of those was an outlier that was created by randomly assigning a standard deviation to these points two times larger than the standard deviation of the original data. In the fourth scenario (Analyte 4), all 17 points were observed but 2 of those were outliers. The four-parameter log-logistic function was fitted for all scenarios. The blank controls in the four scenarios were obtained from the same real assay.

**Fig 6:**
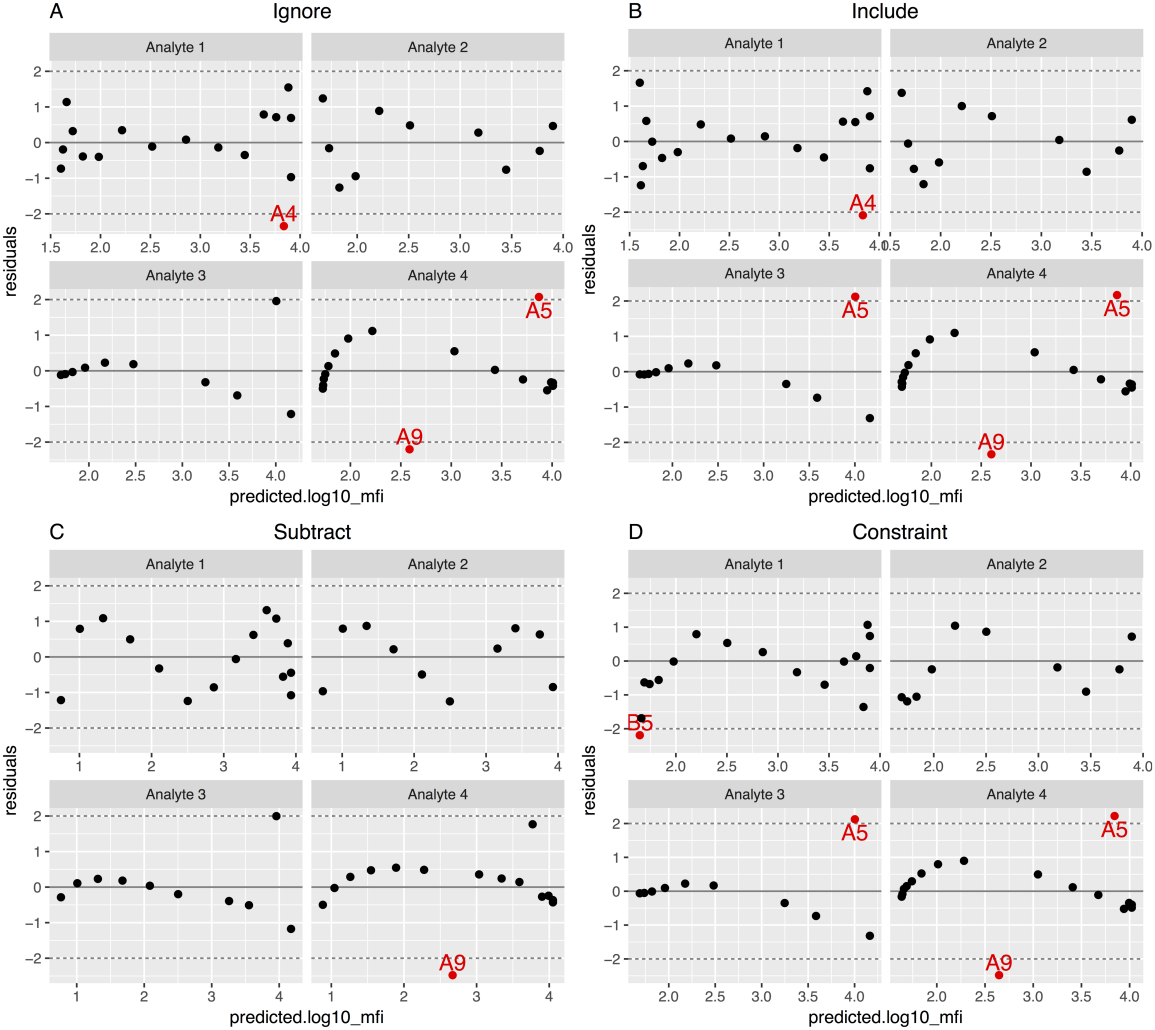
Standardized residuals as a function of the predicted base 10 logarithm median fluorescence intensity (MFI) for the four emulated datasets based on standard curves fitted using the four different approaches to treat background noise. Points outside of the range specified by the dashed lines are in red and labeled according to the well location in the 96-well plate. (A) Standard curve fitted using “Ignore” background noise method; (B) standard curve fitted including the background noise as an extra point when fitting the curve, using “Include” background method; (C) standard curve fitted subtracting the background noise using the “Subtract” method; (D) standard curve fitted constraining the lower asymptote to the background noise using the “Constraint” method.

**Fig 7:**
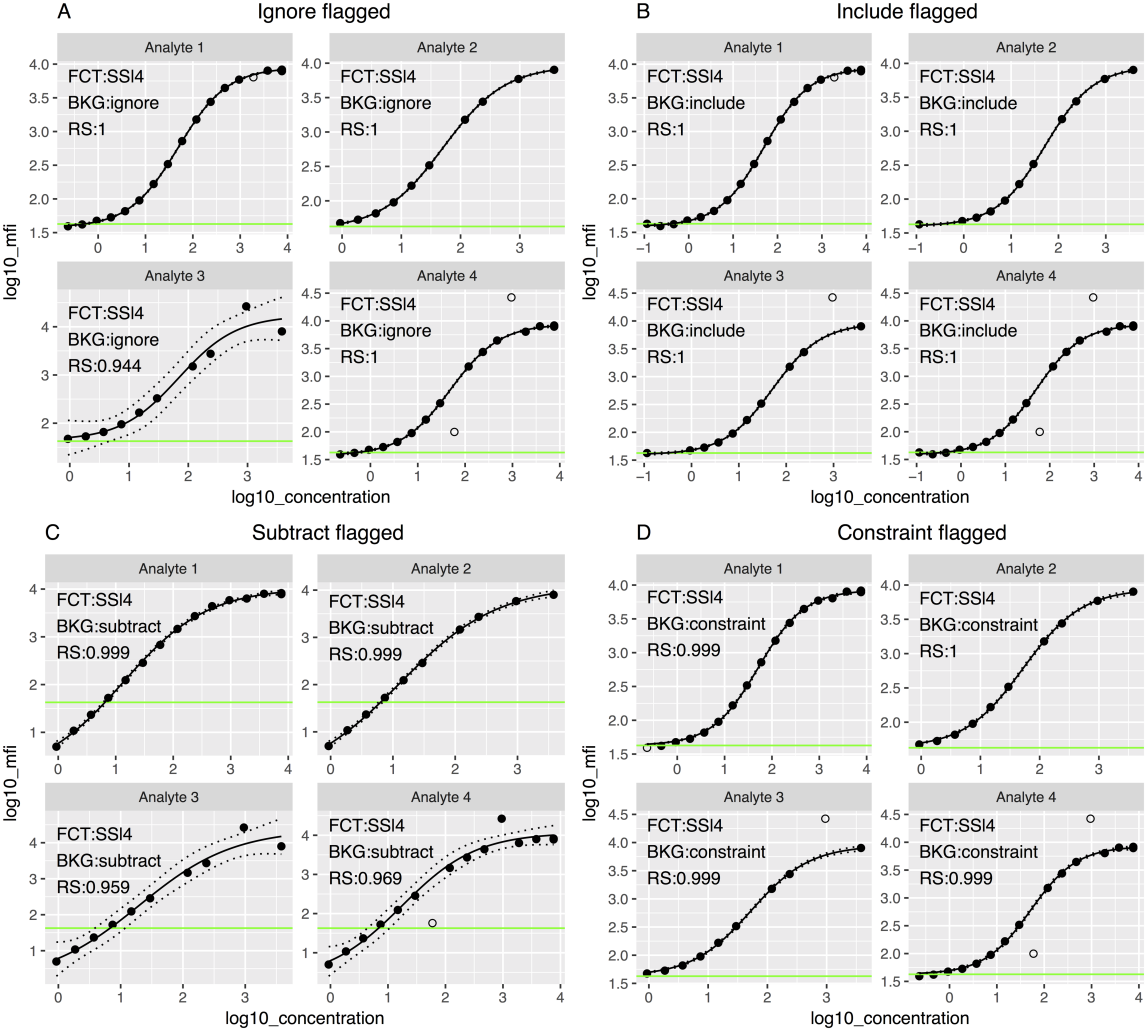
Standard curves based on the four-parameter log-logistic model (FCT) fitted using the four alternative approaches to treat background noise (BKG) in the four emulated datasets after the outliers identified in Fig 5 were flagged (empty circles). The dashed lines represent the confidence interval of the curve, and the green line represents the geometric mean of the blank controls. RS = R-square. (A) Standard curve fitted using “Ignore” background noise method; (B) standard curve fitted including the background noise as an extra point when fitting the curve, using “Include” background method; (C) standard curve fitted subtracting the background noise using the “Subtract” method; (D) standard curve fitted constraining the lower asymptote to the background noise using the “Constraint” method.

**Fig 8:**
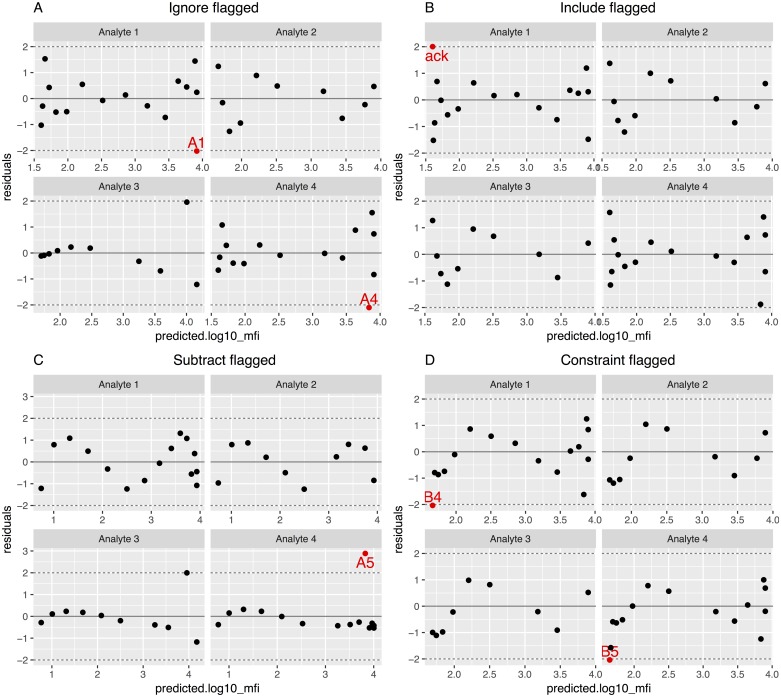
Standardized residuals as a function of the predicted base 10 logarithm median fluorescence intensity (MFI) for the four emulated datasets based on standard curves fitted after outliers were flagged and using the four different approaches to treat background noise. Points outside of the range specified by the dashed lines are in red and labeled according to the well location in the 96-well plate. (A) Standard curve fitted using “Ignore” background noise method; (B) standard curve fitted including the background noise as an extra point when fitting the curve, using “Include” background method; (C) standard curve fitted subtracting the background noise using the “Subtract” method; (D) standard curve fitted constraining the lower asymptote to the background noise using the “Constraint” method.

For each scenario, the four background methods were fitted (Fig 5A–5D) and the standardized residuals plots (Fig 6A–6D) obtained. In all cases, points with absolute value larger than two were considered outliers. In the Analyte 3 scenario, there was a point that visually seemed an outlier but had a residual lower than 2 in all methods except in the Include and Constraint, where the point was identified as an outlier. After identifying the outliers and generating a flag variable into the dataset with the *get_outliers* function, we fitted and plotted again the curve with the *scluminex* and the *plot*.*scluminex* functions to generate the same plots with the flagged points. Curves (Fig 7A–7D) and standardized residual plots (Fig 8A–8D) were fitted using the four approaches to account for background noise. The flagged points are identified in the standard curve plot as non-filled points. The residual plots of the newly fitted curves showed new points with residual larger than the cutoff value of two. For the Include background method the GM of the two blank controls was identified as an outlier for Analyte 1.

Summary statistics before and after exclusion of outliers for the Subtract method were generated using the generic R function *summary* and *as*.*data*.*frame* to a *scluminex* object (Table 1). Using this method, points with lower MFIs than background were automatically excluded because they obtained negative MFIs; therefore, 15 points for Analyte 1 and Analyte 4 were analyzed. AIC and Goodness-of-Fit statistics (Neil test p-value) were automatically estimated by the package but AIC should be compared across same datasets (same analytes and same number of observations).

**Table 1:** Comparison of automatically generated results provided by the package before/after flagging outliers for the Subtract background method for the simulated analytes. In Analyte 1 no outliers and no missing values were included, in Analyte 2 seven missing values were included, in Analyte 3 one outlier and seven missing values were included and in Analyte 4 two outliers were included in the original simulated data. Only in Analyte 4 outliers were flagged by the package and their removal changed results. For Analyte 4, the limits of quantification for the coefficient of variation method could not be estimated with all data due to the fact that the minimum coefficient of variation value was larger than the specified 30% cutoff.

Subtract Blank control	Analyte 1	Analyte 2	Analyte 3	Analyte 4 Before /After
Standard points, n	15	10	10	15 / 14
R^2^	1.00	1.00	0.96	0.90 / 0.97
Akaike Information Criteria	-51.6	-35.3	4.1	18 / 0.9
Neill test p value	0.05	0.10	0.40	0.17 / 0.18
Outliers detected, n	0	0	0	1 / 1
Derivative method				
LOQ, log 10 scale	(0.16;2.1)	(0.05;2.14)	(0.29;2.29)	(0.61;2.44) / (0.38;2.09)
Dynamic range, log 10 scale	1.94	2.09	2	1.81 / 1.71
Interval method (95% CI)				
LOQ, log 10 scale	(-0.03;3.88)	(-0.03;3.58)	(1.63;1.68)	(1.63;2.11) / (1.1;2.00)
Dynamic range, log 10 scale	3.91	3.61	0.05	0.48 / 0.9
Coefficient of Variation method (30% cutoff)				
LOQ, log 10 scale	(-0.03;3.56)	(-0.03;3.46)	(0.34;1.97)	-- / (0.31;2.11)
Dynamic range, log 10 scale	3.59	3.49	1.63	-- / 1.81

The LOQs based on the three methods were estimated in this dataset (Table 1); for the Coefficient of Variation method the specified cutoff was 30%, although oftentimes a cutoff of 20% or less may be desirable. For the Interval method the 95% level of confidence was used. The dynamic ranges (difference between HLOQ and LLOQ) are shown. The LOQ could not be estimated before flagging outliers for Analyte 4 because the minimum coefficient of variation obtained from all fitted concentration range was greater than 30% so all coefficients of variation were above the specified cutoff. Of note, the package estimates LOQs specific to a plate and the standard curve of the plate instead of estimating a single LLOQ and HLOQ applicable to the entire assay. Standard curve shape and range parameter estimates may vary across plates and this variation is what allows curves to be used to calibrate random plate-to-plate variability of a test sample. Background signal of blank controls also vary with the same factors that affect standard curve variability. Therefore, when estimating LOQs based on standard curve, we believe that the concentrations of an analyte in a test sample that can be reliably quantitated can be better estimated through the range and the variability of the curve in the plate the test sample is included. For instance, a plate with larger assay variability shall have a standard curve with larger variance. Therefore, in this plate, a CV of 20% will correspond to a higher threshold of MFI or concentration than the threshold in a plate with low variability in which standard curves presented narrow variability of the standard curve and its parameters.

Part of a QA/QC report using results generated by the package based on real multiplex immunoassay experiment is shown in Supporting Information (S3 File). In this experiment, a panel with 30 analytes was analyzed and all information generated by the package was used: summary of coefficients, standard curves figures, residuals and limits of quantification.

## Conclusions

The drLumi package implements several tools to pre-process multiplex immunoassay data. The package includes alternative methods to account for background noise (estimated by blank controls) when fitting the standard curves, to estimate the LOQ, and to automate QA/QC. Additionally, the function helps the analyst to automatically read data generated by xPONENT^®^ 3.1 software. These features result in a faster and more adequate data pre-processing, and as a result, more powerful and accurate analysis of biomarkers, correlates of protection or any other analysis of association based on multiplex bead immunoassays. Although the package is particularly tailored to assays based on Luminex systems technology it can be easily expandable in the future to analyze data of other technologies, such as Somascan assays. We are permanently maintaining the package and plan a new release with a more user-friendly interface so that laboratory investigators not acquainted with the R software could easily use the package functionality. The package can replace with advantages other packages commercially available distributed by companies that produce multiplex bead based assays.

## Supporting information

S1 File Example of a raw data file generated by the xPONENT^®^ software and importable by the drLumi package. (CSV)

S2 File The drLumi package. (TAR)

S3 File Example of quality assurance/quality control (QA/QC) report developed using drLumi when monitoring the conduct of a multiplex bead immunoassay analyzing samples from a study about correlates of protection in malaria. (PDF)
