# Supplementary material for: drLumi: An open-source package to manage data, calibrate, and conduct quality control of multiplex bead-based immunoassays data analysis
Source: PLoS One. 2017 Nov 14;12(11):e0187901. doi: 10.1371/journal.pone.0187901 (PMC5685631; doi:10.1371/journal.pone.0187901)
Supplement: S2 File — (TAR) [file pone.0187901.s002.tar › drLumi/inst/doc/drLumi.pdf]

drLumi: multiplex immunoassays data analysis

Héctor Sanz[1], John Aponte[1], Jaroslaw Harezlak[2],
Yan Dong[2], Magdalena Murawska[2] and Clarissa Valim[3]

September 23, 2015

[1]ISGlobal, Barcelona Ctr. Int. Health Res. (CRESIB), Hospital Clínic - Universitat de Barcelona, Barcelona, Spain

[2]Indiana University Fairbanks School of Public Health, Indianapolis, IN, USA

[3]Harvard School of Public Health, Boston, MA, USA

hector.sanz@isglobal.org

Contents

1 Introduction **2**

2 Main structure of datasets **4**

3 Datasets in the package **4**
3.1 MFI data . . . 4
3.2 EC data . . . 4
3.3 Raw data from xPONENT® software . . . 5

4 Extraction of samples data **5**

5 Data analysis **6**
5.1 Models . . . 7
5.2 Blank controls . . . 8
5.3 Limits of quantification . . . 10
5.4 Fitted concentration . . . 13
5.5 Agreement between controls . . . 15

6 Summary of results **16**

7 Flag data **22**

8 Raw data from xPONENT® software **24**
8.1 MFI raw data . . . 24
8.2 Bead raw data . . . 26

1 Introduction

The drLumi package allows users to manage data, calibrate assays and perform quality control of multiplex immunoassays. This document provides an overview on the usage of the main functions of the package and examples of the implemented methods. The datasets available in the package were used in the examples presented so they can be reproduced.

Multiplex immunoassays are used to measure concentrations of several cytokines and chemokines, antibodies, or other proteins simultaneously and are important for biomarkers discovery.

Several beads with antibodies fixed to capture the analyte of interest are mixed with subject's serum or plasma in 96 well plates, one subject per well. Sets of beads are individually labeled according to the specific analyte capability of measuring. After the beads capture the existing analyte in subject's sample, they are allowed to interact with a fluorescent antibody. In the presence of the analyte, the fluorescent antibody and the antibody fixed to the bead make a sandwich with the analyte to be quantified. This complex goes through laser bins that quantify the amount of analyte in each bead with the analyte-antibodies complex; the assay final raw output is the median fluorescence intensity (MFI) over all beads containing the corresponding analytes. To calibrate between-plate variability and quantify analyte concentrations, each plate includes wells containing increasing dilutions of a sample with known concentration of each analyte. Those wells are used to fit standard curves with the MFI as a function of the concentration of the analyte in the reference samples. To calibrate each subject's response, the MFI of the subject samples is converted in analyte concentration using the standard curve. The minimum and maximum concentration of analyte that can be reliably quantified after using the standard curve to transform the MFI in an analyte concentration is called lower and higher limit of quantification (LLOQ and HLOQ), respectively. Additionally, to estimate the background noise in the assay, samples containing no analyte (negative or blank controls) are included in each assay plate.

Several analytical factors when pre-processing the data from the assay may result in suboptimal calibration and decrease assay sensitivity, i.e., failure to quantify a large amount of samples because concentration is outside the limits of quantification (LOQ) range, including the fit of standard curve and the approach used to estimate limits of quantification. Moreover, the approach used to account for background noise when estimating standard curves can also affect the fitting of the standard curves and impact the assay calibration and sensitivity.

There are several commercial software to handle concentration-response multiplex data. Usually these software allow to estimate concentration of samples using a standard curve but they don't allow to perform a good Quality Control (QC) based on residuals, goodness of fit or confidence intervals for the coefficients.

In the following sections we illustrate how to perform all QC process for multiplex immunoassays data using `drLumi` package.

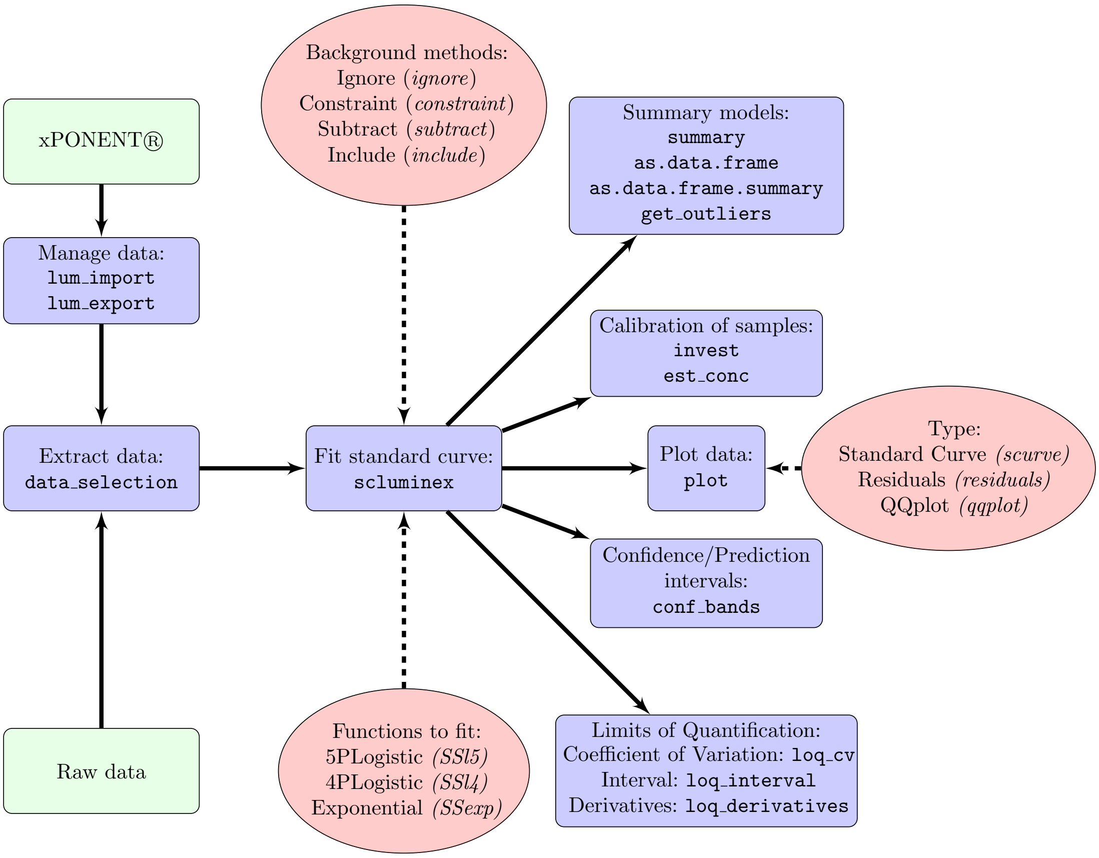

Figure 1: Schematic representation of the main functions and arguments included in the drLumi package. The name of the functions are shown inside blue boxes, options and name of arguments of the functions are shown inside red ellipses and the green box shows the starting point of the flow depending on the origin of the raw data.

The package allows to import CSV raw data that has been exported from xPONENT® software. For a detailed example showing how to process this type of data go to section 8.

To load the package:

```
> library(drLumi)
```

2 Main structure of datasets

The package only allows to read automatically CSV files from xPONENT® software. Other data must be pre-processed, if necessary, and transformed into a `data.frame` class object. The final `data.frame` must have the following variables:

- Plate identification.
- Well position.
- Analyte name.
- Sample identification.
- Median fluorescence intensity values.
- Expected concentration.

The information can be just in one `data.frame` or in several (e.g., `mfidata` and `ecdata`).

3 Datasets in the package

There are three datasets available in the `drLumi` package.

3.1 MFI data

A dataset with the median fluorescence intensity values for three 96-wells plates and 30 analytes information per plate. The variables included in the dataset are:

- plate: plate identification.
- well: position of the sample in the plate.
- analyte: analyte tested.
- sample: type of sample in the well (blank, standard, positive control or unknown). In each plate 2 blanks, 17 dilution points (standard 1 is duplicated), 3 controls (each one duplicated) were tested.
- mfi: Median Fluorescence Intensity.

To load the median fluorescence data:

```
> data(mfidata)
```

First 6 rows of the `mfidata`:

```
> head(mfidata)
    plate  well analyte    sample  mfi
1 plate_1 P1_B1     FGF  Control1 2902
2 plate_1 P1_A2     FGF Standard1 4096
3 plate_1 P1_B2     FGF  Control2  440
4 plate_1 P1_A3     FGF Standard2 3925
5 plate_1 P1_B3     FGF  Control3   40
6 plate_1 P1_A4     FGF Standard4 2510
```

3.2 EC data

A dataset with the expected concentration data by analyte for the `mfidata`. The variables included in the `data.frame` are:

- sample: type of sample (background, control or standard).

- analyte: analyte tested.
- ec: expected concentration value.

To load the expected concentration data:

```
> data(ecdata)
```

First 6 rows of the `ecdata`:

```
> head(ecdata)
     sample analyte        ec
1 Standard1     FGF 3450.0000
2 Standard2     FGF 1725.0000
3 Standard3     FGF  862.5000
4 Standard4     FGF  431.2500
5 Standard5     FGF  215.6250
6 Standard6     FGF  107.8125
```

3.3 Raw data from xPONENT® software

There is an example of CSV raw data from xPONENT® software in `inst/extdata` named `plate1.csv`. For a detailed example of this data go to section 8.

4 Extraction of samples data

Generally, the machine that reads the assay is connected to a software that produces a dataset including standard points, blank controls and subject's samples. The `data_selection` function allows splitting variables and samples in different datasets. Also, is possible to merge the expected concentration values and flag points (samples to be removed from the analysis, for instance outliers). Two available methods can be used in order to identify the samples:

- specify the *pattern* of the samples' name. The `data_selection` function using regular expressions will match samples based on the *pattern*.
- specify the exact name of the samples.

Extracting samples based on a *pattern* and merging the expected concentration variables:

```
> datasets <- data_selection(x = mfidata, ecfile = ecdata,
+     byvar.ecfile = c("sample","analyte"),
+     backname = "Background0",
+     stanname="Standard",posname = "Controls")
```

Where

- `x=mfidata`: is the MFI `data.frame`.
- `ecfile=ecdata`: is the expected concentration `data.frame` to be merged to `mfidata`.
- `byvar.ecfile = c("sample","analyte")`: are the link variables between the `mfidata` and `ecdata`.
- `backname = "Background0"`: is the pattern of the blank controls.
- `stanname = "Standard"`: is the pattern of the standard points.
- `posname = "Controls"`: is the pattern of the controls other than blank.

All samples datasets have been extracted (first 3 observations of each):

Background

```
> head(datasets$plate_1$background,3)
       sample analyte   plate    well  mfi ec
1 Background0     FGF plate_1  P1_A1 21.0  0
2 Background0     FGF plate_1 P1_H12 19.0  0
3 Background0    IL1B plate_1  P1_A1 20.5  0
```

Standard

```
> head(datasets$plate_1$standard,3)
     sample analyte   plate  well  mfi   ec
1 Standard1     FGF plate_1 P1_H4 3933 3450
2 Standard1     FGF plate_1 P1_A2 4096 3450
3 Standard2     FGF plate_1 P1_A3 3925 1725
```

Positive controls

```
> head(datasets$plate_1$positive,3)
    sample analyte   plate   well    mfi      ec
1 Control1     FGF plate_1  P1_B1 2902.0 1150.00
2 Control1     FGF plate_1 P1_G10 3173.5 1150.00
3 Control2     FGF plate_1  P1_B2  440.0  143.75
```

Unknowns

```
> head(datasets$plate_1$unknowns,3)
          sample analyte   plate   well mfi ec
1  M_sid_8_DMSO    IL10 plate_1 P1_F10 521 NA
2  B_sid_13_CSP     FGF plate_1  P1_C1  18 NA
3 B_sid_13_DMSO     FGF plate_1  P1_D1  19 NA
```

5 Data analysis

The function used to analyze the data is `scluminex`. Given standard and background (optional) datasets, a list of nonlinear models and a background method this function tries to fit the list of models hierarchicaly. The package has some pre-specified models. The models are fitted by the `nlsLM` function from the `minpack.lm` [2] package which is a modified version of the `nls` function that incorporates the Levenberg-Marquardt algorithm. The `scluminex` function transforms the original data into base 10 logarithm.

```
> allanalytes <- scluminex(plateid = "newplate",
+     standard = datasets$plate_1$standard,
+     background = datasets$plate_1$background,
+     bkg = "ignore", lfct = c("SSl5","SSl4"),
+     fmfi = "mfi", verbose = FALSE)
```

where,

- `plateid`: is the name of the plate (or experiment).
- `standard = datasets$plate_1$standard`: is a `data.frame` with the standard points information.
- `background = datasets$plate_1$background`: is a `data.frame` with the blank controls data.
- `bkg = "ignore"`: is the approach to account for the background noise.
- `lfct = c("SSl5","SSl4")`: are the models to be fitted. The function will try to estimate in first place the `SSl5` model and if the model does not converge will try to fit `SSl4`.
- `fmfi = "mfi"`: is the name of the MFI variable.
- `verbose = FALSE`: logical to do not print the convergence of the models.

Note that the class of `allanalytes` is `scluminex`. The `list` syntax can be used to extract the information of one specific analyte:

```
> class(allanalytes)
[1] "scluminex"
> allanalytes
 [1] "FGF"     "IL1B"    "G-CSF"   "IL10"    "IL13"    "IL6"     "IL12"
 [8] "RANTES"  "EOTAXIN" "IL17"    "MIP1A"   "GMCSF"   "MIP1B"   "MCP1"
[15] "IL15"    "EGF"     "IL5"     "HGF"     "VEGF"    "IFNg"    "IFNa"
[22] "IL1RA"   "TNFa"    "IL2"     "IL7"     "IP10"    "IL2R"    "MIG"
[29] "IL4"     "IL8"
> names(allanalytes$FGF)
 [1] "convergence"  "data"         "model"        "coefficients" "rsquare"
 [6] "aic"          "modelfit"     "neill.method" "flag_data"    "fct"
[11] "bkg_mean"     "alertbkg"     "bkg_method"   "fieldnames"
```

5.1 Models

As described previously, the package has implemented some nonlinear `selfStart` models:

Five-parameter logistic function: using a base 10 logarithm is implemented though the `SSl5` function:

$$f(x; b, c, d, e, f) = c + \frac{d - c}{(1 + 10^{b(x-e)})^f}$$

where,

- b: the slope around e parameter
- c: the lower asymptote parameter
- d: the upper asymptote parameter
- e: the concentration that produces a response halfway between c and d.
- f: the asymmetry for the slope

The constraint version has c as a fixed value instead a parameter.

Four-parameter logistic function: is expressed as a particular version of the five-parameter logistic model when the f parameter is fixed to 1:

$$f(x; b, c, d, e) = c + \frac{d - c}{1 + 10^{b(x-e)}}$$

The `SSl4` function has the `selfStart` model implemented also on base 10 logarithm. As the `SSl5`, the constraint method has the parameter c as a fixed value.

Exponential growth: the `SSexp` function has the `selfStart` exponential model implemented on base 10 logarithm:

$$f(x; y_0, b) = y_0 10^{\frac{x}{b}}$$

where,

- b: the growth rate
- y_0: the response when there is no concentration.

The constraint version of this function has y_0 as a fixed value instead a parameter.

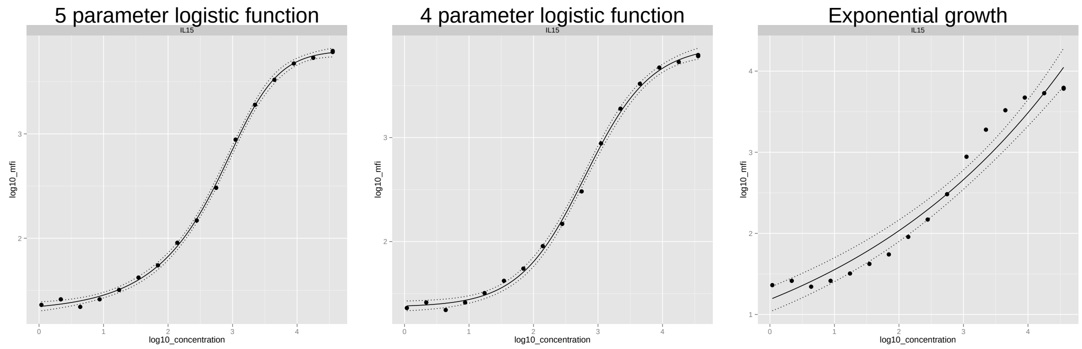

Figure 2: Comparison of five-parameter, four-parameter and exponential models for plate 1 (IL15 analyte) with ignored background.

5.2 Blank controls

The `scluminex` functions allows to specify 4 methods to handle blank controls. As an example, first we subset information from one analyte and the we add the expected concentration data:

```
> fgf <- subset(mfidata, analyte=="FGF" & plate=="plate_1")
> dat <- data_selection(fgf, ecdata)
```

Ignored: the background information can be ignored, so the estimation of the coefficients will not take into account any background data.

```
> ig <- scluminex("plate_1",dat$plate_1$standard, dat$plate_1$background,
+                  lfct="SSl4", bkg="ignore", fmfi="mfi", fanalyte="analyte",
+                  verbose=FALSE)
```

Included: the estimation of the standard curve takes into account the mean of the background of the values as another point of the standard curve. The median fluorescence intensity and the expected concentration for this new point by analyte is estimated as follows:

- MFI: geometric mean value of the blank controls.
- EC: the minimum expected concentration value of the standard points divided by 2.

```
> inc <- scluminex("plate_1",dat$plate_1$standard, dat$plate_1$background,
+                  lfct="SSl4", bkg="include", fmfi="mfi", fanalyte="analyte",
+                  verbose=FALSE)
```

Subtracted: the geometric mean of the blank controls is subtracted from all the standard points.

```
> sub <- scluminex("plate_1",dat$plate_1$standard, dat$plate_1$background,
+                  lfct="SSl4", bkg="subtract", fmfi="mfi", fanalyte="analyte",
+                  verbose=FALSE)
```

Constrained: the constrained parameter (lower asymptote) is fixed to the geometric mean background value and $p-1$ parameters are estimated from the original model.

```
> cons <- scluminex("plate_1", dat$plate_1$standard, dat$plate_1$background,
+     lfct="SSl4", bkg="constraint", fmfi="mfi", fanalyte="analyte",
+     verbose=FALSE)
```

Figure 3 shows the comparison of the 4 methods.

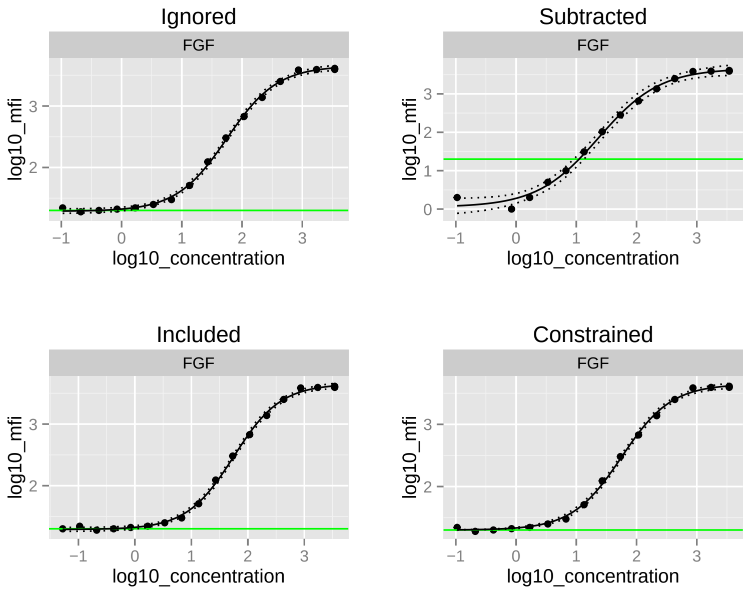

Figure 3: Comparison of background methods for plate 1 (FGF analyte) and 4 parameters logistic model. Green line shows the geometric mean of the blank controls.

5.3 Limits of quantification

The package implements 3 types of estimation for the upper and lower limit of quantification.

Derivatives: the estimation is based on the second order derivative of the model [9]. Once is estimated the maximum and minimum values are found (finding the roots on the third derivative) and those are the limits of quantification (Figure 4). The package calculates the exact derivatives functions for the `SSl5`, `SSl4` and `SSexp`. Given an `scluminex` object the `loq_derivatives` function estimates the LOQ for all analytes or the specified ones by the `subset.list` argument.

```
> # arguments of the function
> args(loq_derivatives)
function (x, subset.list = NULL, ...)
NULL
```

```
> der <- loq_derivatives(allanalytes, subset.list="FGF")
> der
  analyte     lloq     uloq     method
1     FGF 1.088223 2.250466 derivative
```

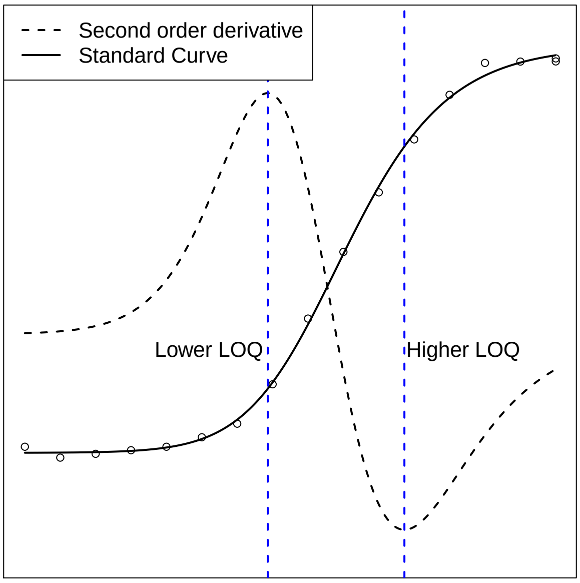

Figure 4: Representation of the limits of quantification based on derivatives.

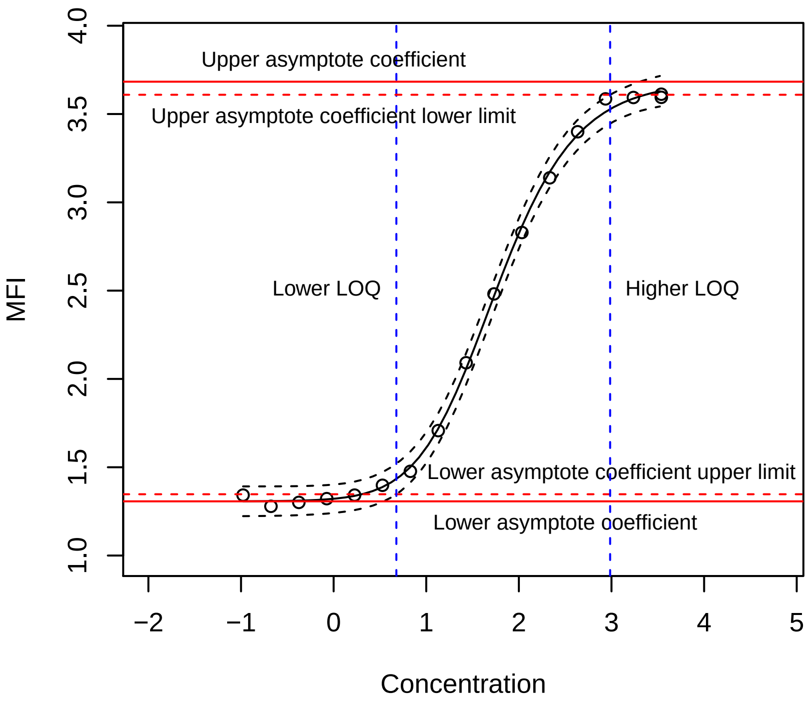

Figure 5: Estimation of limits of quantification based on interval method for plate 1, FGF analyte and ignored background

Interval: the `loq_interval` function estimates the LOQ based on the prediction interval of the curve and the coefficients of the model [7]:

- Lower limit of quantification: is the concentration value of the intersection between lower prediction interval of the standard curve and the upper interval for the lower asymptote coefficient estimated by the model (if the model allows).
- Upper limit of quantification: is the concentration value of the intersection between upper prediction interval of the standard curve and the lower interval for the upper asymptote coefficient estimated by the model (if the model allows).

The function needs as an argument, the model and the position or name of the asymptote coefficients. In Figure 5 there is an example for the plate 1, FGF analyte with ignored background.

```
> int <- loq_interval(allanalytes, subset.list= "FGF", low.asymp=2, high.asymp=3)
> int
  analyte     lloq     uloq   method
1     FGF 0.677414 2.985435 interval
```

In the scenario where one of the asymptotes must be specified and not based in the coefficients, the arguments `lowci` or `highci` must be changed into the desired value. For example, in Figure 6 the upper LOQ is estimated based on coefficients (same values as estimated in Figure 5) but the lower asymptote has been fixed to 1.5 (not based on the coefficients estimation). Therefore, the intersection of the lower asymptote with the prediction interval is different and consequently the estimation of the concentration:

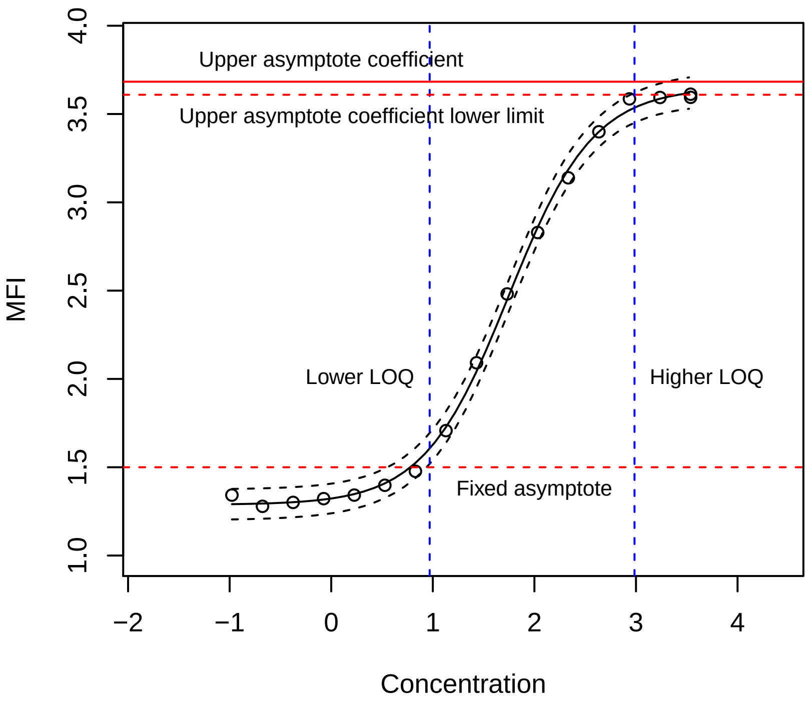

Figure 6: Estimation of limits of quantification based on interval method (fixed lower assymptote value) for plate 1, FGF analyte and ignored background.

```
> int2 <- loq_interval(allanalytes, subset.list="FGF", high.asymp=3, lowci=1.5)
> int2
  analyte      lloq     uloq   method
1     FGF 0.9690558 2.985435 interval
```

Coefficient of variation: this method is based on the estimation of the coefficient of variation of the fitted concentration values (base 10 logarithm) [1, 4]. The function calls the `invest` function and estimates the fitted concentration given a MFI, the standard error is estimated using the Delta Method. The Coefficient of Variation for the fitted concentration is estimated as:

$$\sqrt{e^{(SE \times ln(10))^2} - 1}$$

where SE is the standard error of the fitted concentration [8].

For a specific coefficient of variation cutoff the LLOQ and HLOQ are calculated as the fitted concentration values whose coefficient of variation is lower or equal to the specified cutoff.

Given a `scluminex` object and a coefficient of variation cutoff (`max.cv` argument), the `loq_cv` function estimates the LOQ. See Figure 7 for a representation of it.

```
> cv <- loq_cv(allanalytes, subset.list="FGF", max.cv=0.2)
> cv
  analyte      lloq     uloq                      method
1     FGF 0.4623362 3.127466 coefficient variation <=0.2
```

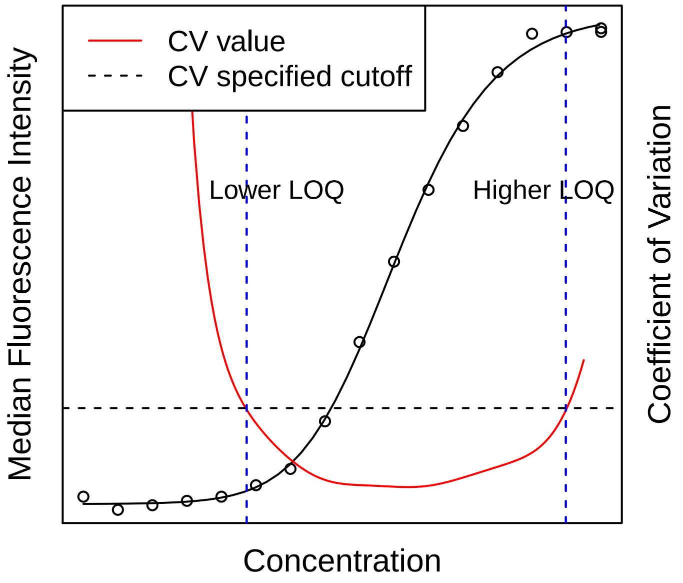

Figure 7: Estimation of limits of quantification based on coefficient of variation method.

The LOQ object is `loq` class:

```
> class(der)
[1] "loqderivatives" "loq"
```

the `summary` method can be applied to obtain more information.

```
> summary(der)
  analyte     lloq     uloq       ly       uy loq.drange y.drange     method
1     FGF 1.088223 2.250466 1.681452 3.097439   1.162243 1.415988 derivative
```

The new variables added are:

- `ly`: the MFI value of the LLOQ (`lloq` variable).
- `uy`: the MFI value of the ULOQ (`uloq` variable).
- `loq.drange`: dynamic range of the LOQ (difference between `uloq` and `lloq`).
- `y.drange`: dynamic range of the MFI values (difference between `uy` and `ly`).

5.4 Fitted concentration

Given a MFI value (base 10 logarithm), the function `invest` estimates the concentration value and the standard error calculating the invert value [11]. The package calculates the inverse functions for the 5-parameters, 4-parameters and exponential functions (in base 10 logarithm) therefore an analytically solution is given. The arguments of the function are:

```
> args(invest)
function (x, analyte = NULL, yvalue, ci.method = c("delta", "bootstrap"),
    level = 0.95, seed.boot = 123, nboot = 100)
NULL
```

Two methods for estimating the confidence interval are available:

Bootstrap: generates `nboot` response vectors (assuming normality) and fit the same inital model with the original concentration data. The confidence interval is calculated by the percentile method specified in the `level` argument. No standard error is estimated.

```
> invesboot <- invest(ig, "FGF", yvalue = 1.4, ci.method="bootstrap")
> invesboot
  log10_mfi log10_concentration.fit log10_concentration.lci
1       1.4               0.4949141               0.3766864
  log10_concentration.uci log10_concentration.se ci.method analyte
1               0.5770771                     NA bootstrap     FGF
```

Delta method: estimates the standard error based on the Delta Method. The function used is `deltamethod` from the `msm` [5] package.

```
> invesdelta <- invest(ig, "FGF", yvalue = 1.4, ci.method="delta")
> invesdelta
  log10_mfi log10_concentration.fit log10_concentration.lci
1       1.4               0.4949141                0.383485
  log10_concentration.uci log10_concentration.se ci.method analyte
1               0.6063431             0.05157872     delta     FGF
```

Also is possible to take into account the dilution of the concentration calling the `est_conc` function. This function is a wrapper of the `invest` one but is specific for concentration estimation. Given a `scluminex` object and a dataset, the function estimates the concentration for each analyte with the corresponding estimated model.

As an example, to select the positive control dataset of the FGF analyte:

```
> concdf <- subset(datasets$plate_1$positive, analyte=="FGF")
```

Assuming dilution factor 1 (same results as `invest` function):

```
> est_conc(ig, concdf, fmfi="mfi", dilution=1)
    sample analyte   plate   well    mfi        ec warning log10.fitted.conc
1 Control1     FGF plate_1  P1_B1 2902.0 1150.0000                 2.7670472
2 Control1     FGF plate_1 P1_G10 3173.5 1150.0000                 2.8704865
3 Control2     FGF plate_1  P1_B2  440.0  143.7500                 1.8702911
4 Control2     FGF plate_1 P1_G11  435.0  143.7500                 1.8667074
5 Control3     FGF plate_1  P1_B3   40.0   21.2963                 0.9635245
6 Control3     FGF plate_1 P1_G12   36.0   21.2963                 0.8886454
  log10.fitted.conc.se dilution dil.fitted.conc dil.lb.conc dil.ub.conc
1           0.03614467        1      584.853627  488.608904  700.056348
2           0.04404870        1      742.141174  596.108199  923.948911
3           0.01475733        1       74.180727   68.930244   79.831144
4           0.01472403        1       73.571118   68.375109   79.161987
5           0.02747050        1        9.194423    8.020068   10.540734
6           0.02966791        1        7.738296    6.676544    8.968896
```

Assuming dilution factor 2:

```
> est_conc(ig, concdf, fmfi="mfi", dilution=2)
    sample analyte   plate   well    mfi        ec warning log10.fitted.conc
1 Control1     FGF plate_1  P1_B1 2902.0 1150.0000                 2.7670472
2 Control1     FGF plate_1 P1_G10 3173.5 1150.0000                 2.8704865
3 Control2     FGF plate_1  P1_B2  440.0  143.7500                 1.8702911
4 Control2     FGF plate_1 P1_G11  435.0  143.7500                 1.8667074
5 Control3     FGF plate_1  P1_B3   40.0   21.2963                 0.9635245
6 Control3     FGF plate_1 P1_G12   36.0   21.2963                 0.8886454
  log10.fitted.conc.se dilution dil.fitted.conc dil.lb.conc dil.ub.conc
1           0.03614467        2      1169.70725   977.21781  1400.11270
2           0.04404870        2      1484.28235  1192.21640  1847.89782
3           0.01475733        2       148.36145   137.86049   159.66229
4           0.01472403        2       147.14224   136.75022   158.32397
5           0.02747050        2        18.38885    16.04014    21.08147
6           0.02966791        2        15.47659    13.35309    17.93779
```

5.5 Agreement between controls

The function `intra_icc` estimates the intraclass correlation coefficient for a dataset in long format. The function calls the `icc` function from the `irr` [3] package.

Taking as example the concentration data for the positive controls:

```
> conc_icc_df <- est_conc(allanalytes, datasets$plate_1$positive,
+     fmfi="mfi", dilution=1)
```

The data has the following structure (6 first rows):

```
> head(conc_icc_df)
    sample analyte   plate   well    mfi        ec warning log10.fitted.conc
1 Control1     FGF plate_1  P1_B1 2902.0 1150.0000                 2.8017672
2 Control1     FGF plate_1 P1_G10 3173.5 1150.0000                 2.9039833
3 Control2     FGF plate_1  P1_B2  440.0  143.7500                 1.8590243
4 Control2     FGF plate_1 P1_G11  435.0  143.7500                 1.8553082
5 Control3     FGF plate_1  P1_B3   40.0   21.2963                 0.9894147
6 Control3     FGF plate_1 P1_G12   36.0   21.2963                 0.9231057
  log10.fitted.conc.se dilution dil.fitted.conc dil.lb.conc dil.ub.conc
1           0.03951173        1      633.529984  519.611216  772.424129
2           0.04442805        1      801.647207  641.479648 1001.806132
3           0.01525222        1       72.281029   66.956476   78.029004
4           0.01525136        1       71.665174   66.386276   77.363839
5           0.02835275        1        9.759211    8.465241   11.250972
6           0.03269666        1        8.377331    7.109937    9.870645
```

To estimate the ICC:

```
> icc_positive <- intra_icc(conc_icc_df, id.var=c("sample", "analyte", "plate"),
+     value.var="dil.fitted.conc", type="agreement",model="twoway",
+     unit="single")
```

where

- `id.var`: indetifies the replicates samples
- `value.var`: the variable to be analyzed.
- others: arguments to be passed to the `icc` function from the `irr` package

There are three objects in the list generated:

Re-Structured dataset

```
> head(icc_positive$icc.df)
  dil.fitted.conc_1 dil.fitted.conc_2   sample analyte   plate
1        633.529984        801.647207 Control1     FGF plate_1
2         72.281029         71.665174 Control2     FGF plate_1
3          9.759211          8.377331 Control3     FGF plate_1
4       1209.316230       1272.055684 Control1    IL1B plate_1
5        167.309524        165.716522 Control2    IL1B plate_1
6         19.680236         21.636014 Control3    IL1B plate_1
```

The ICC object from the `irr` package

```
> names(icc_positive$icc.mod)
 [1] "subjects"   "raters"     "model"      "type"       "unit"
 [6] "icc.name"   "value"      "r0"         "Fvalue"     "df1"
[11] "df2"        "p.value"    "conf.level" "lbound"     "ubound"
```

The ICC estimation

```
> icc_positive$icc.value
[1] 0.9871378
```

6 Summary of results

The `scluminex` object can be printed, summarized and plotted.

Print: the name of the analytes is listed.

```
> allanalytes
 [1] "FGF"     "IL1B"    "G-CSF"   "IL10"    "IL13"    "IL6"     "IL12"
 [8] "RANTES"  "EOTAXIN" "IL17"    "MIP1A"   "GMCSF"   "MIP1B"   "MCP1"
[15] "IL15"    "EGF"     "IL5"     "HGF"     "VEGF"    "IFNg"    "IFNa"
[22] "IL1RA"   "TNFa"    "IL2"     "IL7"     "IP10"    "IL2R"    "MIG"
[29] "IL4"     "IL8"
```

Summary: a dataset is generated showing the estimated coefficients, number of observations, R^2, convergence and the fitted function for each analyte. To extract more information `as.data.frame` can be applied to a `summary.scluminex` object or to a `scluminex`. The methodology applied:

Neill test: is an ANOVA-based lack-of-fit test [6]. The method does not require replicates for concentration values but assumes that predictor variable can be grouped. The function is an adaptation of the `neill.test` from `drc` package [10]. The p-value of the test is reported.

$\bar{R}^2$**:** is the adjusted version of R^2 which takes into account the number of fitted parameters, estimated as:

$$\left(1 - \frac{\frac{SS_{err}}{(n-p-1)}}{\frac{SS_{tot}}{(n-1)}}\right)$$

where,

- n: number of observations
- p: number of estimated coefficients
- SS_{err}: residual sum of squares
- SS_{tot}: total sum of squares

AIC: Akaike information criterion estimated as:

$$(-2 \times (log - likelihood) + 2 \times p)$$

where p is the number of estimated parameters in the model. The function applied to the fitted model is the generic `AIC` function for `nls` class object.

As example, the `summary` method applied to a `scluminex` object

```
> summary(allanalytes)
   analyte          b          c        d         e         f obs   rsquare convergence  fct
1      FGF -0.8828771  1.3068483 3.683054 1.3890770 1.7678441  17 0.9986956 convergence SS15
2     IL1B -0.7291501  1.2823197 4.157377 2.2406824 1.1845345  17 0.9997494 convergence SS15
3    G-CSF -0.7229726  0.7586540 4.092740 2.6835136 1.2948285  17 0.9994358 convergence SS15
4     IL10 -0.4523784  0.6016439 4.595441 1.9790133 0.9125607  17 0.9994614 convergence SS15
5     IL13 -0.8043012  0.8938679 4.283454 1.9005612 1.6165574  17 0.9996548 convergence SS15
6      IL6 -0.6209108  0.9814027 4.348758 1.9220673 0.7637184  17 0.9992782 convergence SS15
7     IL12 -0.6862771  0.8229139 4.204463 2.2984784 1.3830266  17 0.9998644 convergence SS15
8   RANTES -0.9437332  1.7723069 3.918469 1.6347323 1.7387868  17 0.9994649 convergence SS15
9  EOTAXIN -0.5210368  1.4316138 4.289606 0.8253446 2.1946897  17 0.9997680 convergence SS15
10    IL17 -0.7770365  0.8729686 4.437082 2.6606798 0.5119282  17 0.9996918 convergence SS15
11   MIP1A -0.7290696  0.9101793 3.990784 1.5526541 1.9647135  17 0.9998835 convergence SS15
12   GMCSF -0.4091215  0.3682323 4.531576 1.2278351        NA  17 0.9991905 convergence SS14
13   MIP1B -0.5502088  1.0419523 4.381991 2.0851423 1.5433827  17 0.9997014 convergence SS15
14    MCP1 -0.6368871  1.0210596 4.558936 2.2773092 1.4159834  17 0.9998101 convergence SS15
15    IL15 -1.2741555  1.3014988 3.801743 3.2553293 0.4213175  17 0.9989005 convergence SS15
16     EGF -0.7775902  1.4265917 4.247292 1.9743882 0.7942679  17 0.9999056 convergence SS15
17     IL5 -0.7358589 -0.1865238 4.482711 2.5280240 0.2742499  17 0.9994644 convergence SS15
18     HGF -0.4779733  0.8688056 5.138004 2.5832461 2.0644387  17 0.9996647 convergence SS15
19    VEGF -0.8248516  1.5726924 3.950033 1.6801506 1.0039745  17 0.9997100 convergence SS15
20    IFNg -0.4707418  0.6146812 4.754997 2.3781928        NA  17 0.9992237 convergence SS14
21    IFNa -0.9684408  0.7668357 4.234176 2.2819768 0.9519239  17 0.9993487 convergence SS15
22   IL1RA -0.6535843  1.6186756 3.973113 3.1939268        NA  17 0.9977053 convergence SS14
23    TNFa -0.3705635  0.7370135 4.773413 0.9077849 2.1495834  17 0.9994428 convergence SS15
24     IL2 -0.6672199  0.9780458 4.410199 2.3856437 0.7218382  17 0.9998813 convergence SS15
25     IL7 -0.8784672  1.5961995 4.267243 2.4553340 1.1748884  17 0.9997071 convergence SS15
26    IP10 -0.7080650  0.7865411 4.353835 0.9641264 1.5094283  17 0.9996376 convergence SS15
27    IL2R -0.5859247  0.9110713 4.285251 2.7511816 1.2448516  17 0.9996893 convergence SS15
28     MIG -0.6499398  0.8284273 4.598374 1.9951056 1.8461333  17 0.9996634 convergence SS15
29     IL4 -0.5490025  0.8743643 4.339536 2.1432739 1.3927581  17 0.9998933 convergence SS15
30     IL8 -0.6469705  1.7275331 4.135944 2.0599591 1.4727478  17 0.9996691 convergence SS15
```

the `as.data.frame` method to a `scluminex` object

```
> as.data.frame(ig)
   analyte    mfi           ec   well log10_mfi log10_concentration
1      FGF 4096.0 3450.0000000  P1_A2  3.612360          3.53781910
2      FGF 3933.0 3450.0000000  P1_H4  3.594724          3.53781910
3      FGF 3925.0 1725.0000000  P1_A3  3.593840          3.23678910
4      FGF 2510.0  431.2500000  P1_A4  3.399674          2.63472911
5      FGF  675.0  107.8125000  P1_A5  2.829304          2.03266912
6      FGF 3854.0  862.5000000  P1_H5  3.585912          2.93575910
7      FGF  123.5   26.9531250  P1_A6  2.091667          1.43060913
8      FGF 1377.0  215.6250000  P1_H6  3.138934          2.33369911
9      FGF   30.0    6.7382812  P1_A7  1.477121          0.82854913
10     FGF  303.0   53.9062500  P1_H7  2.481443          1.73163912
11     FGF   22.0    1.6845703  P1_A8  1.342423          0.22648914
12     FGF   51.0   13.4765625  P1_H8  1.707570          1.12957913
13     FGF   20.0    0.4211426  P1_A9  1.301030         -0.37557085
14     FGF   25.0    3.3691406  P1_H9  1.397940          0.52751914
15     FGF   22.0    0.1052856 P1_A10  1.342423         -0.97763084
16     FGF   21.0    0.8422852 P1_H10  1.322219         -0.07454085
17     FGF   19.0    0.2105713 P1_H11  1.278754         -0.67660084
                      warning predicted.log10_mfi    residuals
1                                        3.619779 -0.204908310
2                                        3.619779 -0.691997531
3                                        3.586877  0.192313387
4                                        3.399745 -0.001967785
5                                        2.859326 -0.829174292
6                                        3.522083  1.762897543
7                                        2.042835  1.348683934
8                                        3.186101 -1.302704181
9                                        1.523880 -1.291419811
10                                       2.448572  0.907867003
11                                       1.347986 -0.153657570
12                                       1.727064 -0.538396036
13                                       1.302009 -0.027032456
14                                       1.408672 -0.296396611
15                                       1.290855  1.424237036
16                                       1.317258  0.137038697
17 Not_Estimated_Concentration           1.294517 -0.435383042
   log10.fitted.conc log10.fitted.conc.se plateid
1        3.449503479           0.20302133 plate_1
2        3.292007536           0.13140535 plate_1
3        3.285427496           0.12902475 plate_1
4        2.634596941           0.02984423 plate_1
5        2.009076031           0.01649710 plate_1
6        3.230453096           0.11075190 plate_1
7        1.469657477           0.01615612 plate_1
8        2.282880986           0.02131608 plate_1
9        0.727426969           0.03567570 plate_1
10       1.754876098           0.01406998 plate_1
11       0.185252694           0.10496266 plate_1
12       1.106420774           0.02375991 plate_1
13      -0.404669644           0.48753881 plate_1
14       0.486818796           0.05239479 plate_1
15       0.185252694           0.10496266 plate_1
16      -0.009466399           0.17418522 plate_1
17               NaN                  NaN plate_1
```

and `as.data.frame` to a `summary.scluminex` method:

```
> ss <- summary(ig)
> as.data.frame(ss)
  analyte         b        c        d        e       b_se       c_se       d_se
1     FGF -1.039396 1.287375 3.651836 1.746509 0.04389603 0.01758703 0.02389215
        e_se       b_t      c_t      d_t      e_t       b_pval       c_pval
1 0.01906806 -23.67858 73.20025 152.8467 91.59341 4.469256e-12 2.148862e-18
       d_pval       e_pval obs   rsquare modelfit       aic convergence plateid
1 1.51517e-22 1.172038e-19  17 0.9985489    0.299 -59.14575 convergence plate_1
   fct bkg_mean bkg_method
1 SSl4 19.97498     ignore
```

Plot: standard curves, standardized residuals or Q-Q plot of the residuals are plotted. The function is based on `ggplot2` so other data can be added to the plot. The plot is specified by the `type` argument.

The default type is `scurve` and the function allows to plot the standard curve of a plate for all analytes or just the ones desired. Also allows to plot some other aspects as legend, confidence bands, background or specify the number of columns.

```
> plot(allanalytes, type = "scurve", ncol=5, psize=1)
```

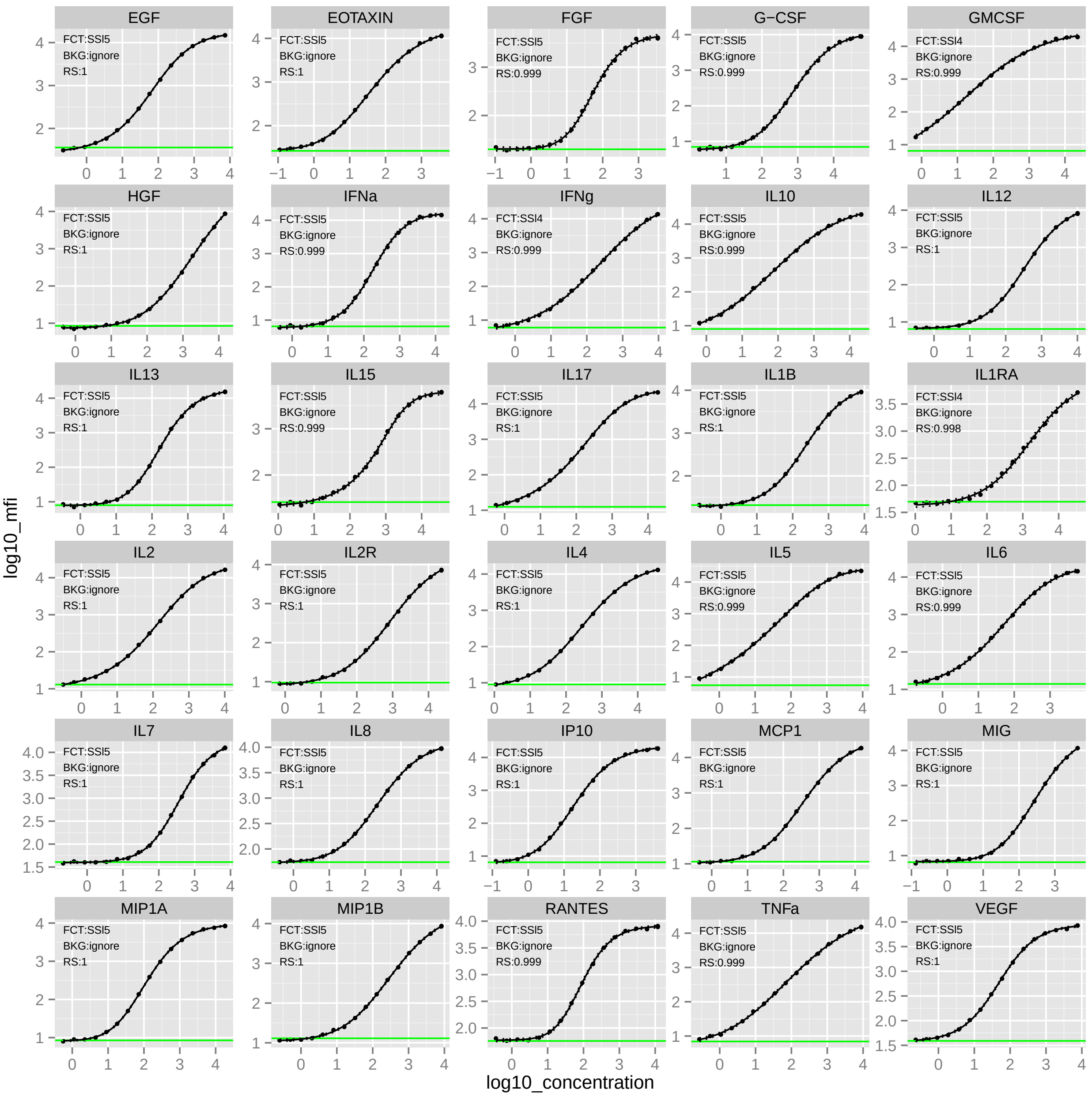

The type **residuals** show the standardized residuals. The points beyond the **out.limit** argument are presented in red and the well variable is shown.

```
> plot(allanalytes, type = "residuals", out.limit= 2.5, ncol=5, psize=1)
```

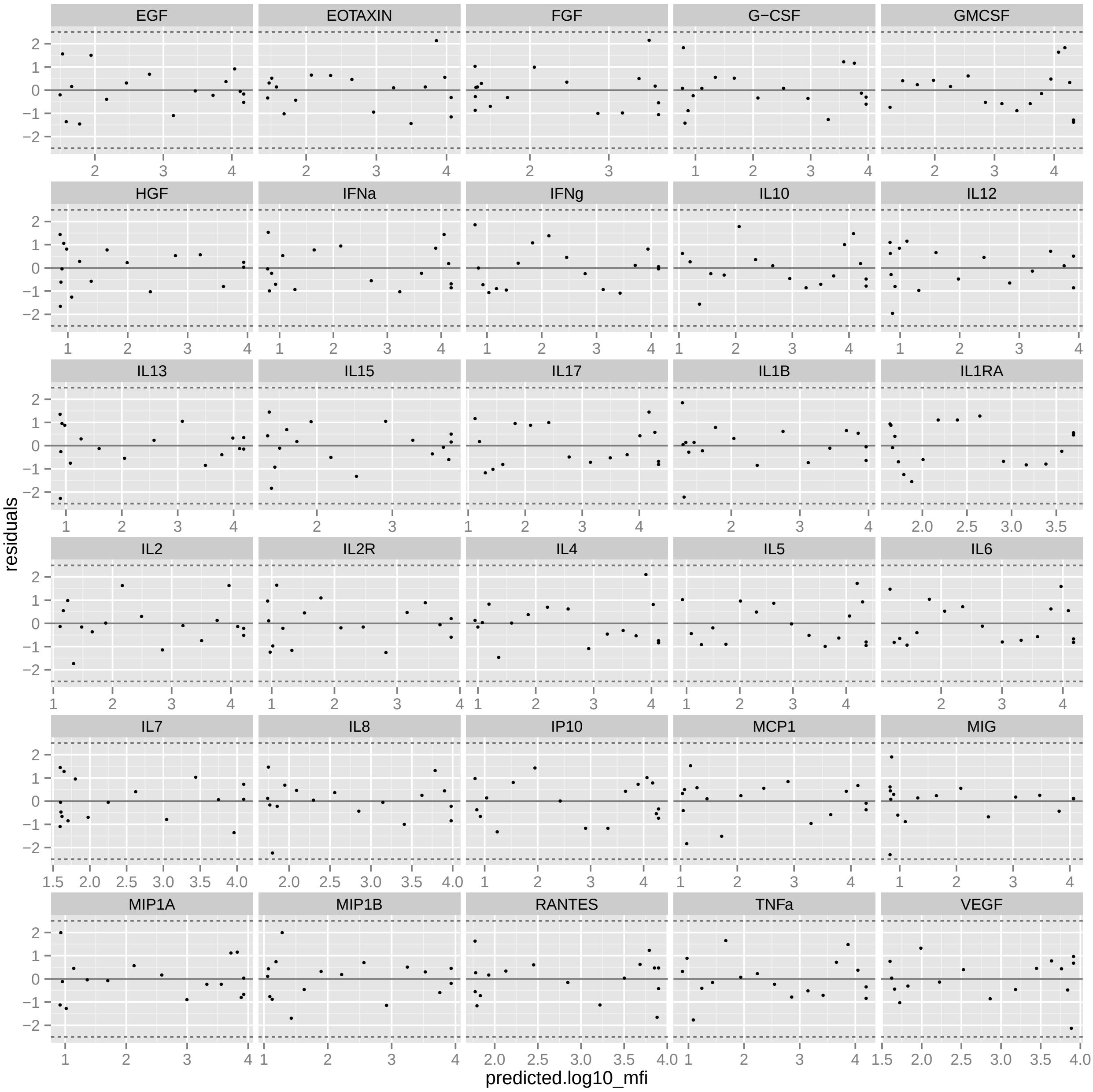

The type qqplot generates the Q-Q plot of the standardized residuals.

```
> plot(allanalytes,  type = "qqplot", ncol=5, psize=1)
```

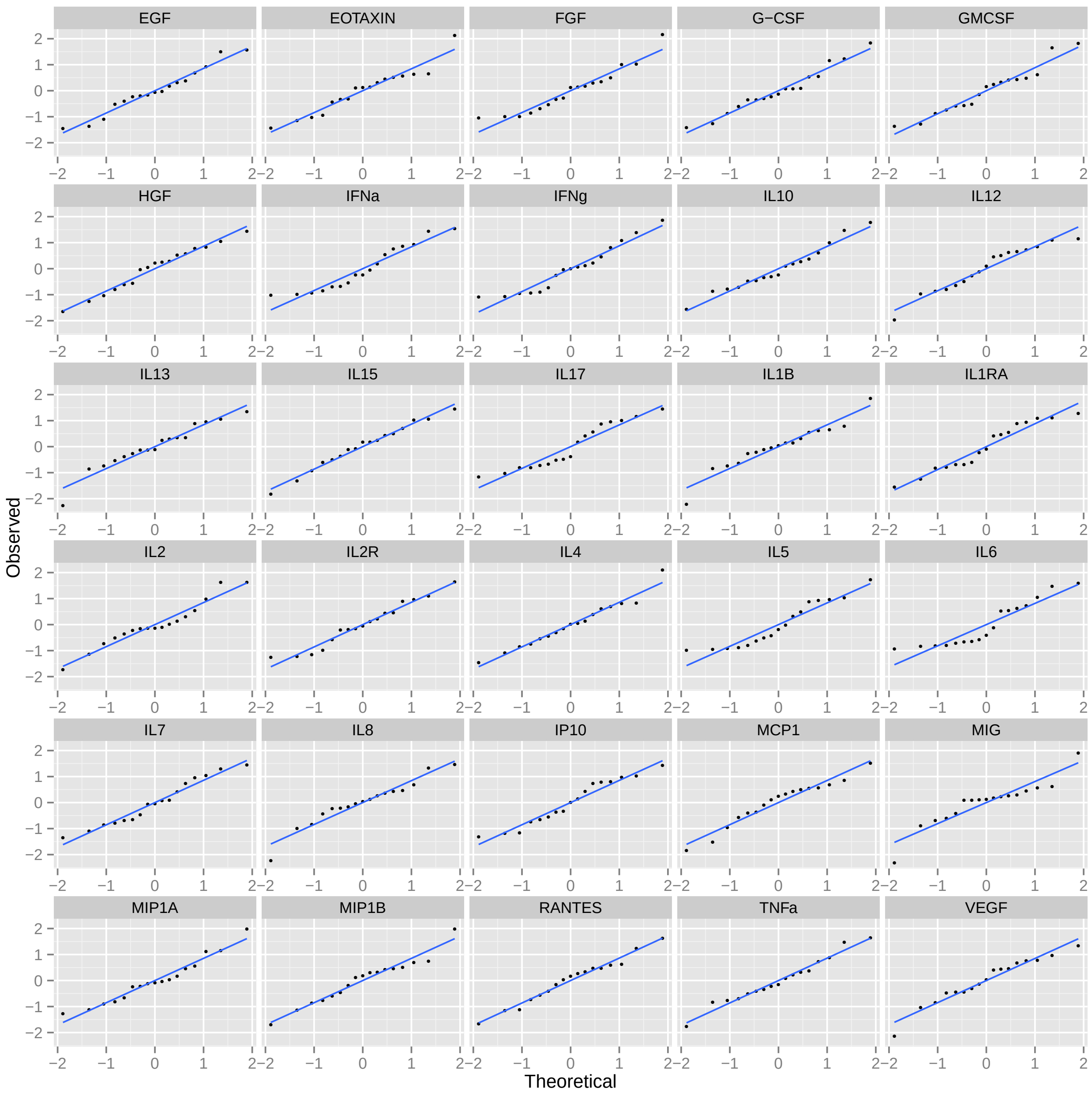

7 Flag data

The package implements several features to easily identify outliers. The function get_outliers allows to identify the points of the standard curve with a standardized residual greater than a specified value.

```
> get_outliers(allanalytes, out.limit=2)
        batch_well_analyte    batch   well analyte    flag observations
6       newplate*P1_H5*FGF newplate  P1_H5     FGF OUTLIER     2.153402
30     newplate*P1_A9*IL1B newplate  P1_A9    IL1B OUTLIER    -2.220823
85    newplate*P1_H11*IL13 newplate P1_H11    IL13 OUTLIER    -2.266228
142 newplate*P1_H5*EOTAXIN newplate  P1_H5 EOTAXIN OUTLIER     2.127560
```

```
309    newplate*P1_A3*VEGF newplate  P1_A3    VEGF OUTLIER    -2.136298
474    newplate*P1_A10*MIG newplate P1_A10     MIG OUTLIER    -2.313133
482     newplate*P1_H5*IL4 newplate  P1_H5     IL4 OUTLIER     2.099858
509    newplate*P1_H10*IL8 newplate P1_H10     IL8 OUTLIER    -2.236779
```

As commented previously the plot of the residuals also allows to identify the same points

```
> plot(allanalytes, "residuals", out.limit=2, size.text=2.5)
```

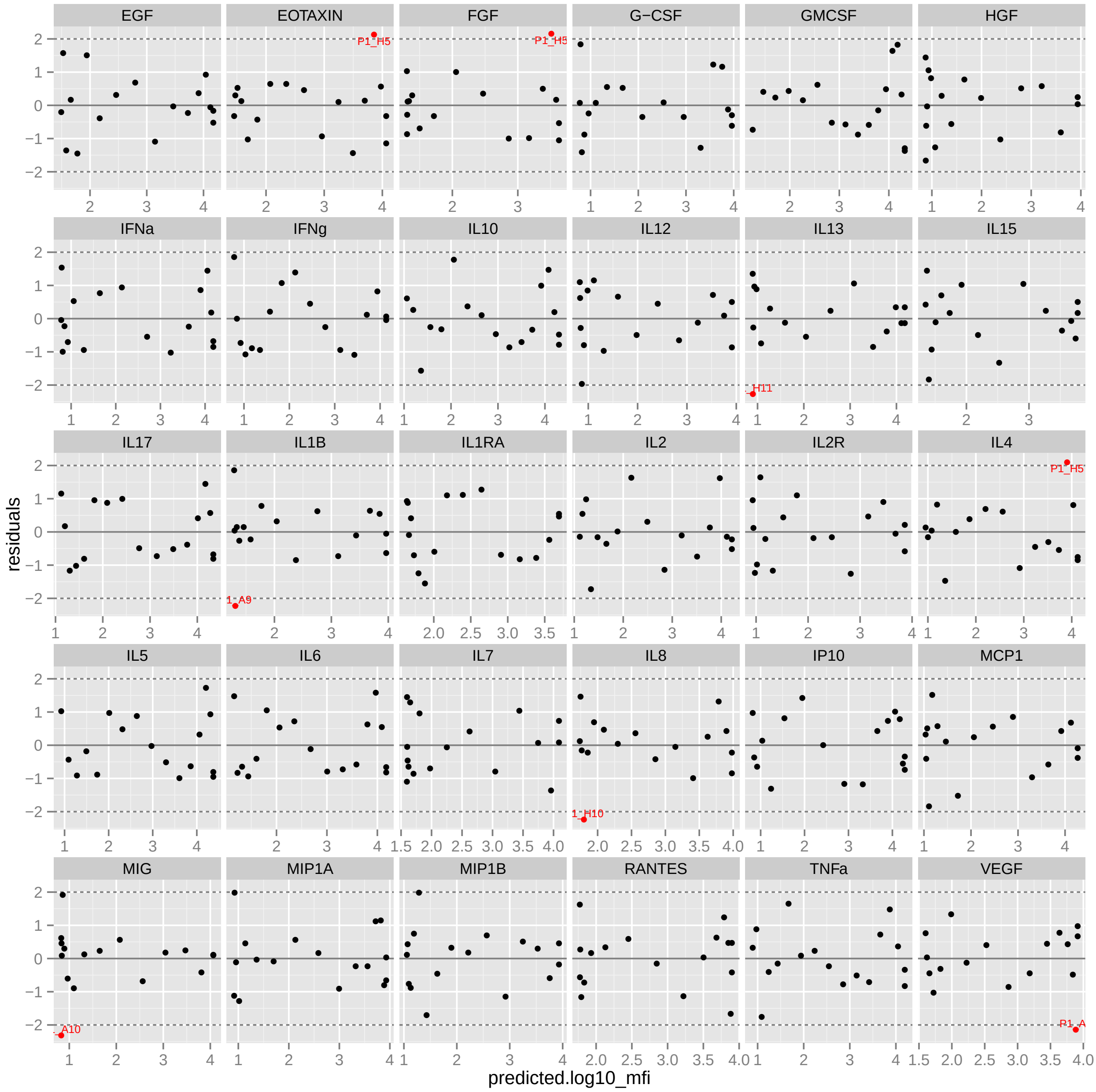

Once we have identified the outliers we can add this information to the data, using `data_selection` or the `merge` function.

```
> out <- get_outliers(allanalytes, out.limit=2)
> flag.dat <- merge(datasets$plate_1$standard, out, by=c("analyte","well"),all.x=TRUE)
```

After we can run the `scluminex` function.

```
> flag.allanalytes <- scluminex(plateid = "newplate.flag",
+     standard = flag.dat,
+     background = datasets$plate_1$background,
+     bkg = "ignore", lfct = c("SSl5","SSl4"),
+     fmfi = "mfi", verbose = FALSE)
```

And plot the standard curve. The flagged points are shown as non-filled points but they are not included in the estimation of the curve.

```
> plot(flag.allanalytes, "scurve",
+      subset.list=c("FGF","IL1B", "IL13","EOTAXIN","VEGF","MIG","IL4","IL8"),
+      ncol=4, psize=2, size.legend=3)
```

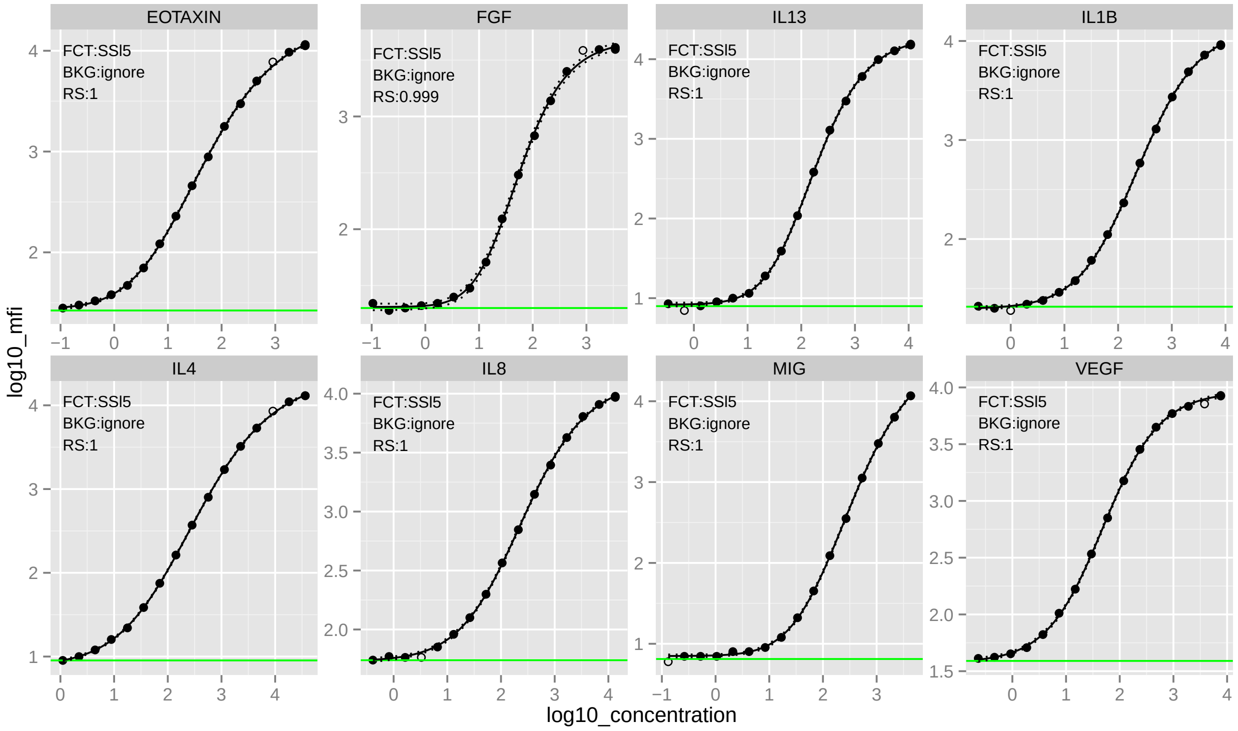

8 Raw data from xPONENT® software

The package allows to import CSV raw data that has been exported from xPONENT® software. The `lum_import` function identifies sections of information from this data. Moreover the function imports Bead raw data.

8.1 MFI raw data

There is an example of MFI raw data included in the package. The CSV file has several blocks of information that need to be extracted and restructured in order to analyze it. This raw data can be imported calling the `lum_import` function:

```
> imp_path <-  system.file(c("inst","extdata"),"plate1.csv", package="drLumi")
> imp <- lum_import(imp_path)
> imp
The file imported is: Fluorescence type
```

```
Identified data type: Median
Identified data type: Net MFI
Identified data type: Count
Identified data type: Result
Identified data type: Range
Identified data type: Avg Net MFI
Identified data type: Avg Result
Identified data type: Avg Range
Identified data type: %CV Replicates
Identified data type: % Recovery
Identified data type: Comments
Identified data type: Units
Identified data type: Standard Expected Concentration
Identified data type: Control Expected Concentration
Identified data type: Control Range - Low
Identified data type: Control Range - High
Identified data type: Per Bead Count
Identified data type: Dilution Factor
Identified data type: Analysis Types
Identified data type: Analysis Coefficients
Identified data type: R^2
Identified data type: Audit Logs
Identified data type: Warnings/Errors
```

The function identifies the section parts of the CSV file and groups the data in several datasets. The `lum_import` object has the following objects:

- `dtblock`: blocks of information from original CSV file.
- `raw_metadata`: a `data.frame` with the information of batch (software version, operator, batch date ...).
- `vars` type object: variables that are going to be exported in the `lum_export` function. These variables can be modified in order to remove or add more.
- `name_batch`: the name of the batch as it is described in raw data.
- `type_raw_data`: Fluorescence (MFI values for samples) or Bead (for Bead data).

After the identification of the raw data is necessary to extract the information from the `lum_import` object. This can be done using the `lum_export` function which generates several datasets based on the identified sections and the specified variables:

```
> expdb <- lum_export(imp)
> expdb
Dataframes:
well scurve average batch region sample name_batch
```

As described previously variables can be removed (or new ones can be added). Following is an example for the selection of 2 variables from the well dataset:

```
> imp$well_vars
[1] "Median"     "Net MFI"    "Count"      "Result"     "Range"
[6] "% Recovery" "Comments"
> imp$well_vars <- c("Median", "Net MFI")
> exp <- lum_export(imp)
```

```
> head(exp$well)
  batch_well_analyte   batch  well analyte        sample median net_mfi
1  plate_1*P1_A1*FGF plate_1 P1_A1     FGF   Background0     21       1
2  plate_1*P1_B1*FGF plate_1 P1_B1     FGF      Control1   2902    2882
3  plate_1*P1_C1*FGF plate_1 P1_C1     FGF  B_sid_13_CSP     18      -2
4  plate_1*P1_D1*FGF plate_1 P1_D1     FGF B_sid_13_DMSO     19      -1
5  plate_1*P1_E1*FGF plate_1 P1_E1     FGF  B_sid_13_HBS     17      -3
6  plate_1*P1_F1*FGF plate_1 P1_F1     FGF B_sid_13_AMA1     18      -2
```

8.2 Bead raw data

Bead raw data has several files (usually one file per well). This type of data can be imported either from a folder or from a zip file. The function assumes that all well files within the folder are in CSV format and try to combine all information. To identify the files new variables are added:

- well: with the name of the CSV file
- batch: the name of the file
- batch_well_eventno: a combination of well, batch and eventno variables.

Reading non-zip compressed data:

```
> imp_path_nozip <- system.file(c("inst","extdata"),"bead_data",
+                               package="drLumi")
> bead_nozip <- lum_import(imp_path_nozip)
> bead_nozip
The file imported is: Bead type
Number of unique wells files 2
```

The code for reading zip files is the same as reading non-compressed files and returns the same information. The only difference is that first unzip the file and creates a new folder with the same name as the original in the same directory where the zip file is located.
All CSV files are combined in one `data.frame`:

```
> head(bead_nozip$bead_files)
  eventno rid dbl    dd rp1 cl1 cl2 aux1 time        well     batch
1       0   0   0 24977  13 117   0    0    0 plate_P1_A1 bead_data
2       1   0   0 25917   0 281  10    0    0 plate_P1_A1 bead_data
3       2   0   0 26555   0 394  17    0    0 plate_P1_A1 bead_data
4       3   0   0 24832   0  68   0    0    0 plate_P1_A1 bead_data
5       4   0   0 28166   0 419  29    0    0 plate_P1_A1 bead_data
6       5   0   0 27034   0 404  29    0    0 plate_P1_A1 bead_data
       batch_well_eventno
1 bead_data*plate_P1_A1*0
2 bead_data*plate_P1_A1*1
3 bead_data*plate_P1_A1*2
4 bead_data*plate_P1_A1*3
5 bead_data*plate_P1_A1*4
6 bead_data*plate_P1_A1*5
```

And the files are identifiable by the well variable:

```
> with(bead_nozip$bead_files, table(well, batch))
             batch
well           bead_data
  plate_P1_A1       4981
  plate_P1_A2       4647
```

References

[1] Defawe, O., Fong, Y., Vasilyeva, E., Pickett, M., Carter, D., Gabriel, E., Rerks-Ngarm, S., Nityaphan, S., Frahm, N., McElrath, M., and Rosa, S. D. Optimization and qualification of a multiplex bead array to assess cytokine and chemokine production by vaccine-specific cells. *J Immunol Methods. 382* (2012), 117–128.

[2] Elzhov, T., Mullen, K. M., Spiess, A.-N., and Bolker, B. *minpack.lm: R interface to the Levenberg-Marquardt nonlinear least-squares algorithm found in MINPACK, plus support for bounds*, 2013. R package version 1.1.8.

[3] Gamer, M., Lemon, J., Fellows, I., and Puspendra, S. *irr: Various Coefficients of Interrater Reliability and Agreement*, 2012. R package version 0.84.

[4] Gottschalk, P., and Dunn, J. Determining the error of dose estimates and minimum and maximum acceptable concentrations from assays with nonlinear dose-response curves. *Comput. Methods Programs Biomed 80* (2005), 204–215.

[5] Jackson, C. *msm: Multi-state Markov and hidden Markov models in continuous time*, 2014. R package version 1.4.

[6] Neill, J. Testing for lack of fit in nonlinear regression. *Ann. Statist. 16* (1988), 733–740.

[7] Quinn, C. P., Semenova, V. A., Elie, C. M., Sandra Romero-Steiner, C. G., Li, H., Stamey, K., Steward-Clark, E., Schmidt, D. S., Mothershed, E., Pruckler, J., Schwartz, S., Benson, R. F., Helsel, L. O., Holder, P. F., Johnson, S. E., Kellum, M., Messmer, T., Thacker, W. L., Besser, L., Plikaytis, B. D., Taylor, T. H., Freeman, A. E., Wallace, K. J., Dull, P., Sejvar, J., Bruce, E., Moreno, R., Schuchat, A., Lingappa, J. R., Martin, S. K., Walls, J., Bronsdon, M., Carlone, G. M., Bajani-Ari, M., Ashford, D. A., Stephens, D. S., and Perkins, B. A. Specific, sensitive, and quantitative enzyme-linked immunosorbent assay for human immunoglobulin g antibodies to anthrax toxin protective antigen. *Emerg Infect Dis 10* (2002), 1103–1110.

[8] Reed, G., Lynn, F., and Meade, B. Use of coefficient of variation in assessing variability of quantitative assays. *Clin Diagn Lab Immunol 9* (2002), 1235–1239.

[9] Ritz, C., and Spiess, A. qpcr: an r package for sigmoidal model selection in quantitative real-time polymerase chain reaction analysis. *Bioinformatics 24* (2008), 1549–1551.

[10] Ritz, C., and Strebig, J. *drc: Analysis of dose-response curve data*, 2013. R package version 2.3-96.

[11] Ruckstuhl, A. *Introduction to Nonlinear Regression*, 2010.
