# Supplementary figures and images for: drLumi: An open-source package to manage data, calibrate, and conduct quality control of multiplex bead-based immunoassays data analysis
Source: PLoS One. 2017 Nov 14;12(11):e0187901. doi: 10.1371/journal.pone.0187901 (PMC5685631; doi:10.1371/journal.pone.0187901)

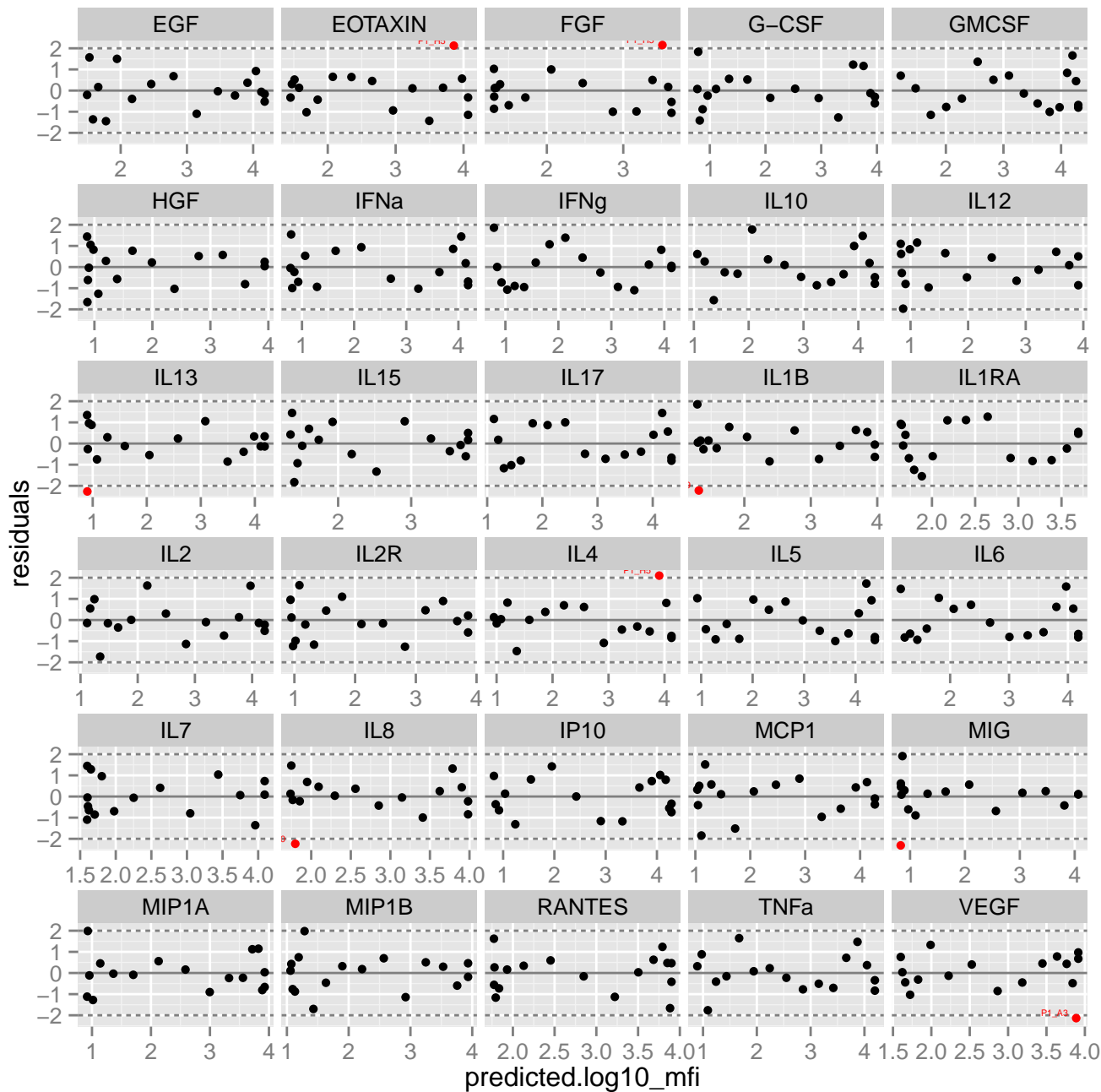

Supplement: S2 File — (TAR) [file pone.0187901.s002.tar › drLumi/vignettes/unnamed-chunk-35.pdf]

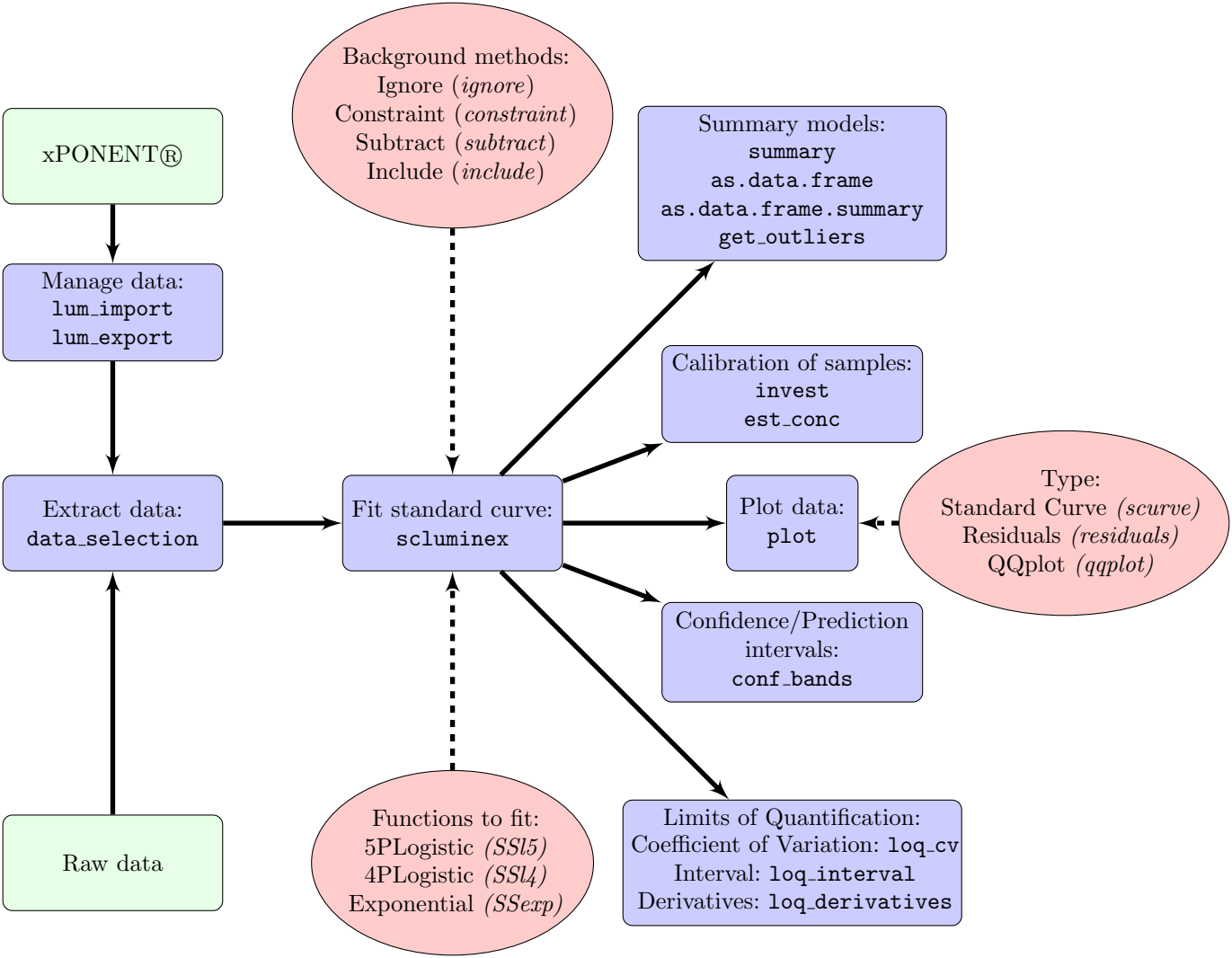

Supplement: S2 File — (TAR) [file pone.0187901.s002.tar › drLumi/vignettes/flow.pdf]

# Exponential growth

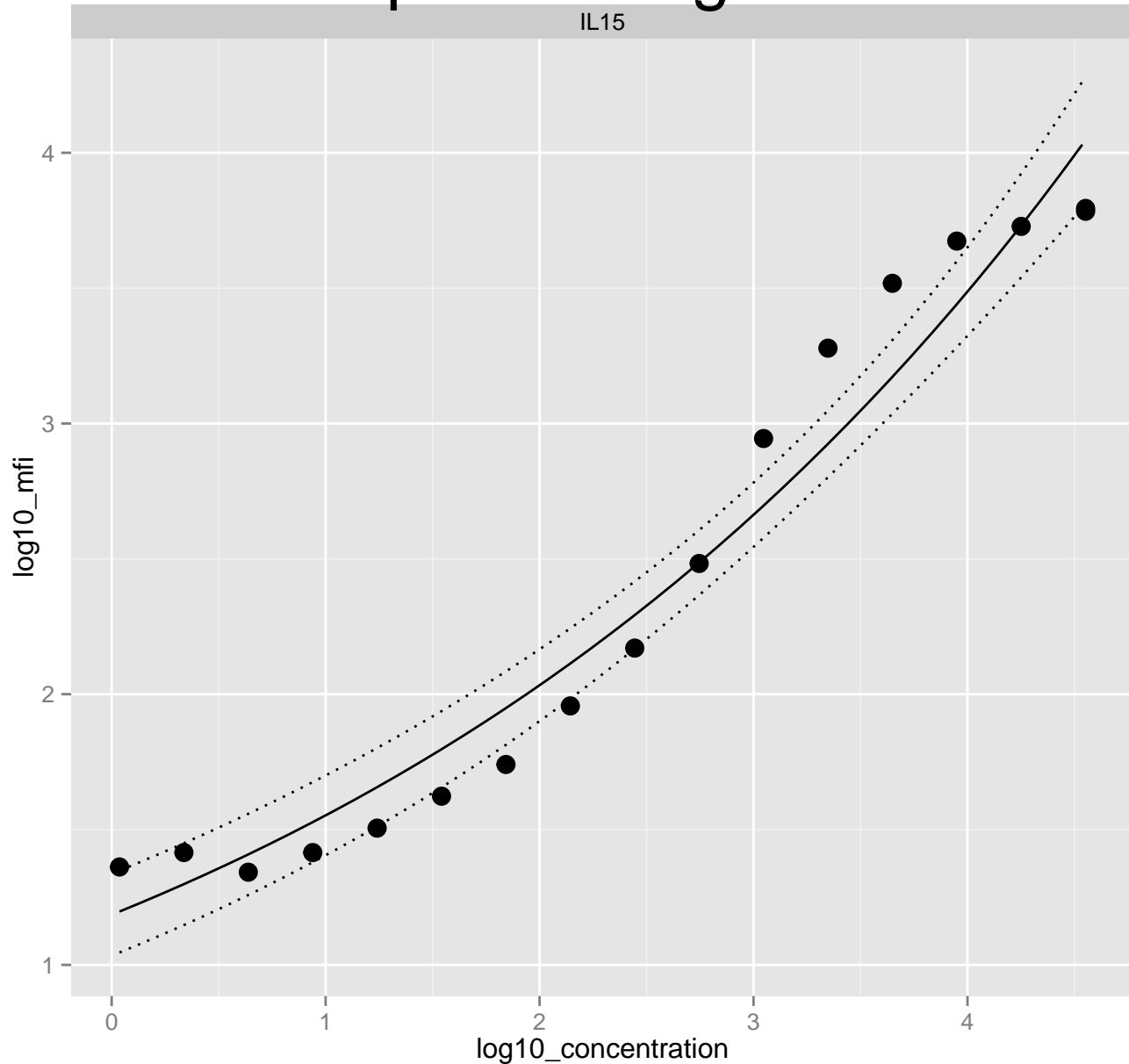

Supplement: S2 File — (TAR) [file pone.0187901.s002.tar › drLumi/vignettes/unnamed-chunk-19.pdf]

$y + 2$

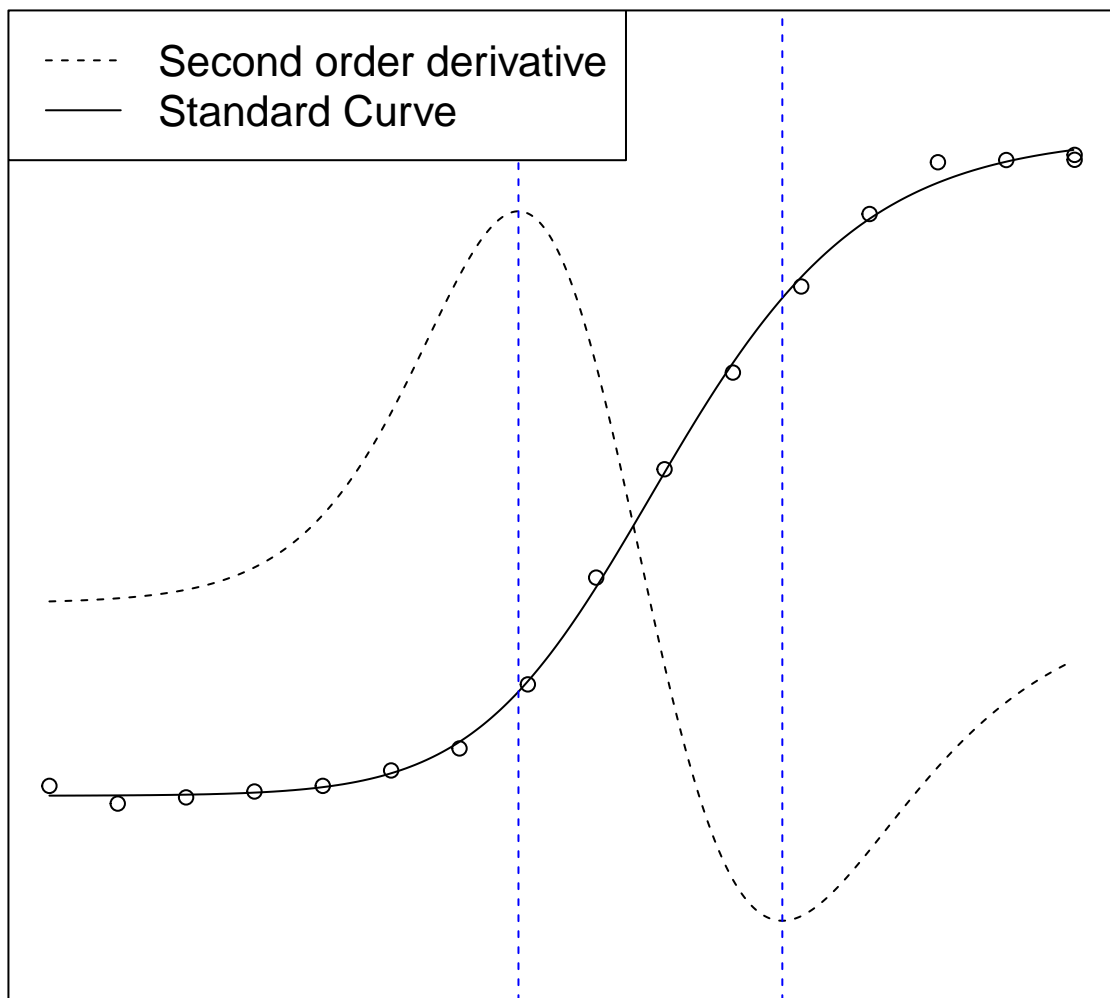

suport

Supplement: S2 File — (TAR) [file pone.0187901.s002.tar › drLumi/vignettes/unnamed-chunk-26-1.pdf]

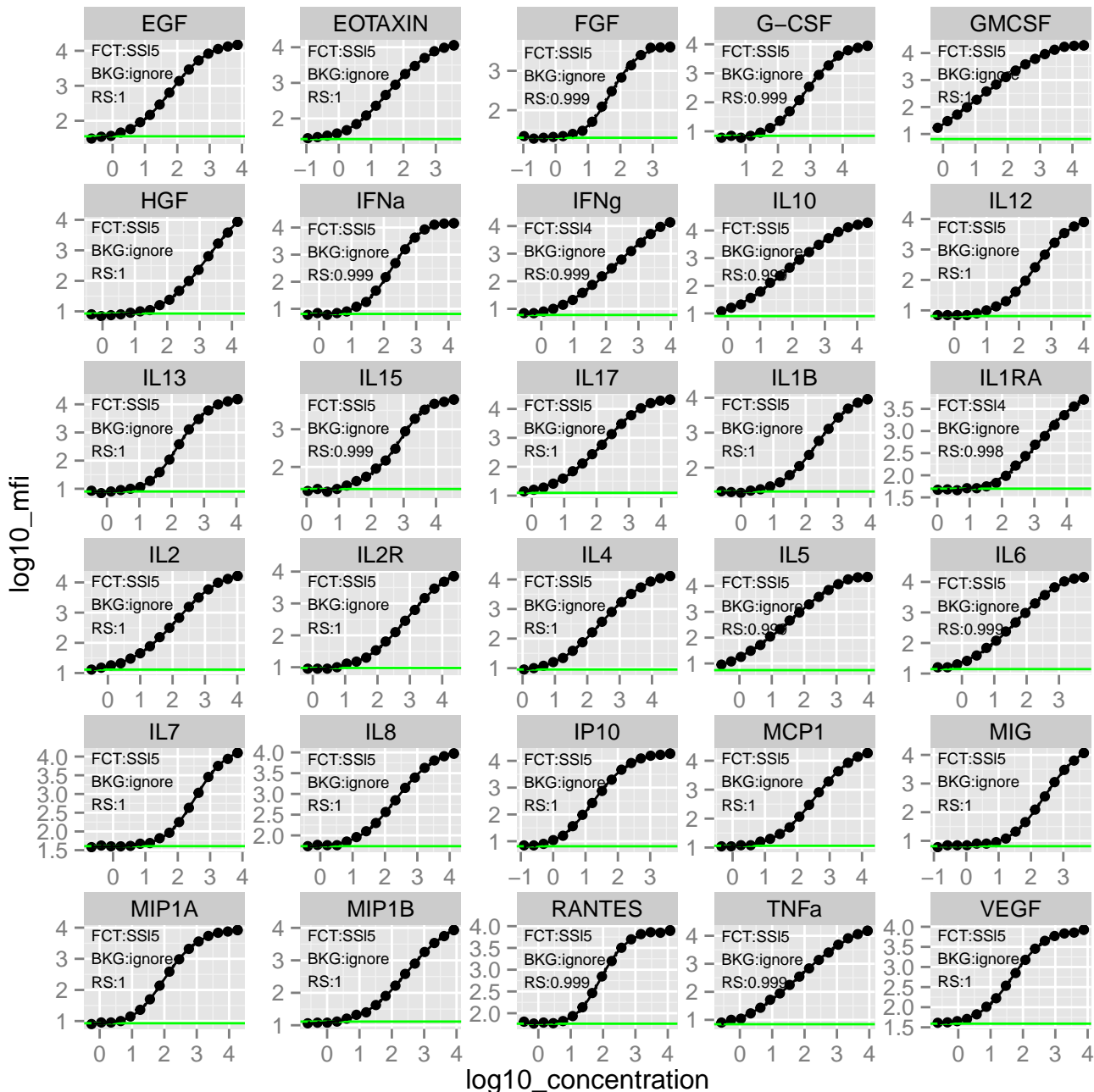

Supplement: S2 File — (TAR) [file pone.0187901.s002.tar › drLumi/vignettes/unnamed-chunk-34.pdf]

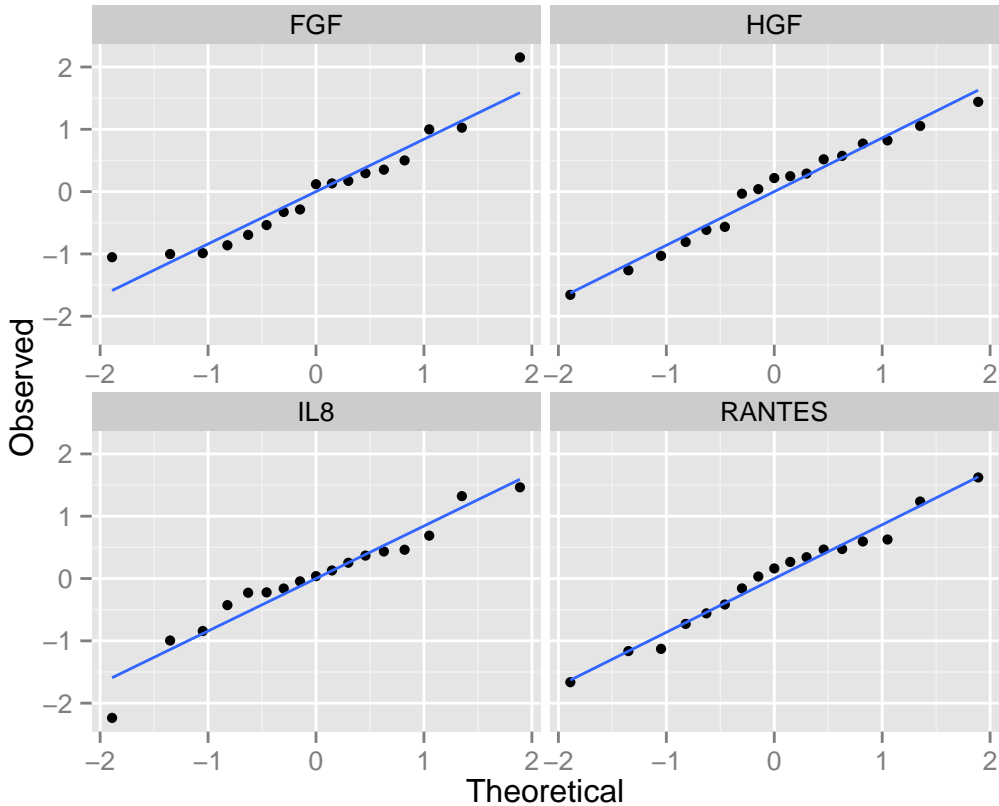

Supplement: S2 File — (TAR) [file pone.0187901.s002.tar › drLumi/vignettes/qqplot.pdf]

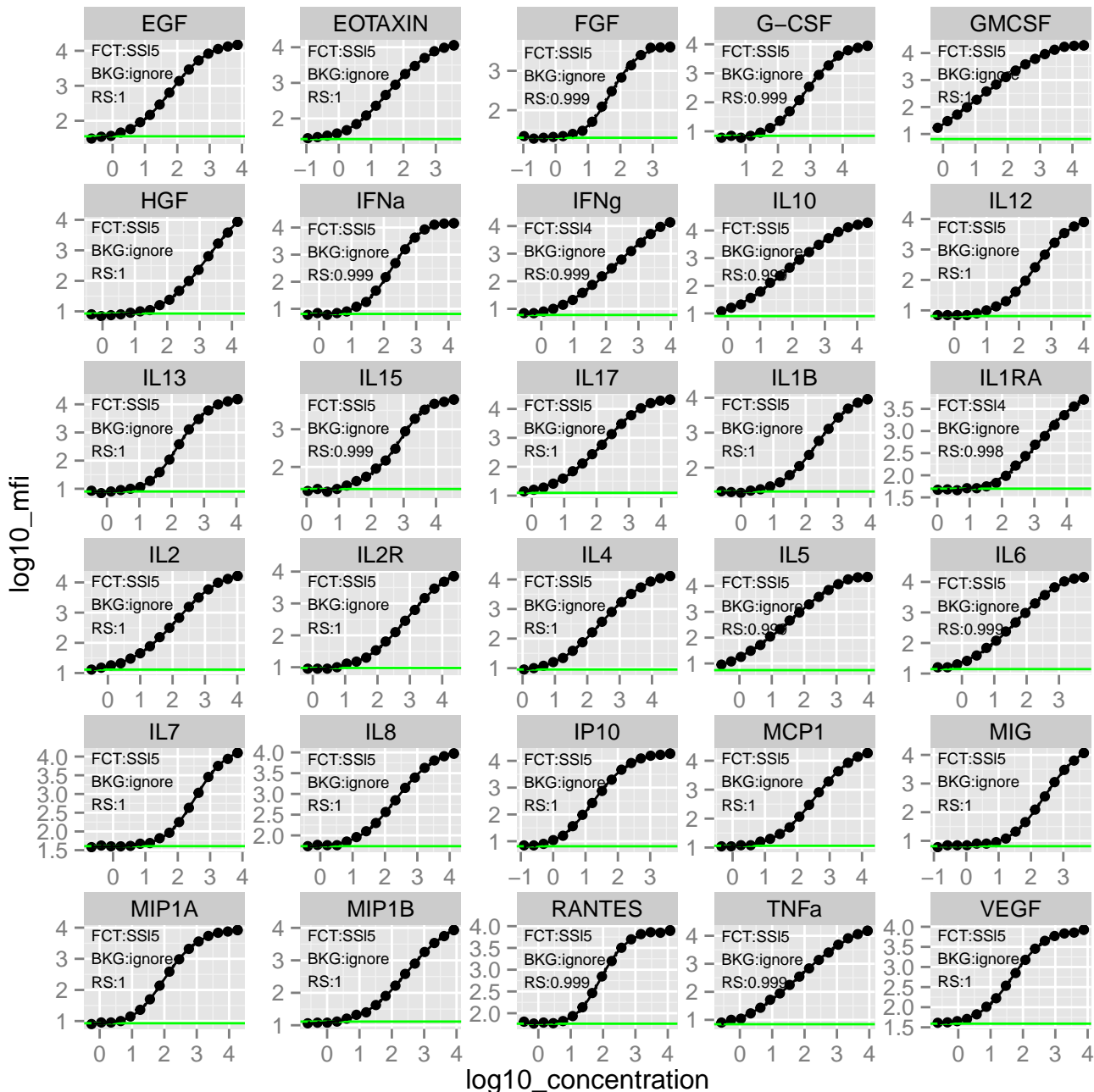

Supplement: S2 File — (TAR) [file pone.0187901.s002.tar › drLumi/vignettes/unnamed-chunk-36.pdf]

- - - Second order derivative  
— Standard Curve

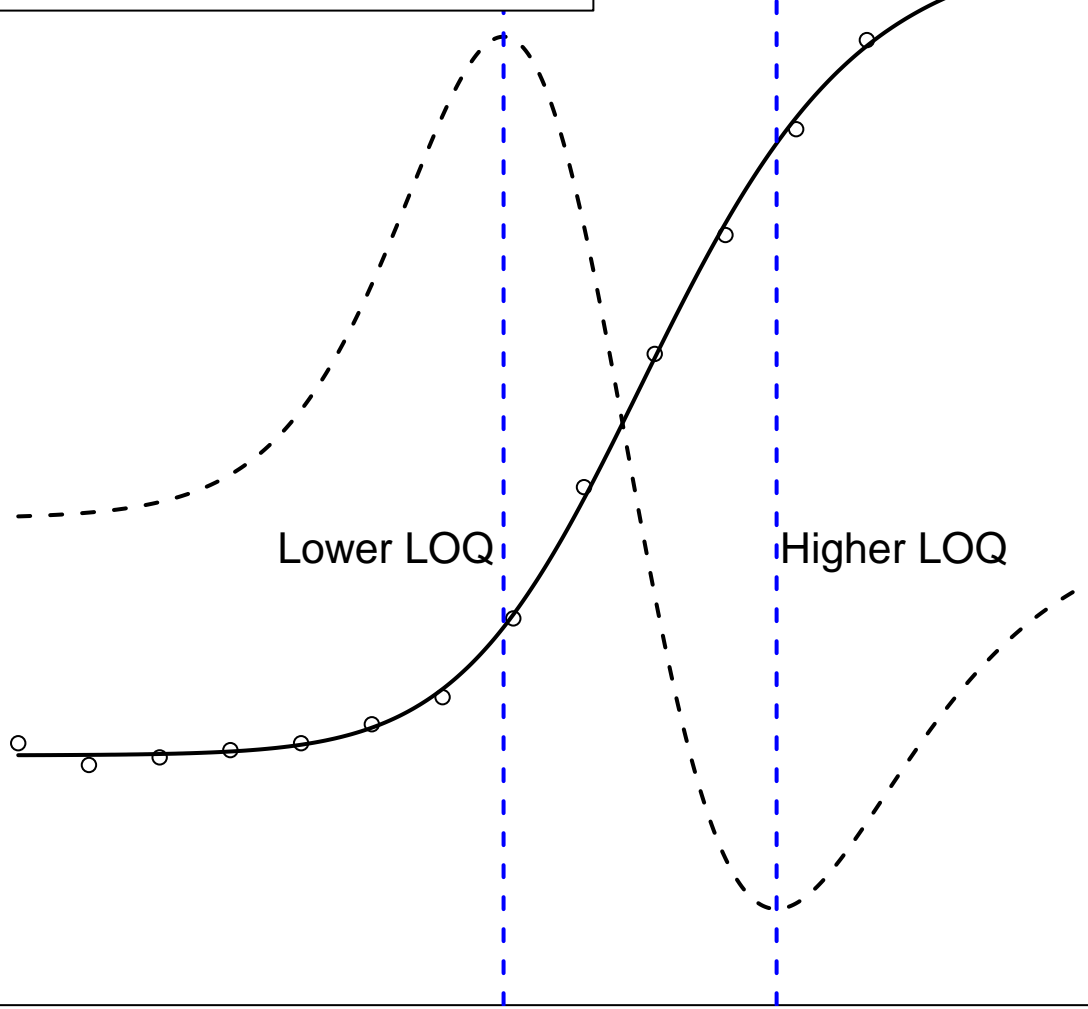

Supplement: S2 File — (TAR) [file pone.0187901.s002.tar › drLumi/vignettes/loqder.pdf]

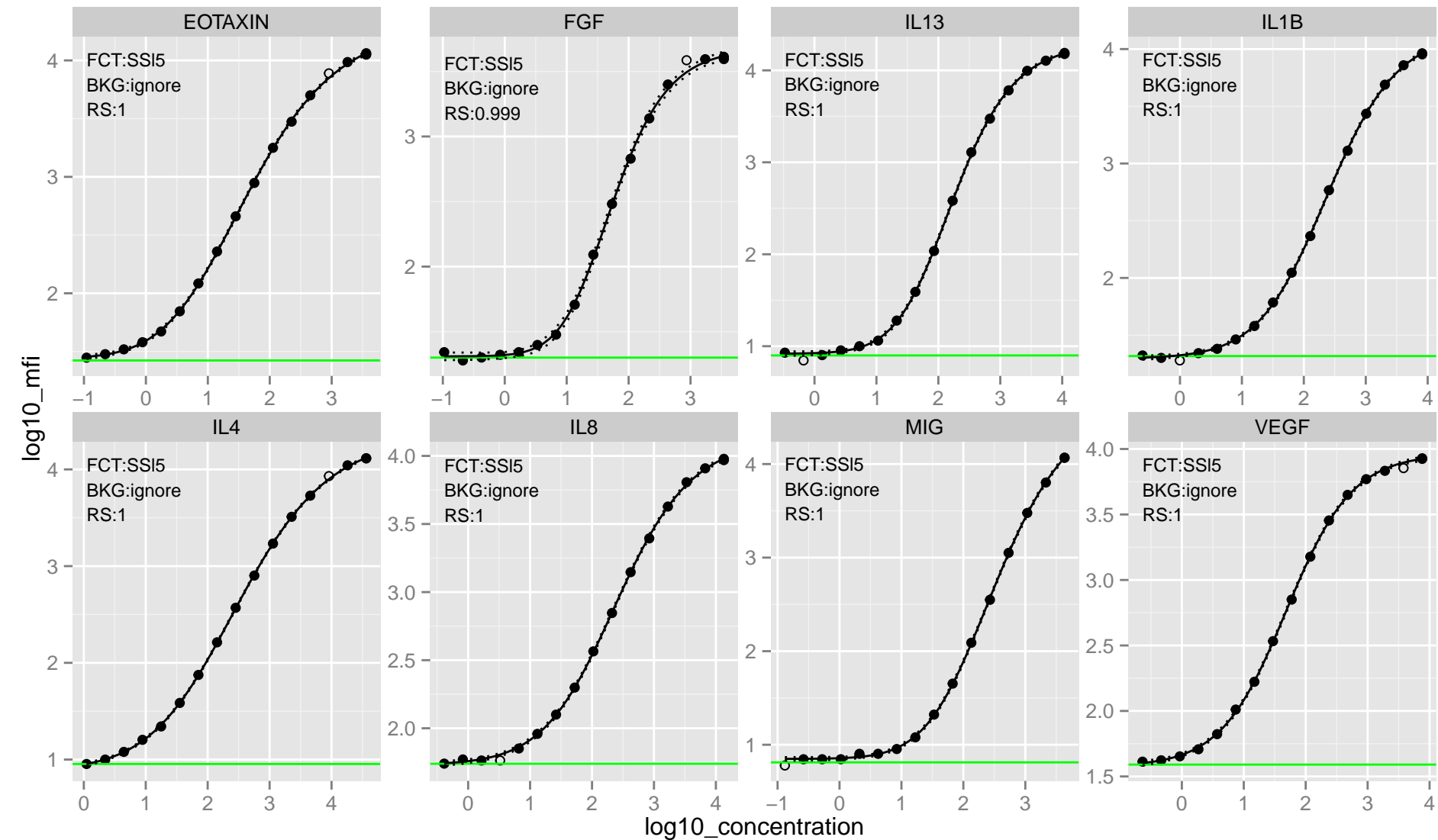

Supplement: S2 File — (TAR) [file pone.0187901.s002.tar › drLumi/vignettes/unnamed-chunk-57-1.pdf]

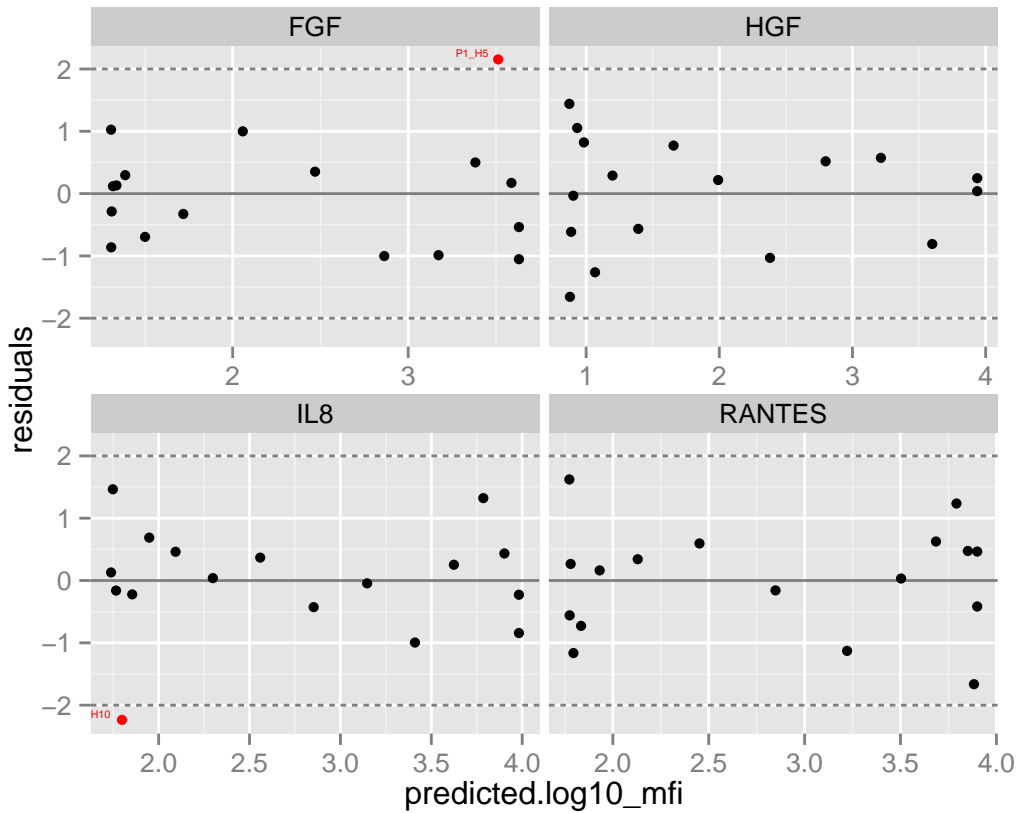

Supplement: S2 File — (TAR) [file pone.0187901.s002.tar › drLumi/vignettes/resplot.pdf]

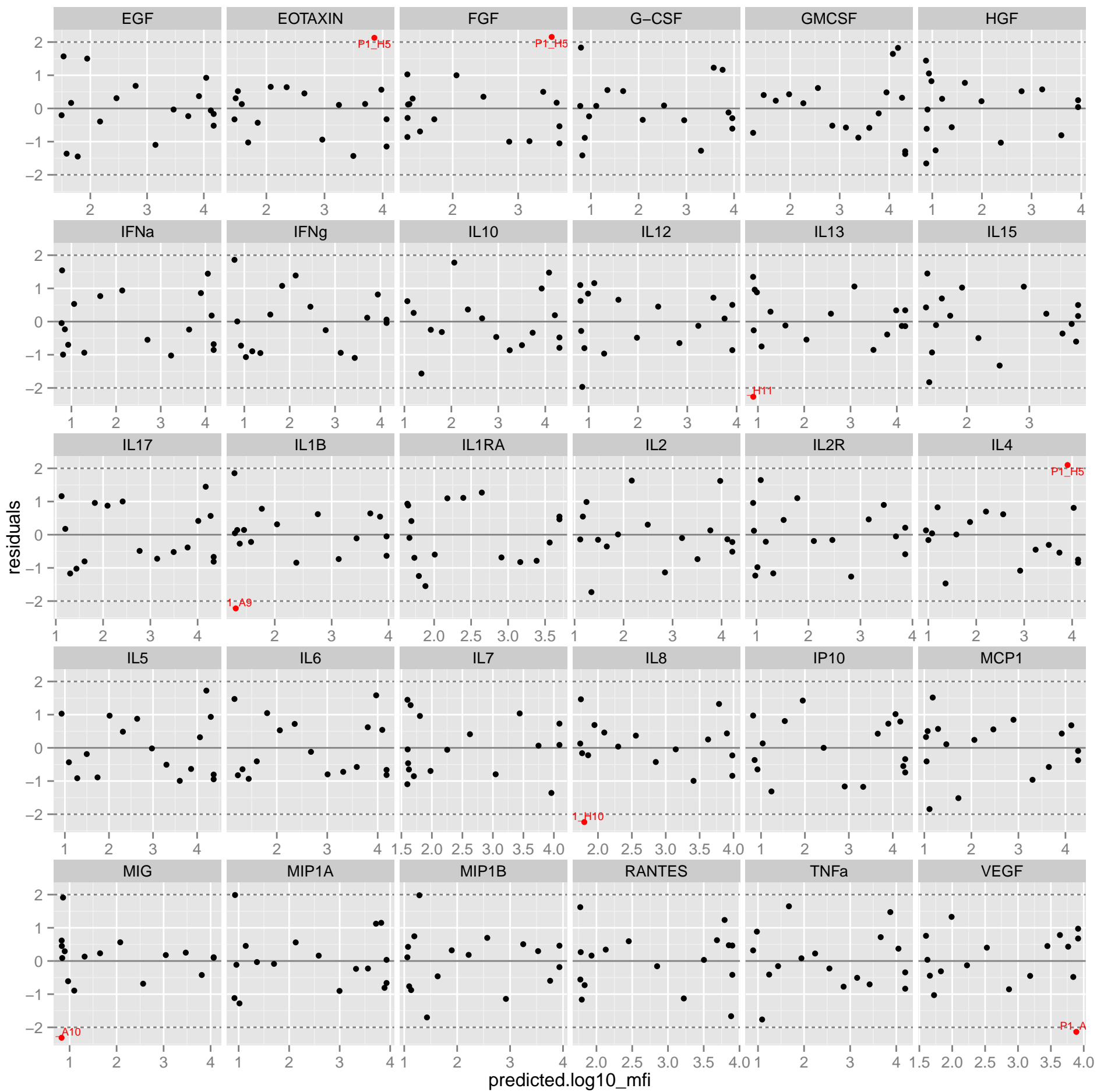

Supplement: S2 File — (TAR) [file pone.0187901.s002.tar › drLumi/vignettes/unnamed-chunk-54-1.pdf]

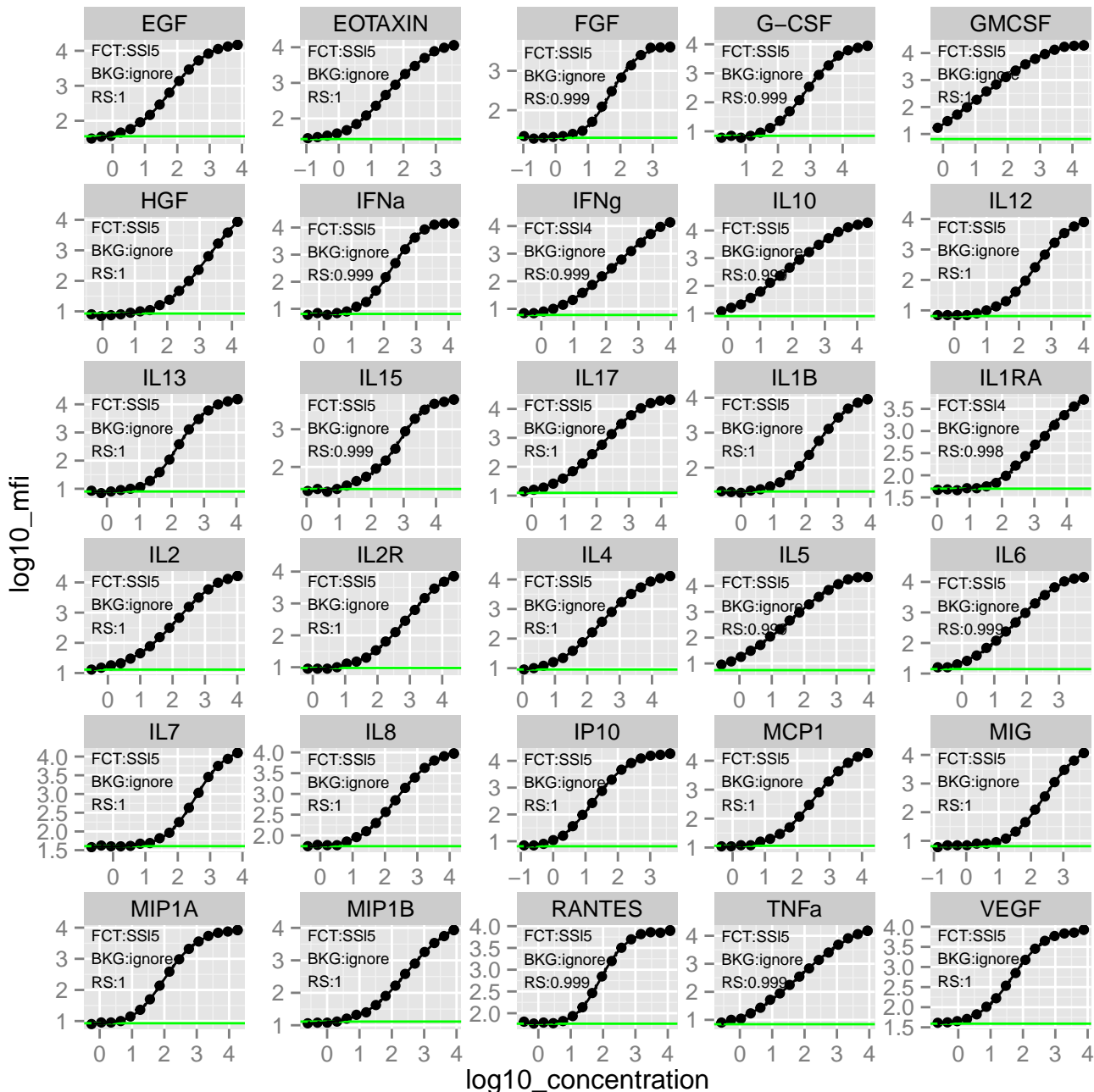

Supplement: S2 File — (TAR) [file pone.0187901.s002.tar › drLumi/vignettes/unnamed-chunk-38.pdf]

# 4 parameter logistic function

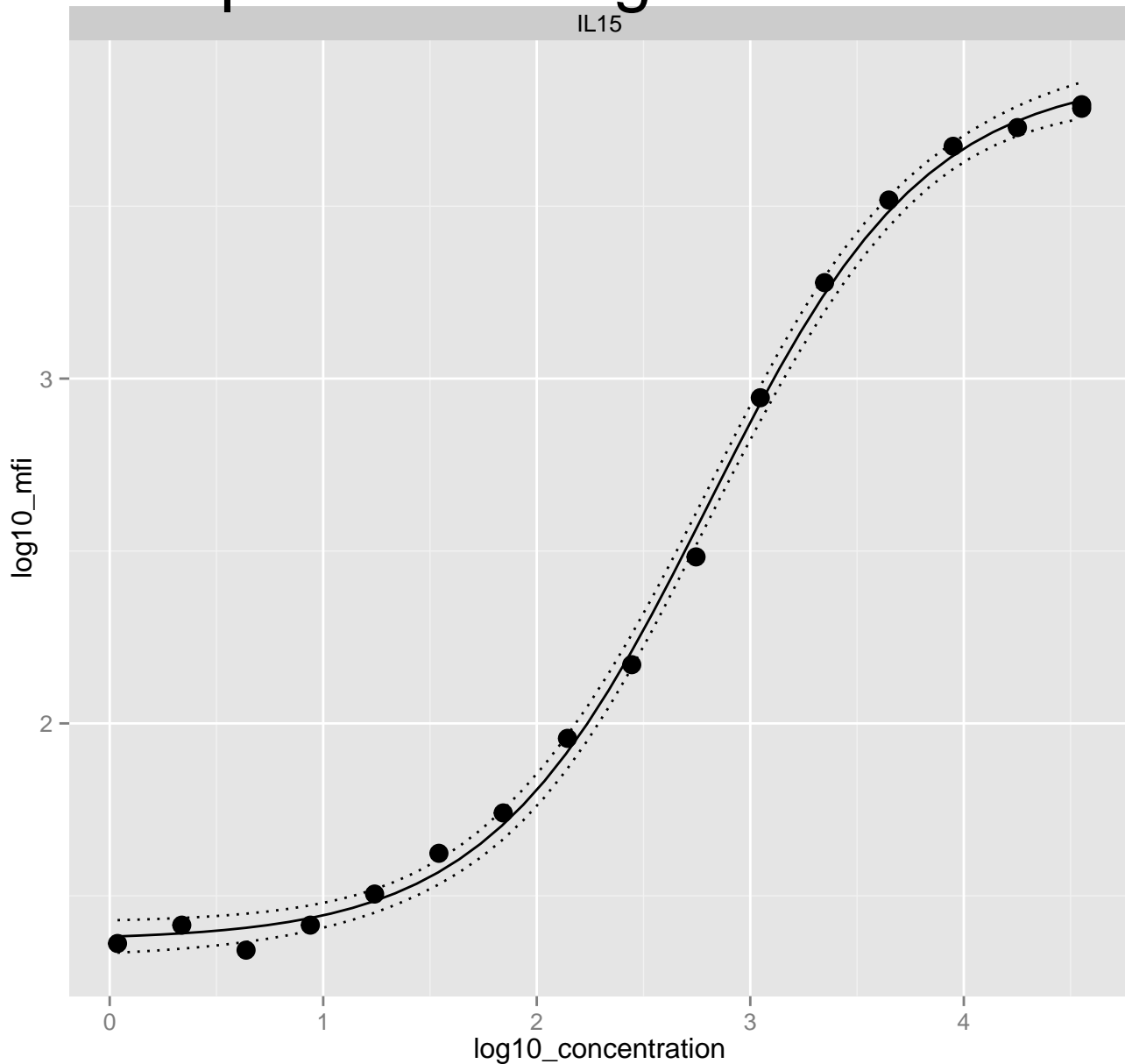

Supplement: S2 File — (TAR) [file pone.0187901.s002.tar › drLumi/vignettes/unnamed-chunk-14.pdf]

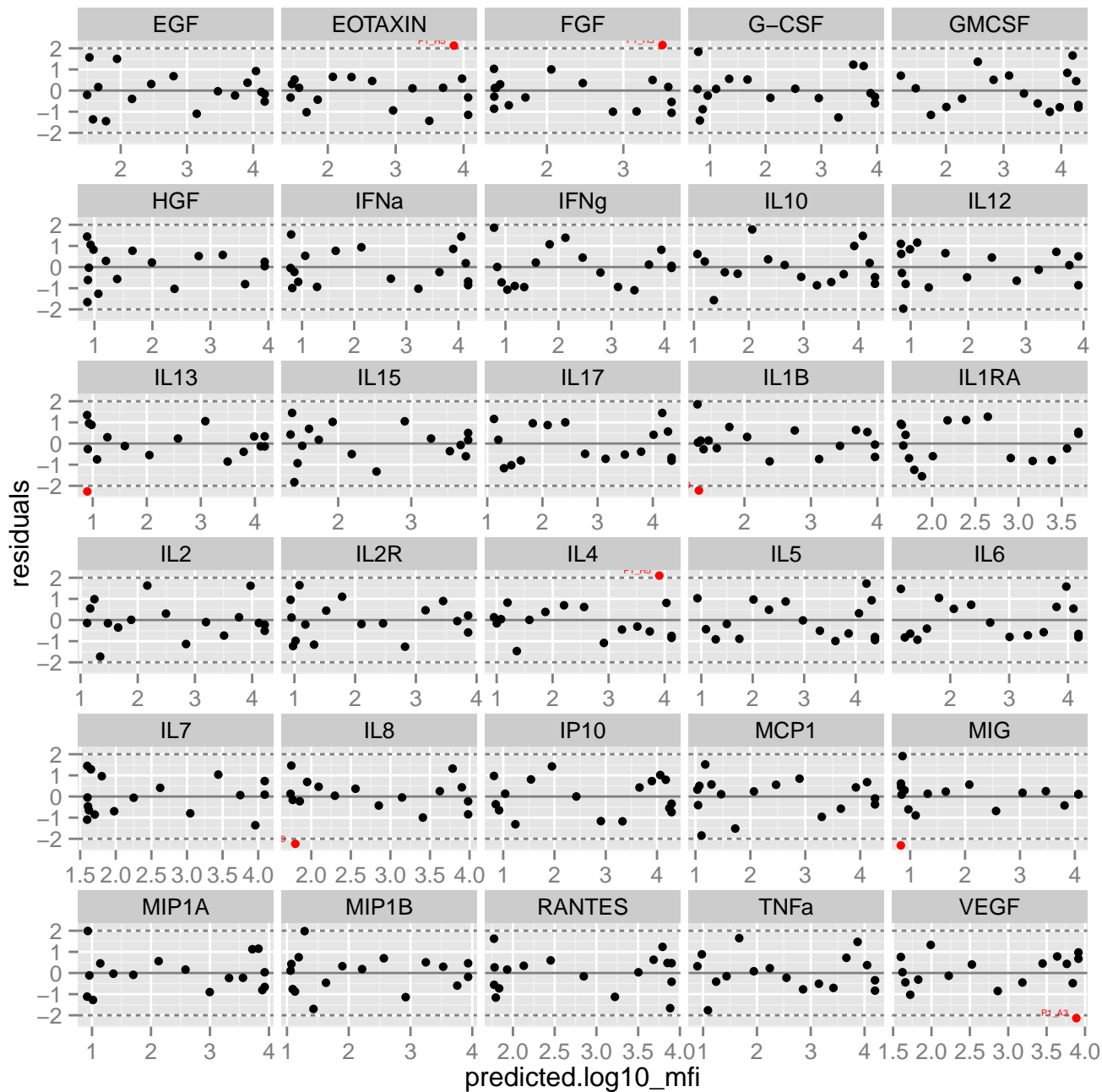

Supplement: S2 File — (TAR) [file pone.0187901.s002.tar › drLumi/vignettes/unnamed-chunk-37.pdf]

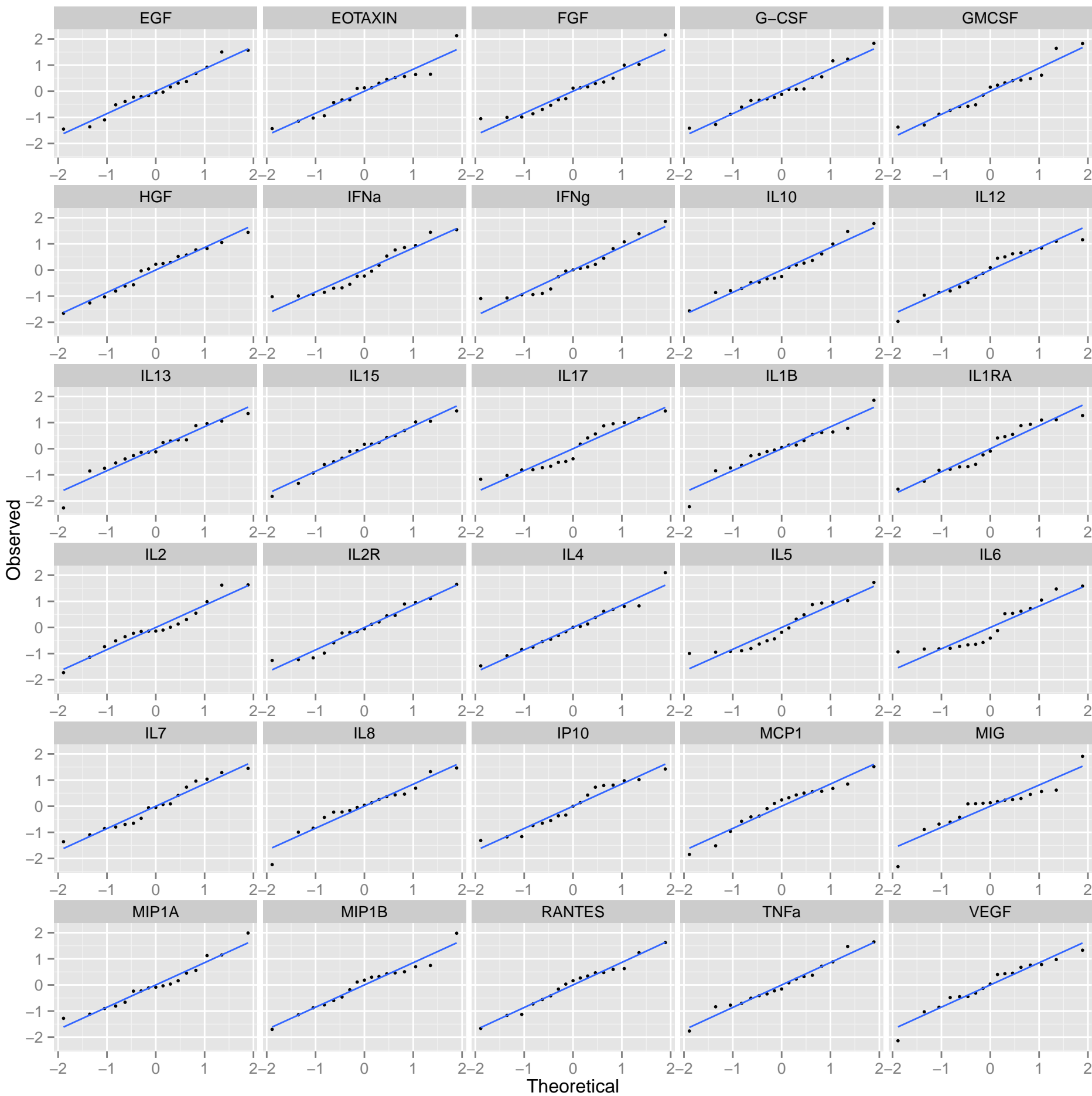

Supplement: S2 File — (TAR) [file pone.0187901.s002.tar › drLumi/vignettes/unnamed-chunk-52-1.pdf]

# 5 parameter logistic function

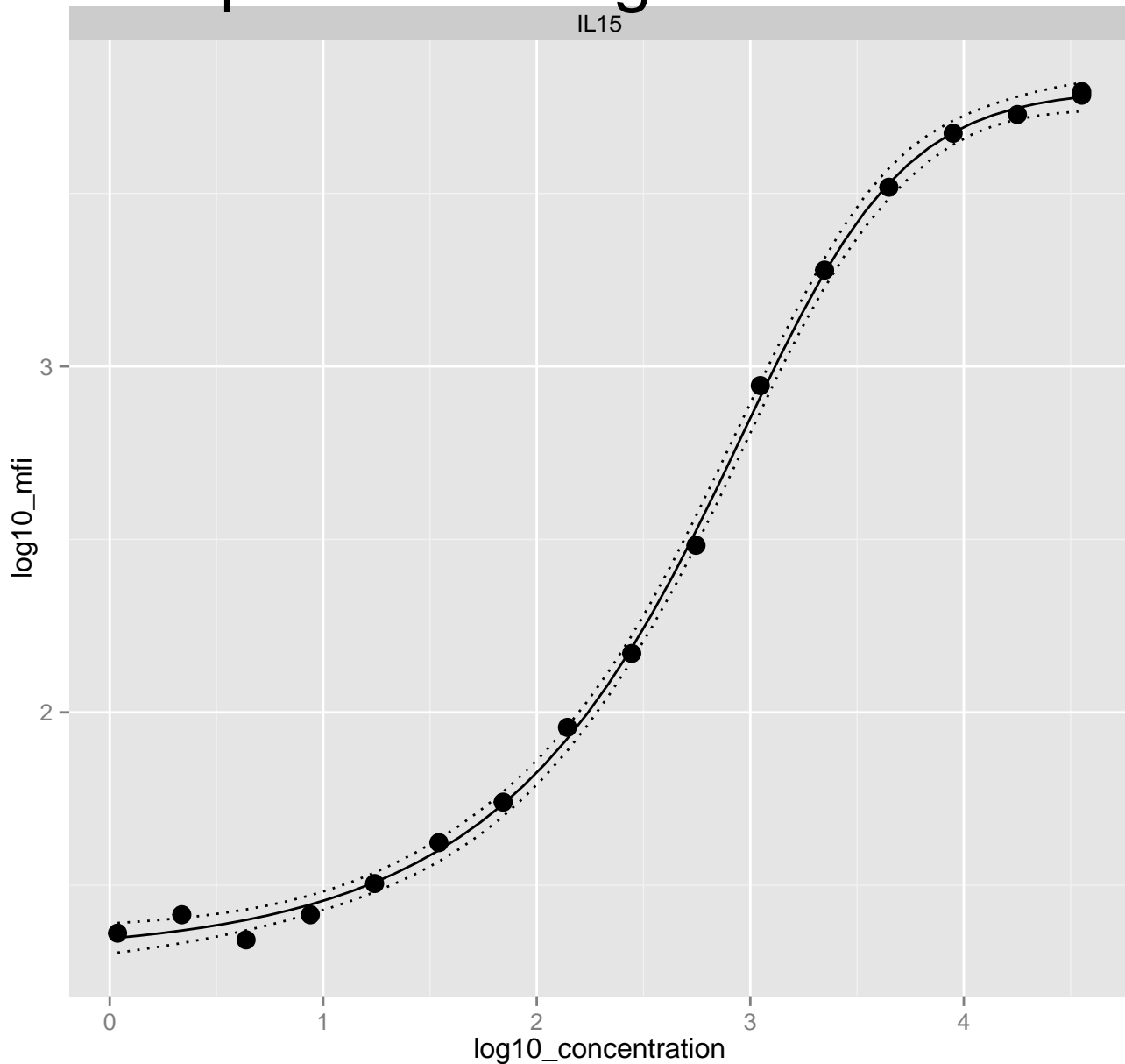

Supplement: S2 File — (TAR) [file pone.0187901.s002.tar › drLumi/vignettes/unnamed-chunk-13.pdf]

Ignored

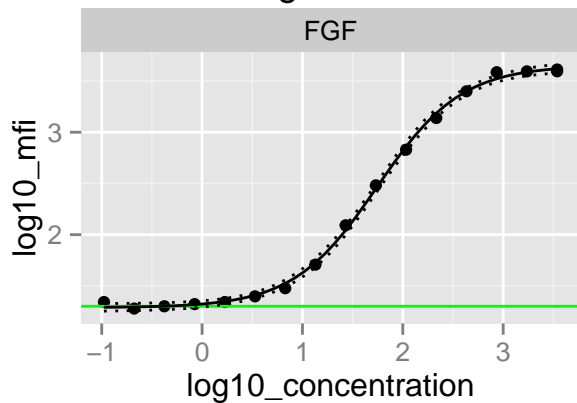

Subtracted

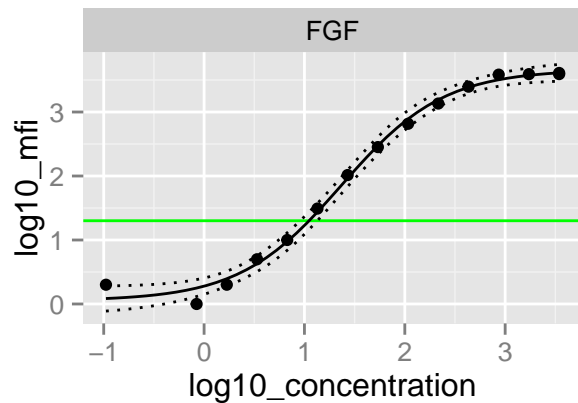

Included

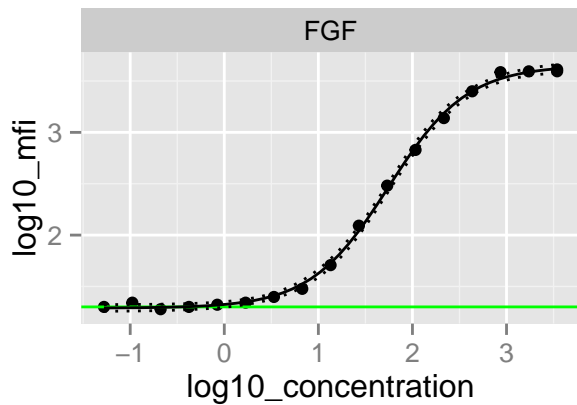

Constrained

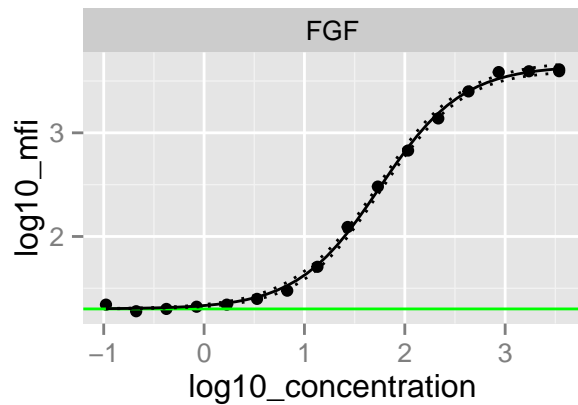

Supplement: S2 File — (TAR) [file pone.0187901.s002.tar › drLumi/vignettes/comparisonbackground.pdf]

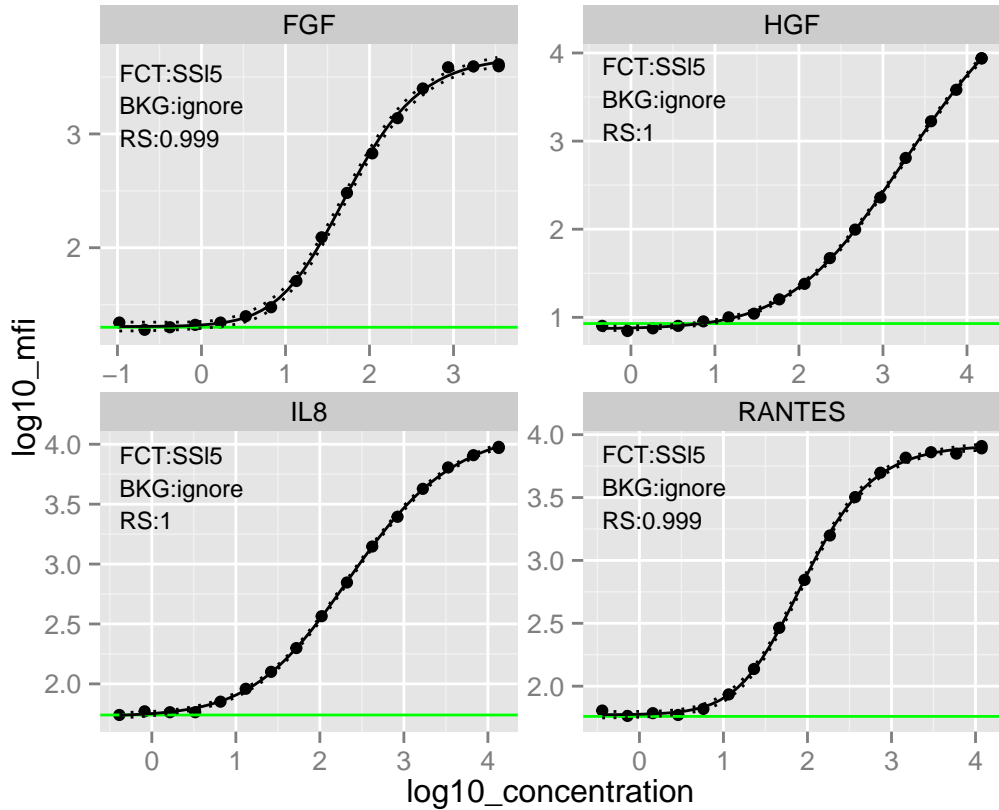

Supplement: S2 File — (TAR) [file pone.0187901.s002.tar › drLumi/vignettes/scurveplot.pdf]

# 5 parameter logistic function

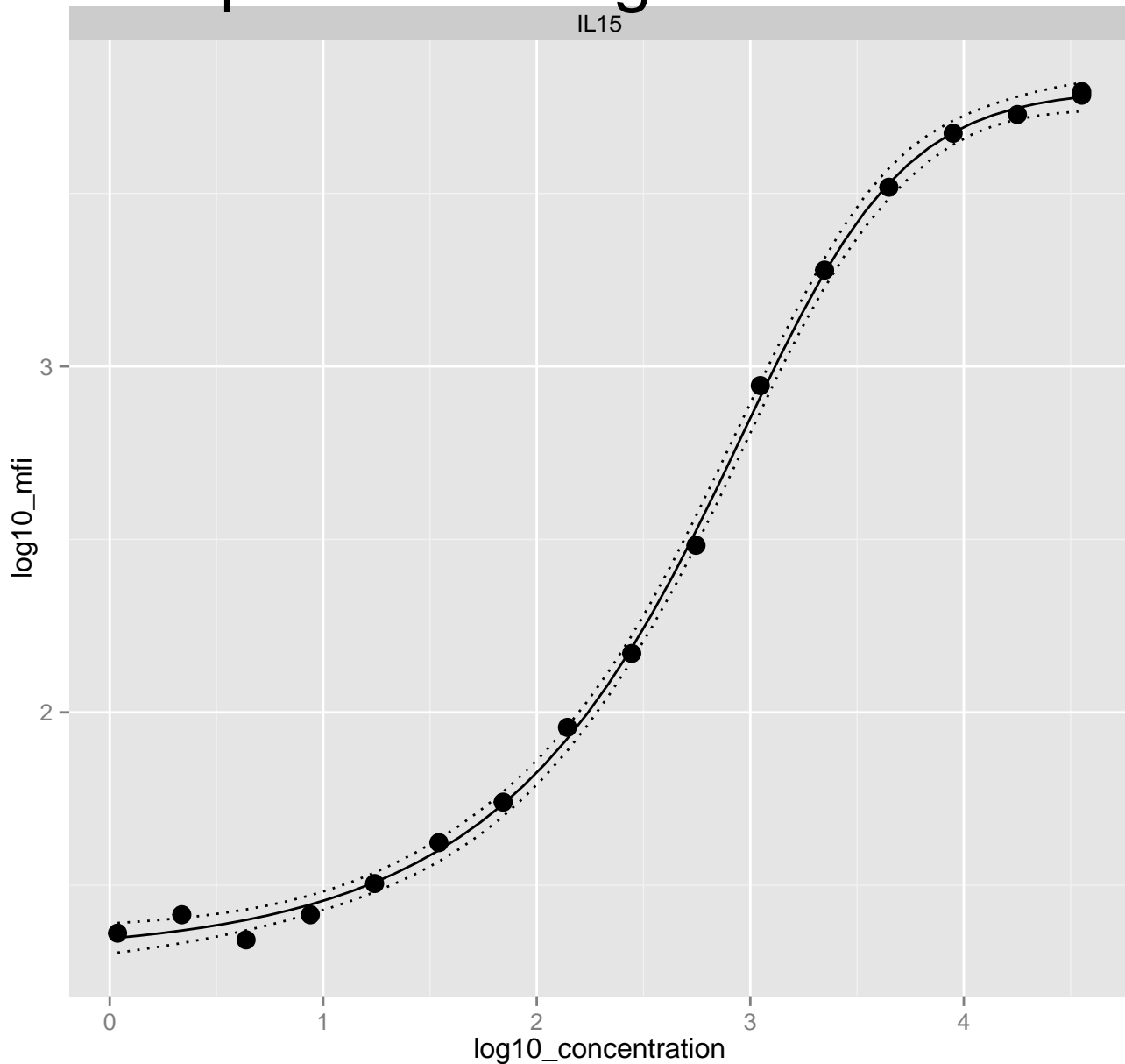

Supplement: S2 File — (TAR) [file pone.0187901.s002.tar › drLumi/vignettes/unnamed-chunk-12.pdf]

FGF

$\log_{10\_mfi}$

$\log_{10\_concentration}$

3

2

1

0

-1

0

1

2

3

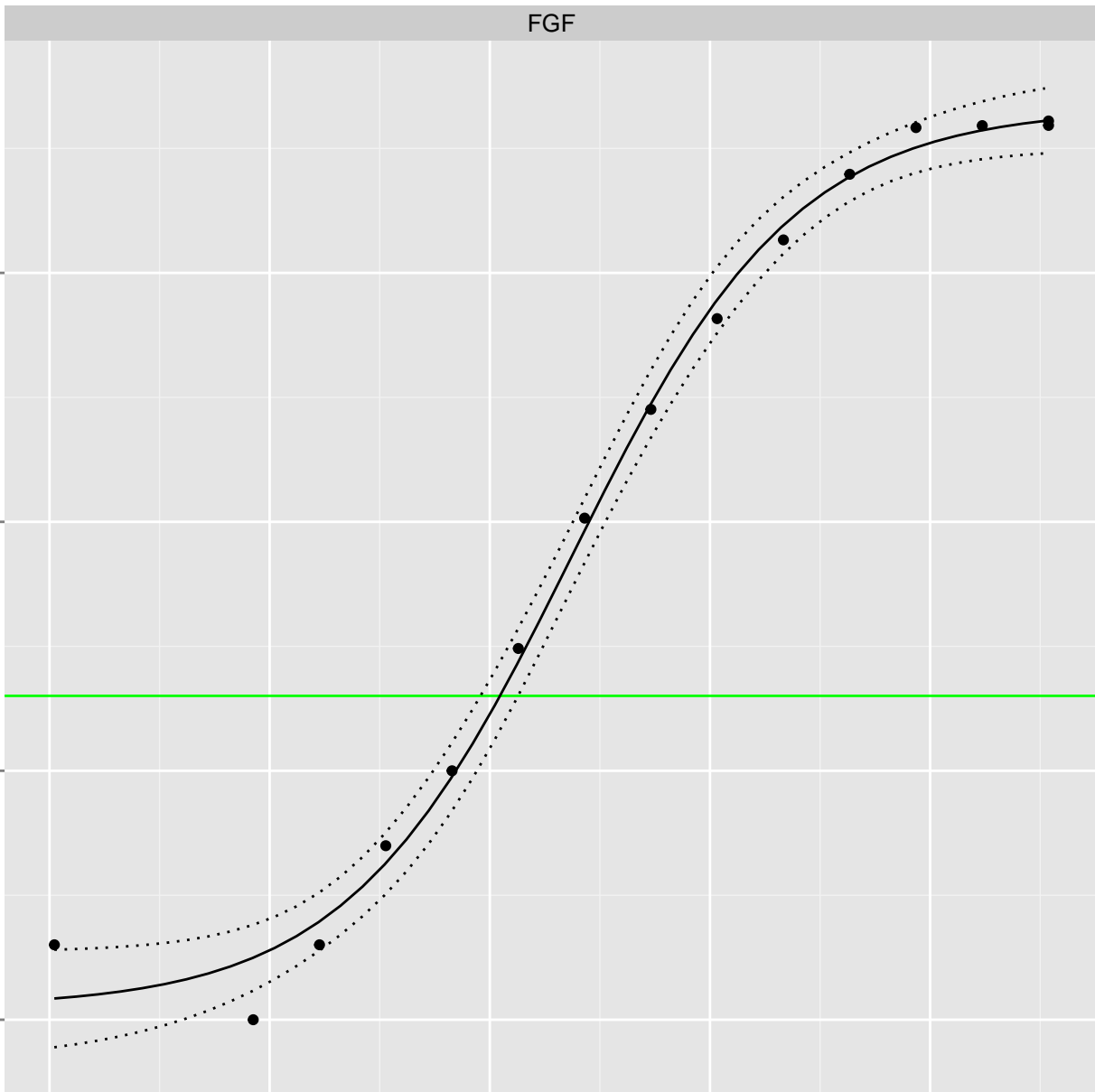

Supplement: S2 File — (TAR) [file pone.0187901.s002.tar › drLumi/vignettes/backcomparison.pdf]

# 4 parameter logistic function

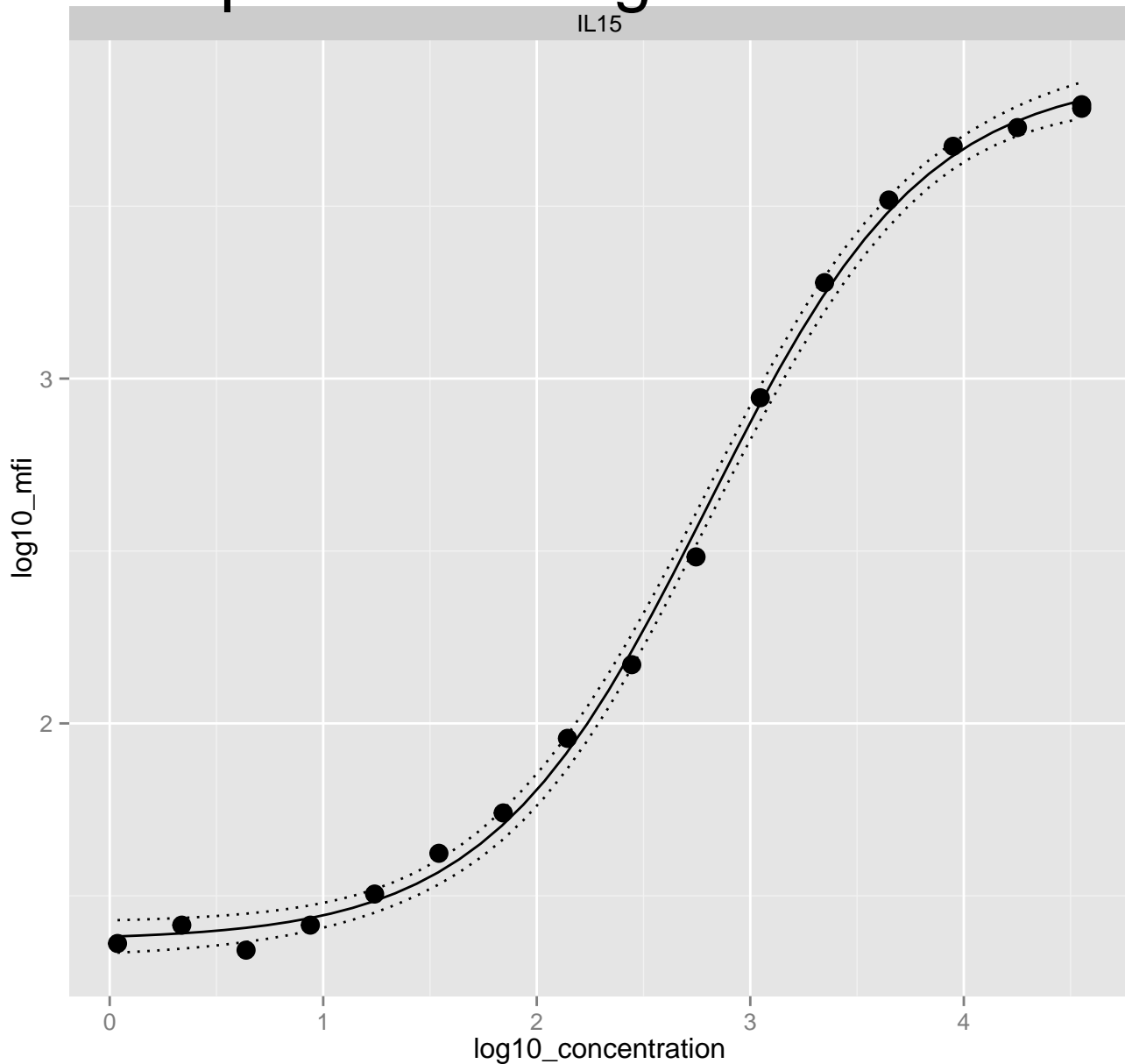

Supplement: S2 File — (TAR) [file pone.0187901.s002.tar › drLumi/vignettes/unnamed-chunk-18.pdf]

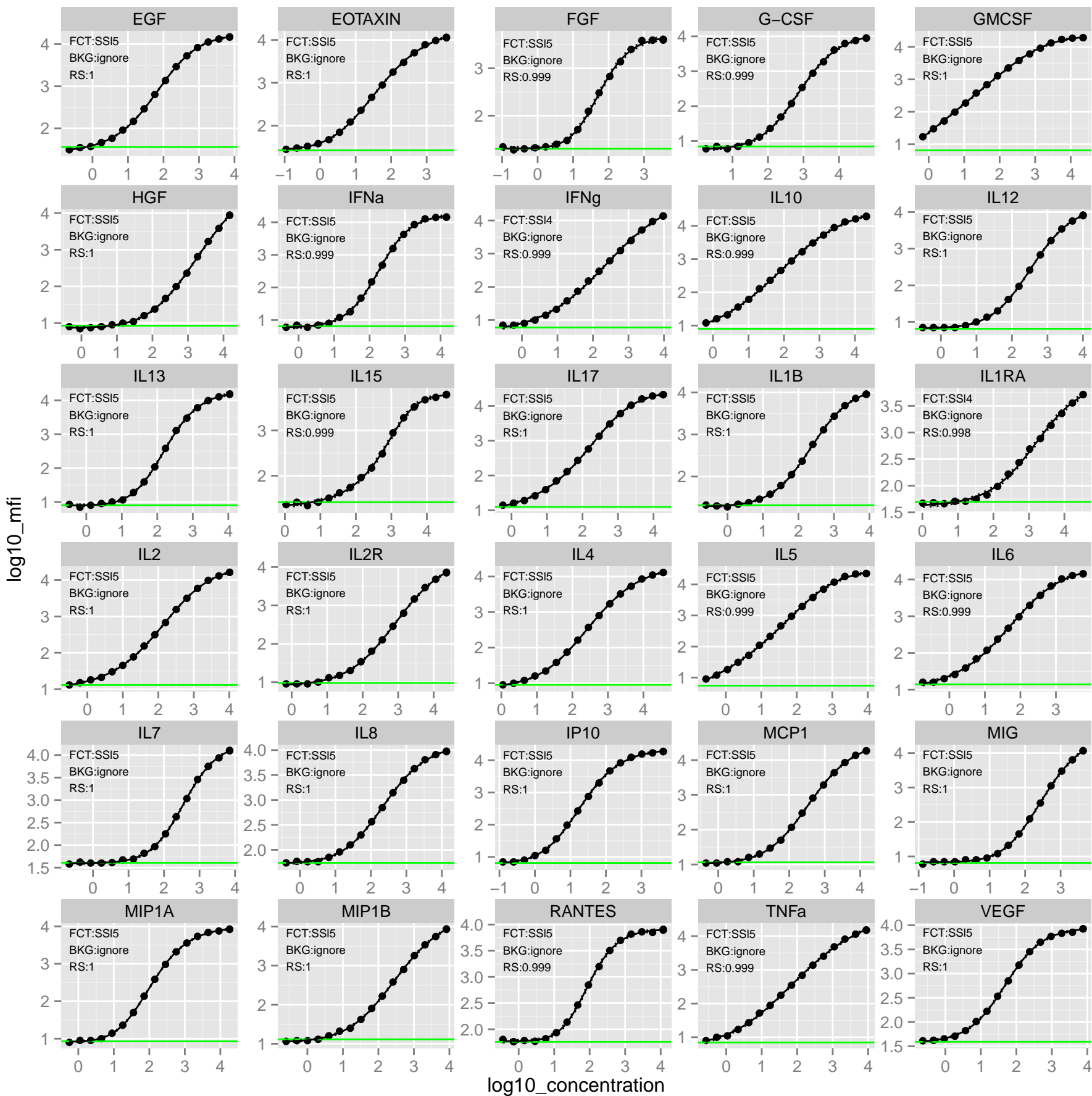

Supplement: S2 File — (TAR) [file pone.0187901.s002.tar › drLumi/vignettes/unnamed-chunk-33.pdf]
