# Supplementary material for: drLumi: An open-source package to manage data, calibrate, and conduct quality control of multiplex bead-based immunoassays data analysis
Source: PLoS One. 2017 Nov 14;12(11):e0187901. doi: 10.1371/journal.pone.0187901 (PMC5685631; doi:10.1371/journal.pone.0187901)
Supplement: S3 File — (PDF) [file pone.0187901.s003.pdf]

QA/QC results for week 5

Background substract data and flag applied

CRESIB

13th May 2014

Contents

1 Summary **4**

2 Distribution of limits of quantification and detection **5**
2.1 Minimum LLOD (MFI information) by day of the week 5
2.2 Maximum HLOD (MFI information) by day of the week 6
2.3 Maximum HLOD (MFI information) by day of the week query 7
2.4 Summary LLOD and HLOD (MFI information) over the week 8
2.5 Minimum LLOQ (MFI information) by day 9
2.6 Maximum HLOQ (MFI information) by day 10
2.7 Summary LLOQ and HLOQ (MFI information) over the week 11
2.8 Minimum LLOQ (concentration information) by day 12
2.9 Maximum HLOQ (concentration information) by day 13
2.10 Summary LLOQ and HLOQ (concentration information) over the week 14

3 Sample quality plots. **15**
3.1 PHA-DMSO ratio by analyte and site (MFI information) 15
3.2 PHA-DMSO ratio values query 16

4 Distribution of outliers **17**
4.1 Number of flags identified by plate 17
4.2 Number of flags identified by day 18
4.3 Number of outliers identified after flag by plate 19
4.4 Number of outliers identified after flag by day 20

5 Qualification and fitness of Standard Curve **21**

6 Operator assesment **22**
6.1 Intra-operator agreement 22
6.1.1 Intraclass Correlation Coefficient (agreement) results by operator 22
6.1.2 Intraclass Correlation Coefficient (agreement) query 23
6.2 Inter-operator agreement 24
6.2.1 Intraclass Correlation Coefficient (agreement) results across operators 24
6.2.2 Intraclass Correlation Coefficient (agreement) results across operators query 25
6.3 LLOD and HLOD (MFI information) 26
6.3.1 Median values for LLOD and HLOD (MFI information) 26
6.3.2 LLOD (MFI information) descriptives 27
6.3.3 HLOD (MFI information) descriptives 28
6.4 LLOQ and HLOQ (MFI information) 29
6.4.1 Median values for LLOQ and HLOQ (MFI information) 29
6.4.2 LLOQ (MFI information) descriptives 30
6.4.3 HLOQ (MFI information) descriptives 31
6.5 LLOQ and HLOQ (concentration information) 32
6.5.1 Median values for LLOQ and HLOQ (concentration information) 32
6.5.2 LLOQ (concentration information) descriptives 33
6.5.3 HLOQ (concentration information) descriptives 34

7 Assay random and systematic variability **35**
7.1 Mean concentration for controls by plate and analyte 35
7.2 Coefficient of variation by day 38
7.3 Coefficient of variation by operator 39
7.4 Coefficient of variation query 40
7.5 Agreement for positive controls 41

8 Annex **43**
8.1 Summary of queries by plate (taking into account all analytes) . . . 43
8.2 CSP stimulated samples boxplots by analytes . . . 44
8.3 CSP stimulated samples boxplots by analytes and plate . . . 45
8.4 Samples boxplots by analytes and stimulation . . . 46
8.5 Samples boxplots by analytes query . . . 47
8.6 Standard curve and Residuals plots by analyte and stimulation . . . 48

1 Summary

- Background method applied: substract.
- Week: 5.
- Operators number: 3.
- Plates number: 3.

Distribution Operator/Plate:

	m067_37	m067_38	m067_39	m067_40	m067_41	m067_42	m067_43	m067_44	m067_45
1	1	0	0	1	0	0	1	0	0
2	0	0	1	0	1	0	0	1	0
4	0	1	0	0	0	1	0	0	1

2.4 Summary LLOD and HLOD (MFI information) over the week

	Blank			LLOD			HLOD			
	Min	Median	Max	Min	Median	Max	Min	Median	Max	N.Plates
EGF	28.5	41.2	50.5	28.8	42.7	50.5	17090	22510	25000	9
EOTAXIN	19.0	28.5	37.0	20.0	28.5	37.0	20670	25000	25000	9
FGF	18.5	26.0	32.5	19.9	26.5	32.5	5067	5799	7192	9
G-CSF	6.0	7.0	10.0	6.5	8.0	11.0	12710	16660	20290	9
GMCSF	5.0	6.0	7.5	5.0	6.0	7.5	25000	25000	25000	9
HGF	7.0	8.5	10.0	8.0	8.9	11.0	25000	25000	25000	9
IFNa	5.0	6.5	8.5	5.7	6.9	8.6	16520	20510	25000	9
IFNg	5.0	6.5	8.5	5.5	7.0	8.5	25000	25000	25000	9
IL10	6.5	8.0	9.5	6.5	8.0	9.5	25000	25000	25000	9
IL12	6.0	7.0	9.0	6.0	8.0	10.0	20940	25000	25000	9
IL13	6.5	8.0	10.0	6.8	8.8	10.7	18300	25000	25000	9
IL15	22.5	29.8	42.5	22.7	29.8	42.5	5992	6989	25000	9
IL17	9.5	11.5	15.5	9.5	11.6	15.5	25000	25000	25000	9
IL1B	19.8	25.0	31.0	20.9	26.0	31.0	15940	23510	25000	9
IL1RA	42.8	58.0	81.5	42.8	60.0	81.5	8995	14140	25000	9
IL2	13.0	17.2	22.0	13.2	17.3	22.0	25000	25000	25000	9
IL2R	8.0	10.0	13.5	9.0	11.0	13.5	25000	25000	25000	9
IL4	7.5	9.0	11.0	7.6	9.1	11.1	19780	25000	25000	9
IL5	5.0	6.0	7.5	5.0	6.0	7.5	25000	25000	25000	9
IL6	12.5	16.5	21.5	12.5	16.7	21.5	25000	25000	25000	9
IL7	32.5	43.0	55.2	32.5	43.8	55.2	18420	25000	25000	9
IL8	50.0	65.0	94.5	50.6	65.1	94.5	15630	25000	25000	9
IP10	6.0	7.0	9.8	6.5	8.0	10.6	23680	25000	25000	9
MCP1	11.0	13.5	19.0	12.0	13.5	20.8	25000	25000	25000	9
MIG	5.5	6.5	9.5	5.9	7.2	9.5	25000	25000	25000	9
MIP1A	7.0	10.0	12.0	7.9	10.0	13.0	9884	10850	13590	9
MIP1B	11.5	14.2	20.0	12.3	15.2	20.1	25000	25000	25000	9
RANTES	48.5	58.5	74.5	51.0	59.8	74.5	8437	9008	10560	9
TNFa	6.0	8.5	10.5	6.0	8.5	10.5	25000	25000	25000	9
VEGF	37.0	49.5	60.8	38.0	49.5	61.2	8442	9676	15650	9

4 Distribution of outliers

4.1 Number of flags identified by plate

	m067_37	m067_38	m067_39	m067_40	m067_41	m067_42	m067_43	m067_44	m067_45
EGF	1	0	0	1	1	0	0	0	0
EOTAXIN	0	0	0	0	0	0	0	0	0
FGF	0	0	0	0	0	0	0	0	0
G-CSF	0	0	0	0	0	1	0	0	0
GMCSF	0	0	0	0	0	0	0	0	0
HGF	0	0	0	0	0	0	0	0	0
IFNa	0	0	0	0	0	0	0	0	0
IFNg	0	0	1	0	0	1	1	1	1
IL10	0	0	0	0	0	0	0	0	0
IL12	0	0	1	0	0	0	0	0	0
IL13	0	0	0	0	1	1	0	0	0
IL15	0	1	0	0	0	0	0	0	0
IL17	0	0	0	0	0	0	0	0	0
IL1B	0	0	0	0	1	1	0	0	0
IL1RA	0	0	1	0	0	0	0	0	0
IL2	0	1	0	0	1	0	0	1	0
IL2R	0	0	0	0	0	0	0	0	0
IL4	0	1	0	0	1	0	1	0	0
IL5	0	0	0	0	0	0	0	0	0
IL6	0	0	0	0	0	0	0	0	0
IL7	0	0	0	0	0	0	0	0	0
IL8	0	1	0	0	0	1	0	0	0
IP10	0	0	0	0	1	0	0	0	0
MCP1	0	1	0	1	0	0	0	0	0
MIG	0	0	0	0	0	0	0	0	0
MIP1A	1	0	1	0	1	0	0	1	0
MIP1B	1	0	1	1	0	0	0	0	0
RANTES	0	1	0	0	0	0	0	0	0
TNFa	0	1	0	0	0	0	0	0	0
VEGF	1	0	0	0	1	1	0	0	0

4.3 Number of outliers identified after flag by plate

	m067_37	m067_38	m067_39	m067_40	m067_41	m067_42	m067_43	m067_44	m067_45
EGF	0	0	0	0	0	0	0	0	0
EOTAXIN	0	0	0	0	0	0	0	0	0
FGF	0	0	0	0	0	0	0	0	0
G-CSF	0	0	0	0	0	0	0	0	0
GMCSF	0	0	0	0	0	0	0	0	0
HGF	0	0	0	0	0	0	0	0	0
IFNa	0	0	0	0	0	0	0	0	0
IFNg	0	0	0	0	0	0	0	0	0
IL10	0	0	0	0	0	0	0	0	0
IL12	0	0	0	0	0	0	0	0	0
IL13	0	0	0	0	0	0	0	0	0
IL15	0	0	0	0	0	0	0	0	0
IL17	0	0	0	0	0	0	0	0	0
IL1B	0	0	0	0	0	0	0	0	0
IL1RA	0	0	0	0	0	0	0	0	0
IL2	0	0	0	0	0	0	0	0	0
IL2R	0	0	0	0	0	0	0	0	0
IL4	0	0	0	0	0	0	0	0	0
IL5	0	0	0	0	0	0	0	0	0
IL6	0	0	0	0	0	0	0	0	0
IL7	0	0	0	0	0	0	0	0	0
IL8	0	0	0	0	0	0	0	0	0
IP10	0	0	0	0	0	0	0	0	0
MCP1	0	0	0	0	0	0	0	0	0
MIG	0	0	0	0	0	0	0	0	0
MIP1A	0	0	0	0	1	0	0	0	0
MIP1B	0	0	0	0	0	0	0	0	0
RANTES	0	0	0	0	0	0	0	0	0
TNFa	0	0	0	0	0	0	0	0	0
VEGF	0	0	0	0	0	0	0	0	0

5 Qualification and fitness of Standard Curve

Analyte	N.Plates	$R^2 \leq 0.95$	Mod.Fit$\leq$ 0.05	B pval $\leq$ 0.05	N.Convergence	N.LL5 conv.	N.LL4 conv.
EGF	9	0	1	9	9	9	0
EOTAXIN	9	0	0	9	9	9	0
FGF	9	0	0	8	9	9	0
G-CSF	9	0	0	9	9	9	0
GMCSF	9	0	0	9	9	9	0
HGF	9	0	0	8	9	9	0
IFNa	9	0	0	9	9	9	0
IFNg	9	0	0	9	9	9	0
IL10	9	0	0	9	9	9	0
IL12	9	0	2	8	9	9	0
IL13	9	0	0	9	9	8	1
IL15	9	0	0	6	9	9	0
IL17	9	0	0	8	9	9	0
IL1B	9	0	0	9	9	9	0
IL1RA	9	0	0	8	9	9	0
IL2	9	0	0	9	9	9	0
IL2R	9	0	0	9	9	9	0
IL4	9	0	0	9	9	8	1
IL5	9	0	0	9	9	9	0
IL6	9	0	0	8	9	8	1
IL7	9	0	0	8	9	9	0
IL8	9	0	0	8	9	9	0
IP10	9	0	0	9	9	8	1
MCP1	9	0	1	9	9	9	0
MIG	9	0	0	7	9	8	1
MIP1A	9	0	0	9	9	9	0
MIP1B	9	0	0	9	9	9	0
RANTES	9	0	1	9	9	9	0
TNFa	9	0	0	9	9	9	0
VEGF	9	0	0	8	9	9	0

6 Operator assesment

6.1 Intra-operator agreement

6.1.1 Intraclass Correlation Coefficient (agreement) results by operator

	Operator1			Operator2			Operator3		
	N.Plates	Min	Overall	N.Plates	Min	Overall	N.Plates	Min	Overall
EGF	3	1.000	1.000	3	0.999	0.999	3	1.000	1.000
EOTAXIN	3	0.997	0.999	3	0.987	0.995	3	0.999	0.999
FGF	3	0.998	0.999	3	0.994	0.998	3	0.993	0.996
G.CSF	3	0.996	0.998	3	0.989	0.997	3	0.999	0.999
GMCSF	3	0.999	1.000	3	0.998	0.999	3	0.998	0.999
HGF	3	0.995	0.995	3	0.991	0.993	3	0.972	0.979
IFNa	3	0.999	0.999	3	0.998	0.999	3	0.999	0.999
IFNg	3	0.999	1.000	3	0.998	0.999	3	0.999	1.000
IL10	3	0.999	0.999	3	0.995	0.998	3	1.000	1.000
IL12	3	0.995	0.996	3	0.996	0.997	3	0.993	0.992
IL13	3	0.999	1.000	3	0.996	0.999	3	0.999	0.999
IL15	3	0.999	0.999	3	0.994	0.998	3	0.997	0.997
IL17	3	0.999	0.999	3	0.997	0.999	3	0.999	0.999
IL1B	3	0.996	0.999	3	0.998	0.999	3	0.995	0.996
IL1RA	3	0.997	0.997	3	0.993	0.997	3	0.992	0.993
IL2	3	0.999	0.999	3	0.998	0.999	3	0.999	0.999
IL2R	3	1.000	1.000	3	0.999	0.999	3	0.997	0.998
IL4	3	0.999	0.999	3	0.998	0.999	3	0.998	0.998
IL5	3	0.999	0.999	3	0.998	0.999	3	0.999	1.000
IL6	3	0.999	0.999	3	0.997	0.999	3	0.999	0.999
IL7	3	0.988	0.993	3	0.994	0.998	3	0.992	0.993
IL8	3	0.998	0.998	3	0.995	0.997	3	0.998	0.997
IP10	3	0.999	1.000	3	0.997	0.999	3	0.999	1.000
MCP1	3	0.997	0.999	3	0.991	0.996	3	0.998	0.999
MIG	3	0.994	0.997	3	0.995	0.996	3	0.989	0.993
MIP1A	3	0.999	0.999	3	0.992	0.998	3	0.999	0.999
MIP1B	3	1.000	1.000	3	0.997	0.998	3	0.998	0.998
RANTES	3	0.999	0.999	3	0.988	0.996	3	0.996	0.997
TNFa	3	0.999	0.999	3	0.997	0.999	3	0.997	0.997
VEGF	3	0.998	0.999	3	0.987	0.997	3	0.995	0.996

7 Assay random and systematic variability

7.1 Mean concentration for controls by plate and analyte

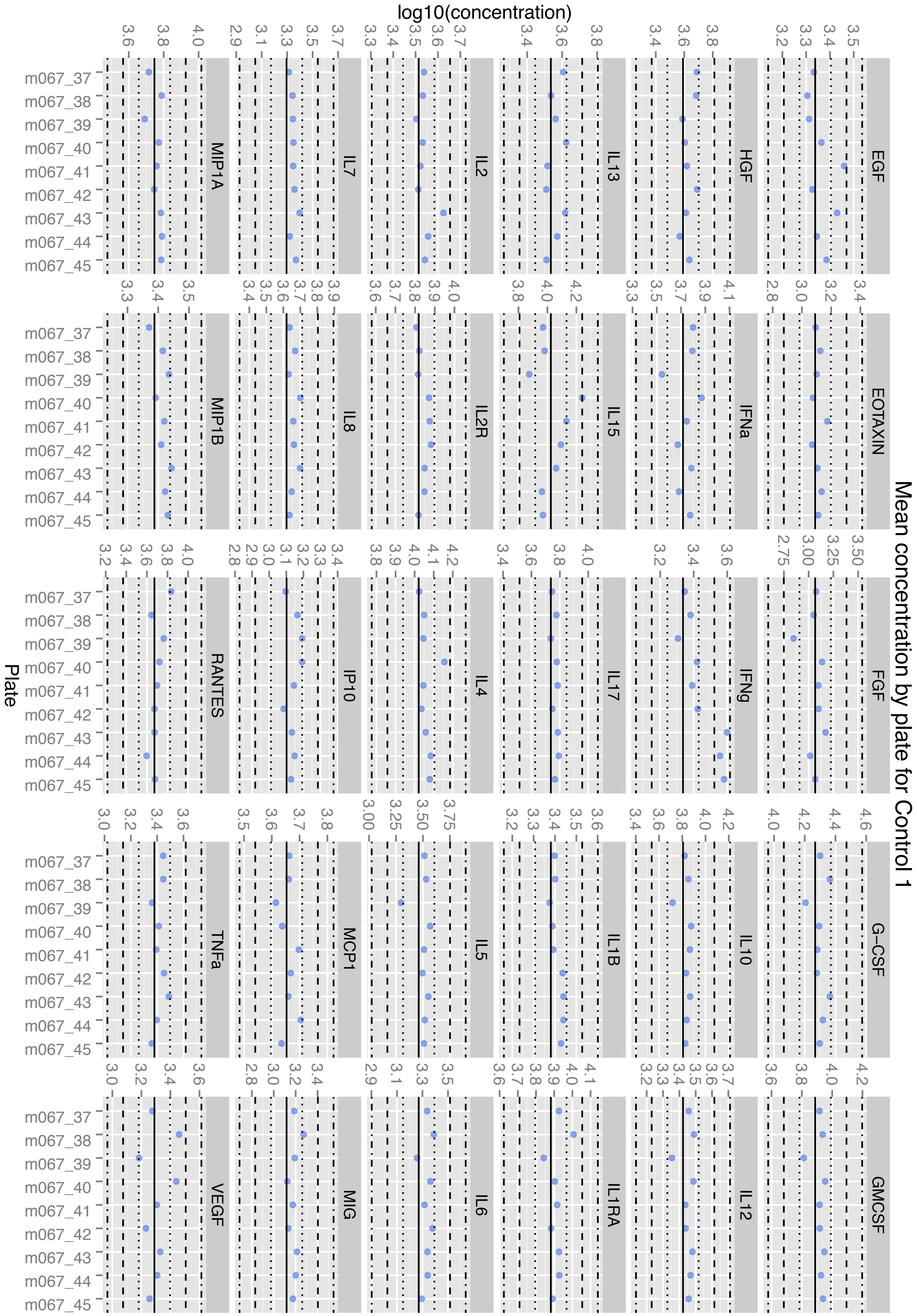

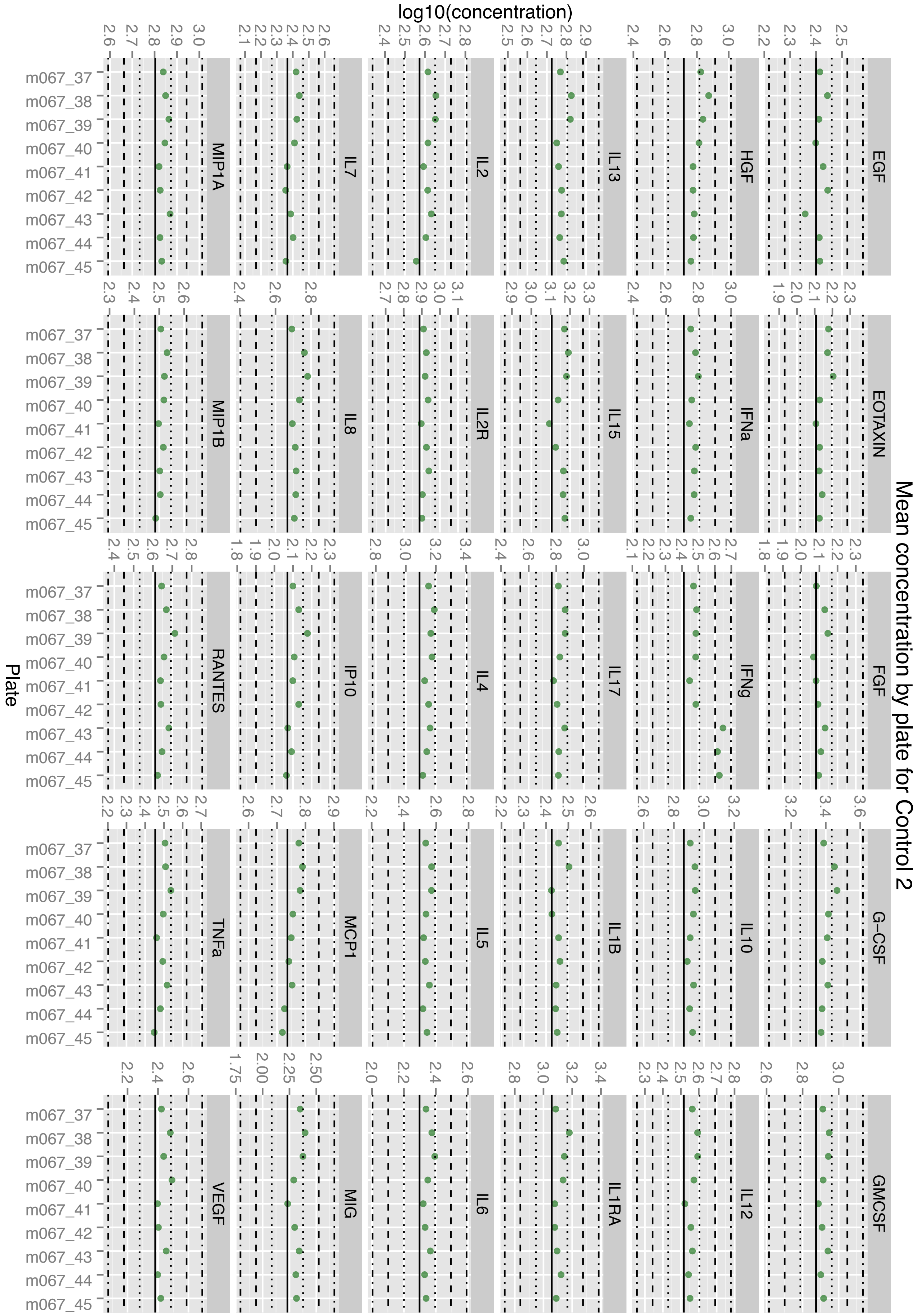
Mean concentration by plate for Control 2
log10(concentration)
Plate
m067_37
m067_38
m067_39
m067_40
m067_41
m067_42
m067_43
m067_44
m067_45
EGF
HGF
IL13
IL2
IL7
MIP1A
EOTAXIN
IFNa
IL15
IL2R
IL8
MIP1B
FGF
IFNg
IL17
IL4
IP10
RANTES
G−CSF
IL10
IL1B
IL5
MCP1
TNFa
GMCSF
IL12
IL1RA
IL6
MIG
VEGF

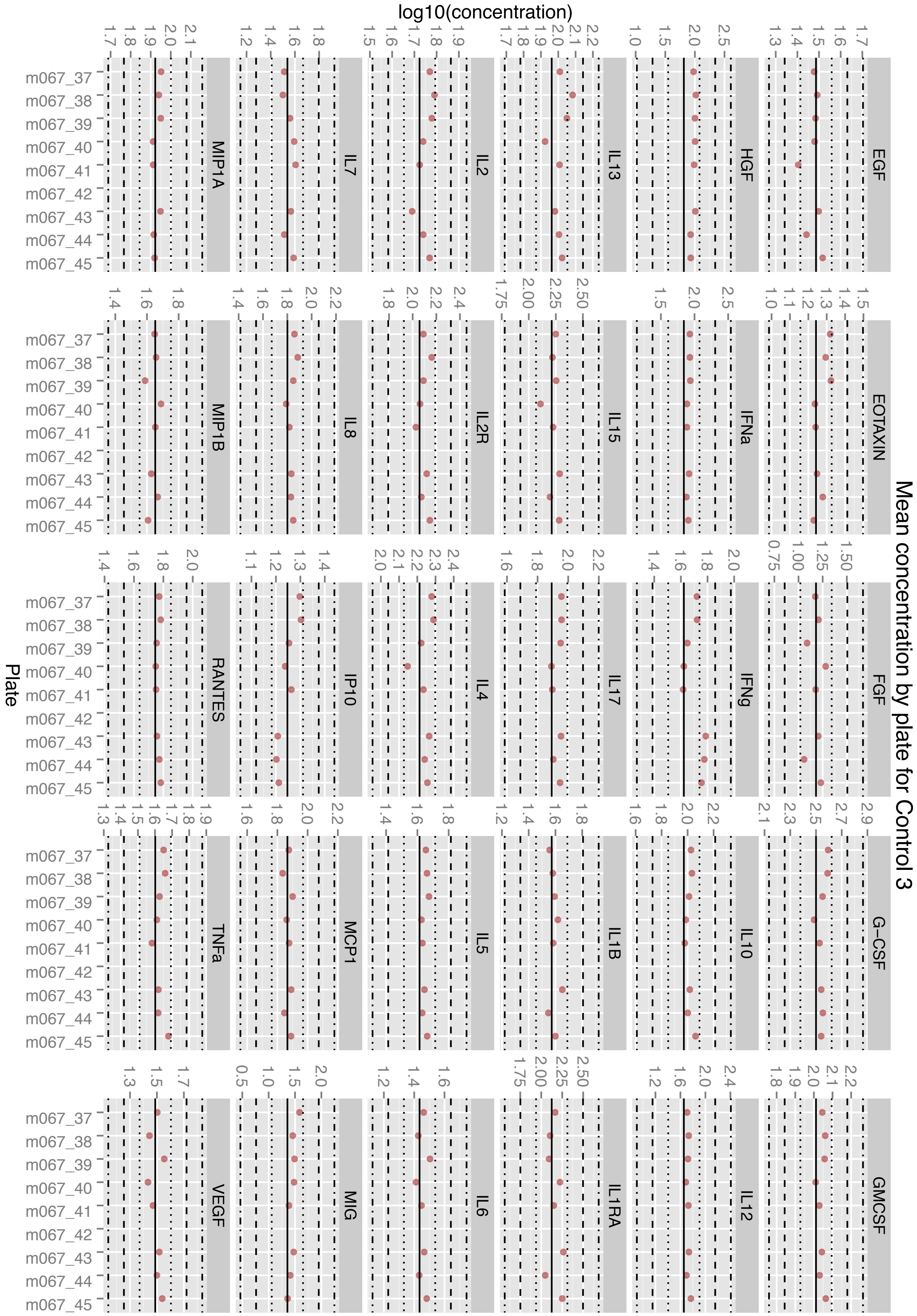
Mean concentration by plate for Control 3
log10(concentration)
Plate
EGF
HGF
IL13
IL2
IL7
MIP1A
EOTAXIN
IFNa
IL15
IL2R
IL8
MIP1B
FGF
IFNg
IL17
IL4
IP10
RANTES
G−CSF
IL10
IL1B
IL5
MCP1
TNFa
GMCSF
IL12
IL1RA
IL6
MIG
VEGF
m067_37
m067_38
m067_39
m067_40
m067_41
m067_42
m067_43
m067_44
m067_45

7.2 Coefficient of variation by day

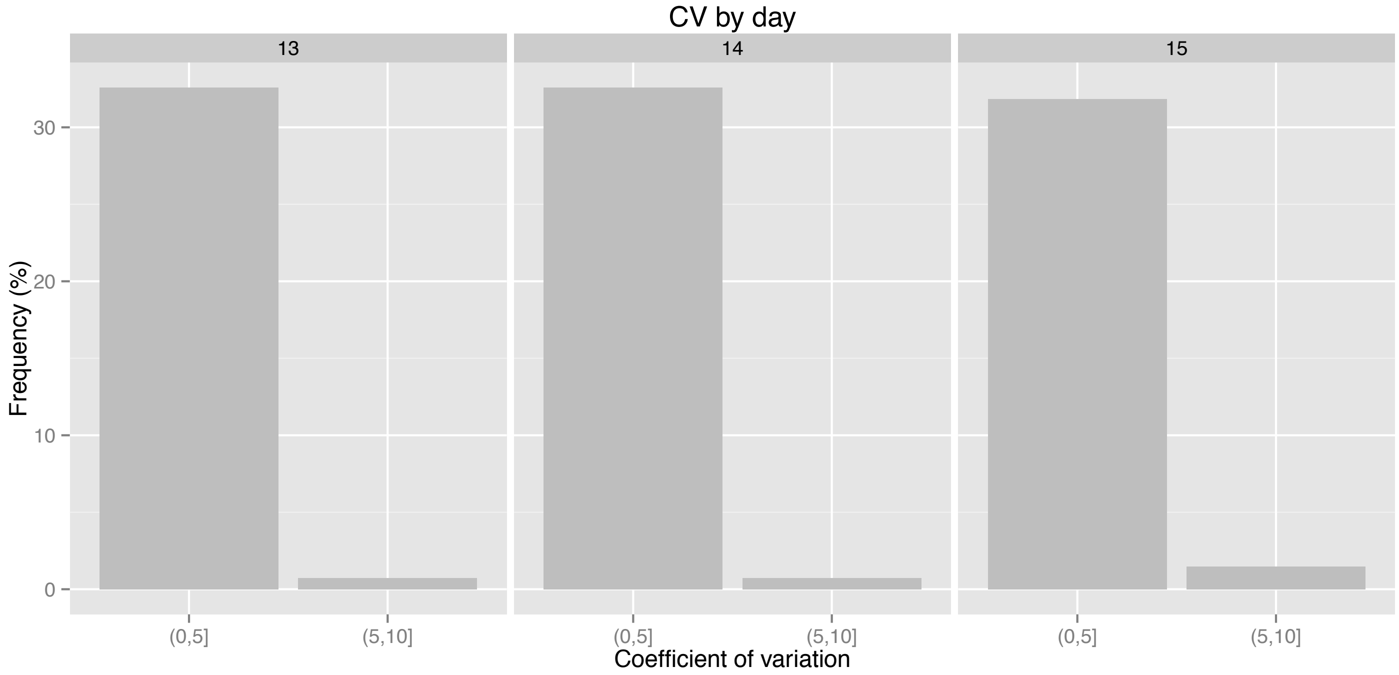

**Day**	**CV categories**					
	(0,5]	(5,10]	(10,15]	(15,20]	(20,30]	(30,Inf]
13	88	2	0	0	0	0
14	88	2	0	0	0	0
15	86	4	0	0	0	0

7.5 Agreement for positive controls

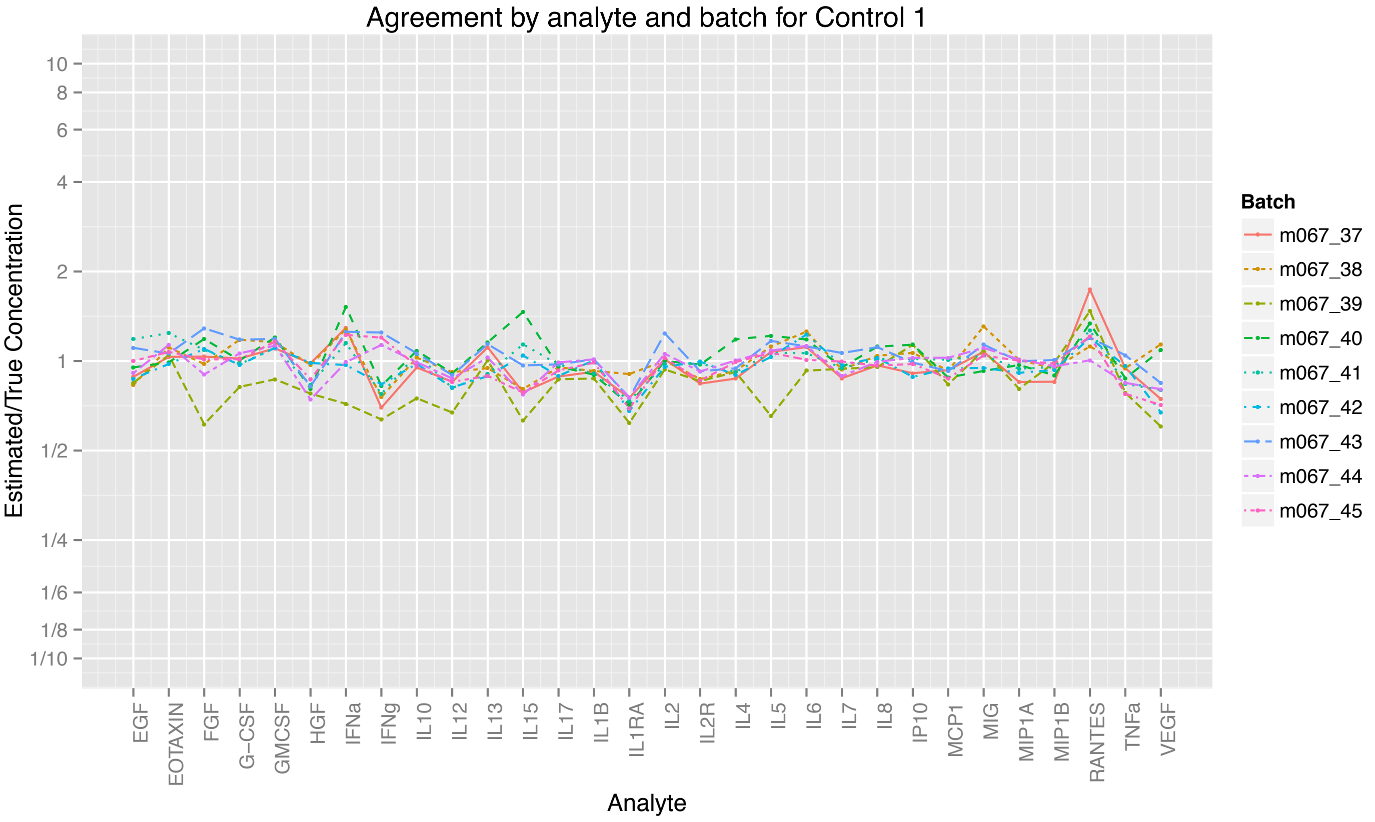

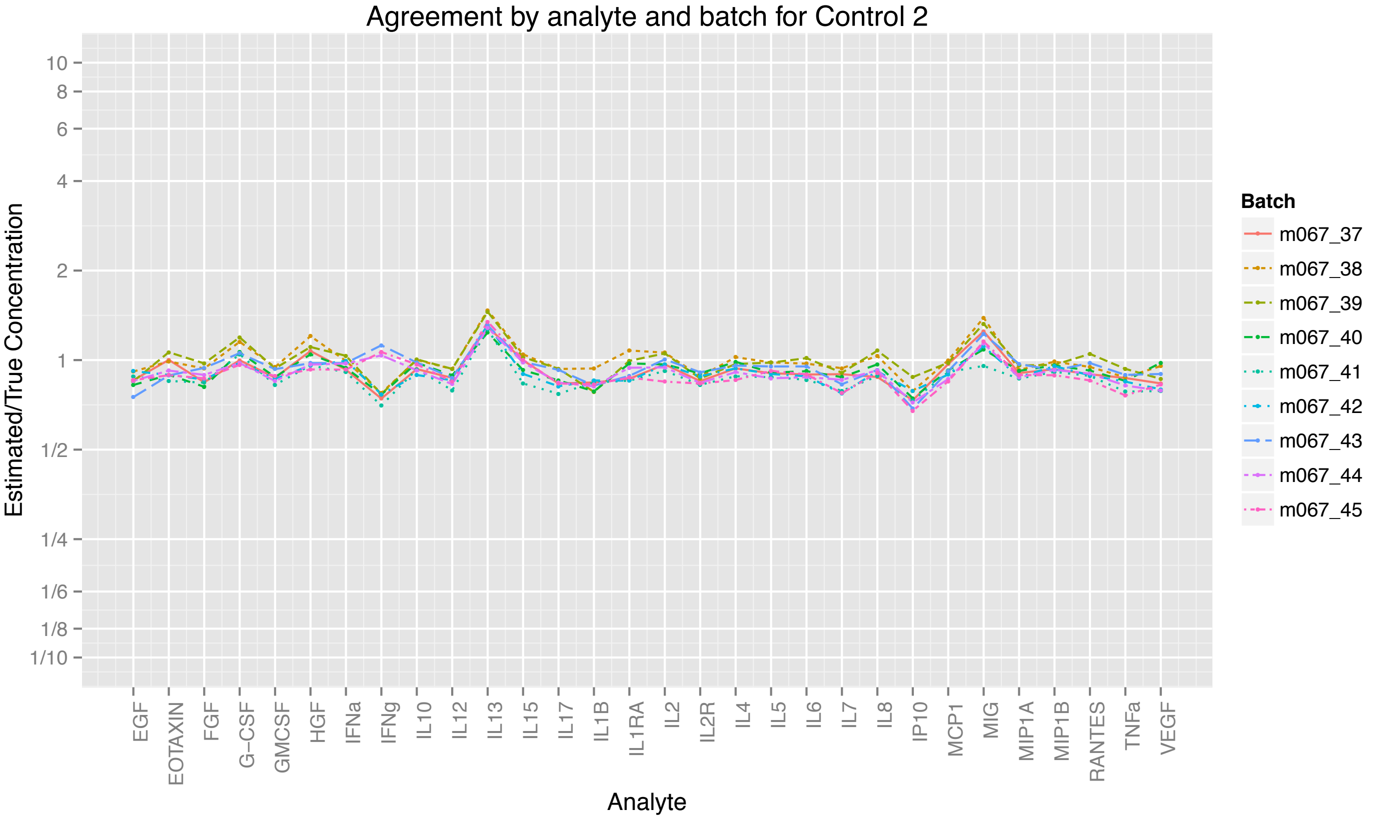

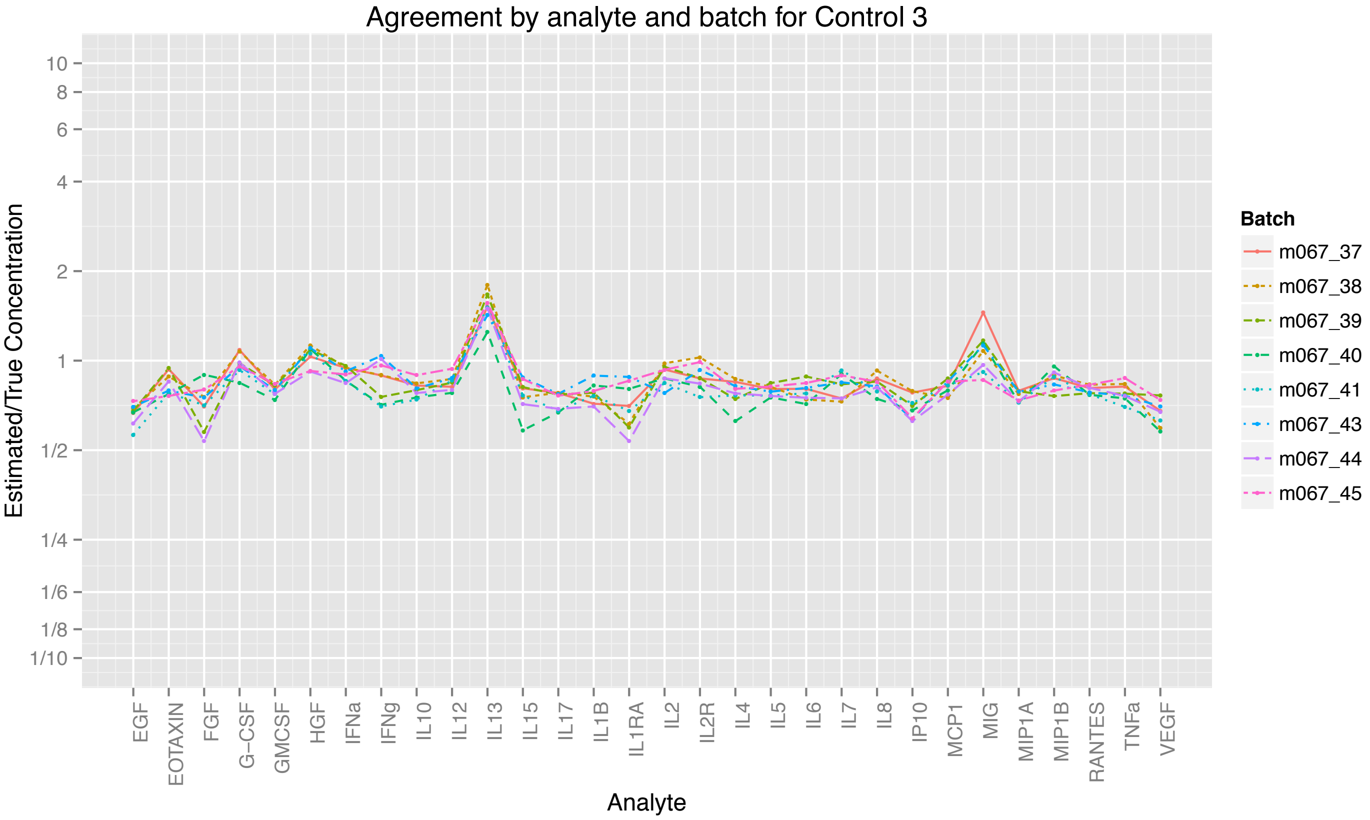
Agreement by analyte and batch for Control 3
Estimated/True Concentration
10
8
6
4
2
1
1/2
1/4
1/6
1/8
1/10
EGF
EOTAXIN
FGF
G−CSF
GMCSF
HGF
IFNa
IFNg
IL10
IL12
IL13
IL15
IL17
IL1B
IL1RA
IL2
IL2R
IL4
IL5
IL6
IL7
IL8
IP10
MCP1
MIG
MIP1A
MIP1B
RANTES
TNFa
VEGF
Analyte
Batch
m067_37
m067_38
m067_39
m067_40
m067_41
m067_43
m067_44
m067_45

8.6 Standard curve and Residuals plots by analyte and stimulation

Standard curve plot information:

- Orange point: positive control.
- Black filled point: standard curve value.
- Green line: background.
- Non-filled black point: values from standard samples not included in the estimation of the curve (flagged).

Residuals plot information:

- Black line: 0 value reference.
- Dashed line: ± 2.5 value (outlier limit).
- Black filled point: standardized residual value of the fitted point.

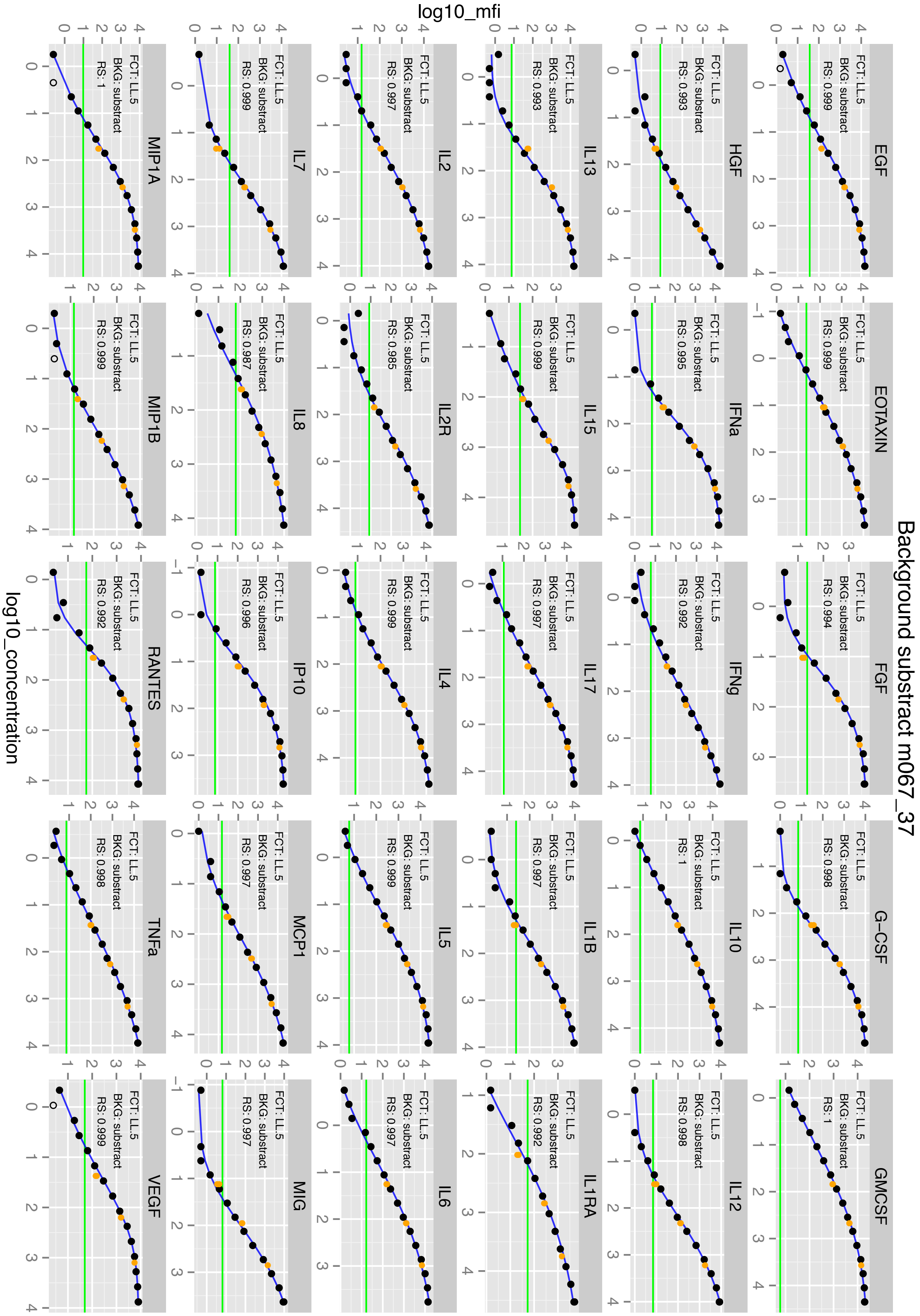
Background substract m067_37
log10_mfi
log10_concentration
EGF
EOTAXIN
FGF
G−CSF
GMCSF
HGF
IFNa
IFNg
IL10
IL12
IL13
IL15
IL17
IL1B
IL1RA
IL2
IL2R
IL4
IL5
IL6
IL7
IL8
IP10
MCP1
MIG
MIP1A
MIP1B
RANTES
TNFa
VEGF

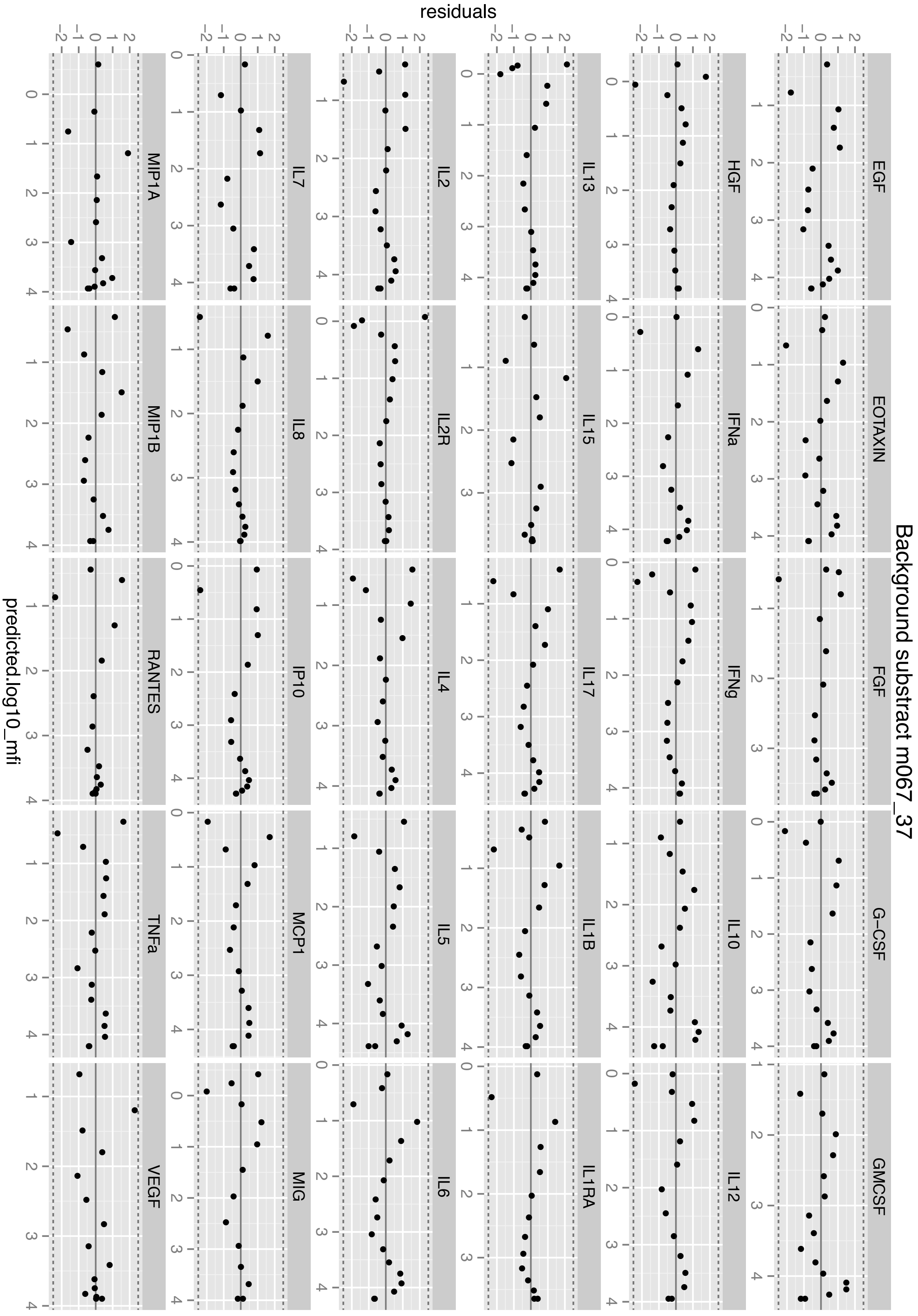
Background substract m067_37
residuals
predicted.log10_mfi
EGF
EOTAXIN
FGF
G−CSF
GMCSF
HGF
IFNa
IFNg
IL10
IL12
IL13
IL15
IL17
IL1B
IL1RA
IL2
IL2R
IL4
IL5
IL6
IL7
IL8
IP10
MCP1
MIG
MIP1A
MIP1B
RANTES
TNFa
VEGF

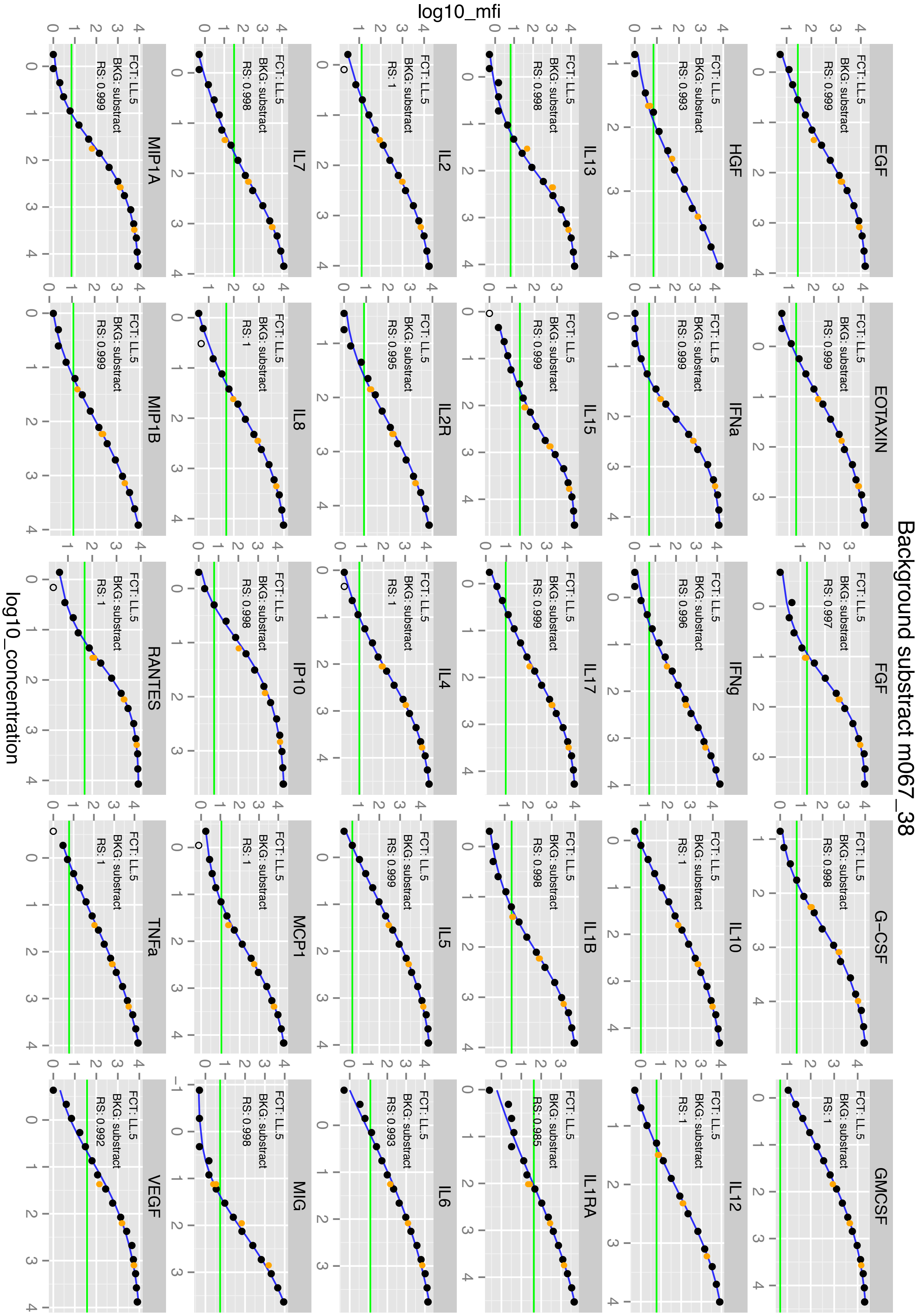
Background substract m067_38
log10_mfi
log10_concentration
EGF
EOTAXIN
FGF
G−CSF
GMCSF
HGF
IFNa
IFNg
IL10
IL12
IL13
IL15
IL17
IL1B
IL1RA
IL2
IL2R
IL4
IL5
IL6
IL7
IL8
IP10
MCP1
MIG
MIP1A
MIP1B
RANTES
TNFa
VEGF
FCT: LL.5
BKG: substract

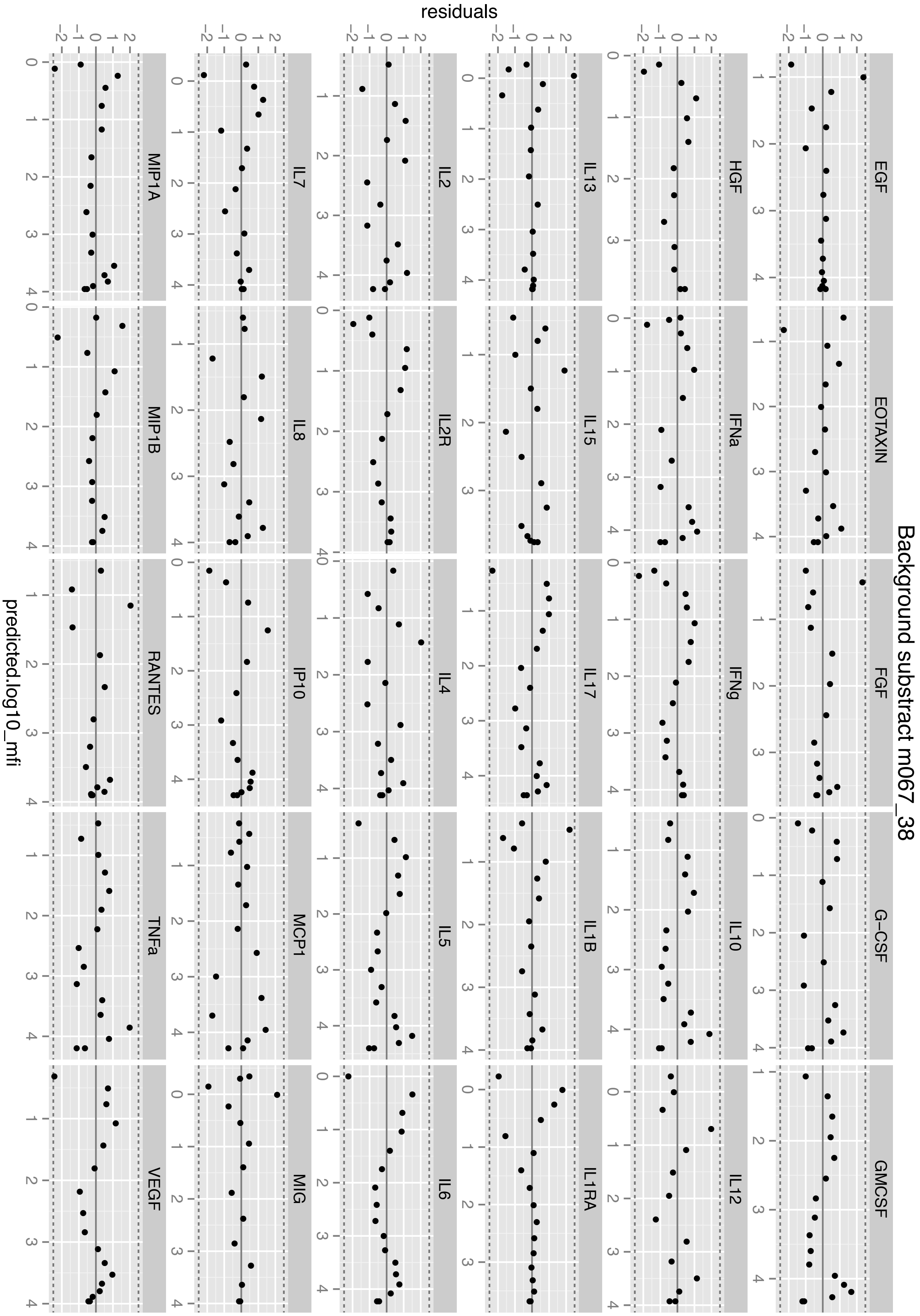
Background substract m067_38
residuals
predicted.log10_mfi
EGF
EOTAXIN
FGF
G−CSF
GMCSF
HGF
IFNa
IFNg
IL10
IL12
IL13
IL15
IL17
IL1B
IL1RA
IL2
IL2R
IL4
IL5
IL6
IL7
IL8
IP10
MCP1
MIG
MIP1A
MIP1B
RANTES
TNFa
VEGF

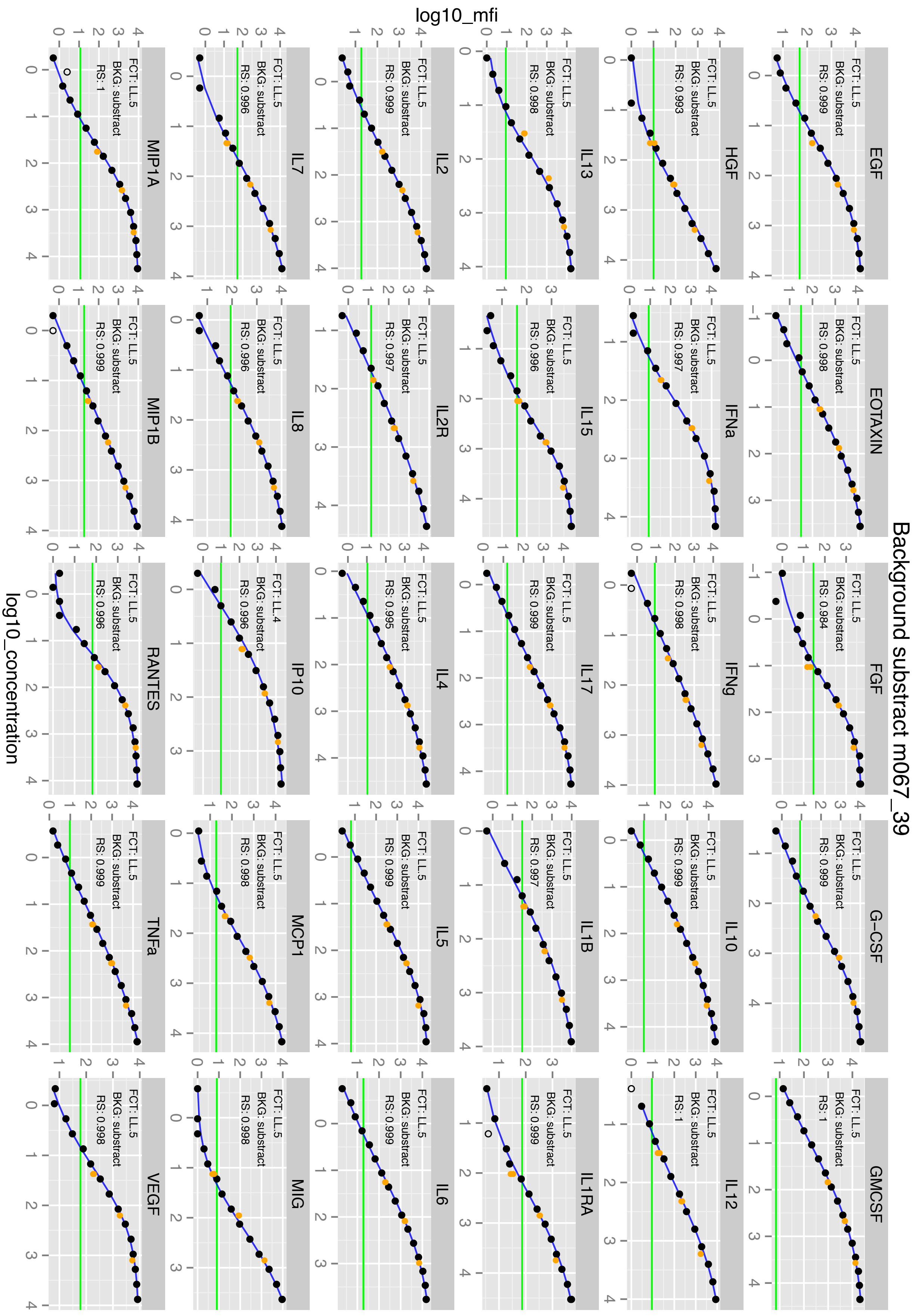
Background substract m067_39
log10_mfi
log10_concentration
EGF FCT: LL.5 BKG: substract RS: 0.999
EOTAXIN FCT: LL.5 BKG: substract RS: 0.998
FGF FCT: LL.5 BKG: substract RS: 0.984
G−CSF FCT: LL.5 BKG: substract RS: 0.999
GMCSF FCT: LL.5 BKG: substract RS: 1
HGF FCT: LL.5 BKG: substract RS: 0.993
IFNa FCT: LL.5 BKG: substract RS: 0.997
IFNg FCT: LL.5 BKG: substract RS: 0.998
IL10 FCT: LL.5 BKG: substract RS: 0.999
IL12 FCT: LL.5 BKG: substract RS: 1
IL13 FCT: LL.5 BKG: substract RS: 0.998
IL15 FCT: LL.5 BKG: substract RS: 0.996
IL17 FCT: LL.5 BKG: substract RS: 0.999
IL1B FCT: LL.5 BKG: substract RS: 0.997
IL1RA FCT: LL.5 BKG: substract RS: 0.999
IL2 FCT: LL.5 BKG: substract RS: 0.999
IL2R FCT: LL.5 BKG: substract RS: 0.997
IL4 FCT: LL.5 BKG: substract RS: 0.995
IL5 FCT: LL.5 BKG: substract RS: 0.999
IL6 FCT: LL.5 BKG: substract RS: 0.999
IL7 FCT: LL.5 BKG: substract RS: 0.996
IL8 FCT: LL.5 BKG: substract RS: 0.996
IP10 FCT: LL.4 BKG: substract RS: 0.996
MCP1 FCT: LL.5 BKG: substract RS: 0.998
MIG FCT: LL.5 BKG: substract RS: 0.998
MIP1A FCT: LL.5 BKG: substract RS: 1
MIP1B FCT: LL.5 BKG: substract RS: 0.999
RANTES FCT: LL.5 BKG: substract RS: 0.996
TNFa FCT: LL.5 BKG: substract RS: 0.999
VEGF FCT: LL.5 BKG: substract RS: 0.998

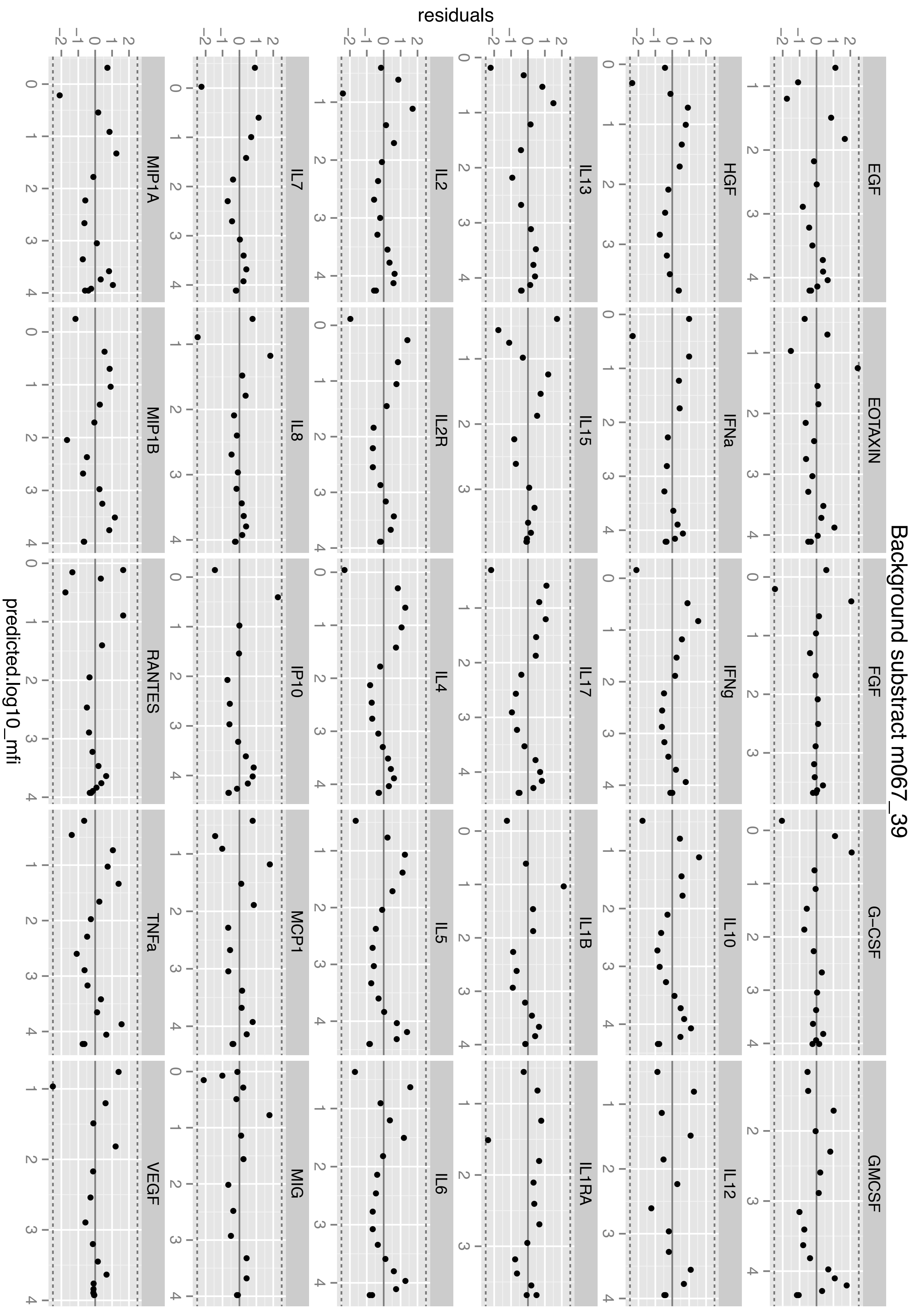
Background substract m067_39
residuals
predicted.log10_mfi
EGF
EOTAXIN
FGF
G−CSF
GMCSF
HGF
IFNa
IFNg
IL10
IL12
IL13
IL15
IL17
IL1B
IL1RA
IL2
IL2R
IL4
IL5
IL6
IL7
IL8
IP10
MCP1
MIG
MIP1A
MIP1B
RANTES
TNFa
VEGF

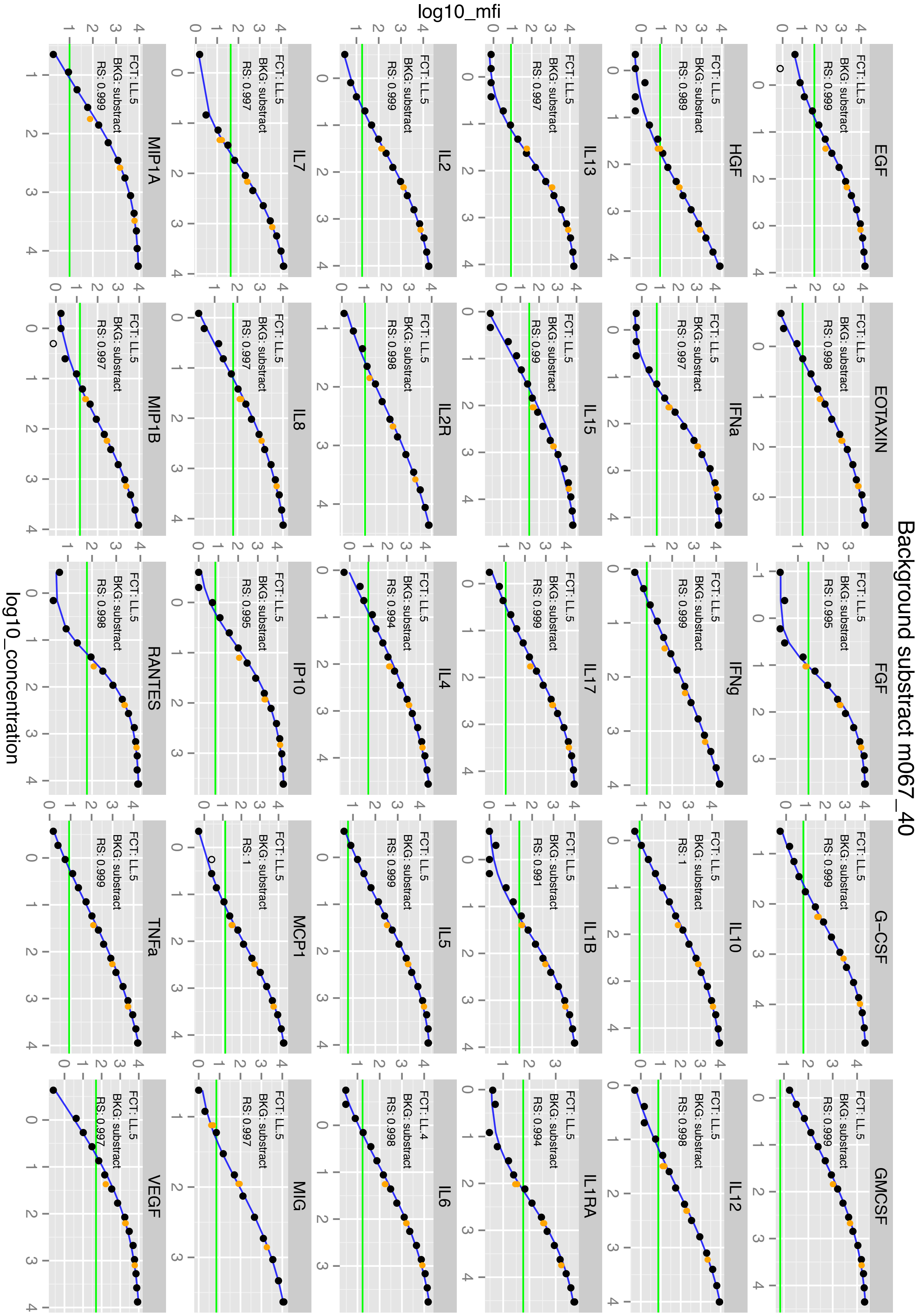

Background substract m067_40
log10_mfi
log10_concentration
EGF FCT: LL.5 BKG: substract RS: 0.999
EOTAXIN FCT: LL.5 BKG: substract RS: 0.998
FGF FCT: LL.5 BKG: substract RS: 0.995
G−CSF FCT: LL.5 BKG: substract RS: 0.999
GMCSF FCT: LL.5 BKG: substract RS: 0.999
HGF FCT: LL.5 BKG: substract RS: 0.989
IFNa FCT: LL.5 BKG: substract RS: 0.997
IFNg FCT: LL.5 BKG: substract RS: 0.999
IL10 FCT: LL.5 BKG: substract RS: 1
IL12 FCT: LL.5 BKG: substract RS: 0.998
IL13 FCT: LL.5 BKG: substract RS: 0.997
IL15 FCT: LL.5 BKG: substract RS: 0.99
IL17 FCT: LL.5 BKG: substract RS: 0.999
IL1B FCT: LL.5 BKG: substract RS: 0.991
IL1RA FCT: LL.5 BKG: substract RS: 0.994
IL2 FCT: LL.5 BKG: substract RS: 0.999
IL2R FCT: LL.5 BKG: substract RS: 0.998
IL4 FCT: LL.5 BKG: substract RS: 0.994
IL5 FCT: LL.5 BKG: substract RS: 0.999
IL6 FCT: LL.4 BKG: substract RS: 0.998
IL7 FCT: LL.5 BKG: substract RS: 0.997
IL8 FCT: LL.5 BKG: substract RS: 0.997
IP10 FCT: LL.5 BKG: substract RS: 0.995
MCP1 FCT: LL.5 BKG: substract RS: 1
MIG FCT: LL.5 BKG: substract RS: 0.997
MIP1A FCT: LL.5 BKG: substract RS: 0.999
MIP1B FCT: LL.5 BKG: substract RS: 0.997
RANTES FCT: LL.5 BKG: substract RS: 0.998
TNFa FCT: LL.5 BKG: substract RS: 0.999
VEGF FCT: LL.5 BKG: substract RS: 0.997

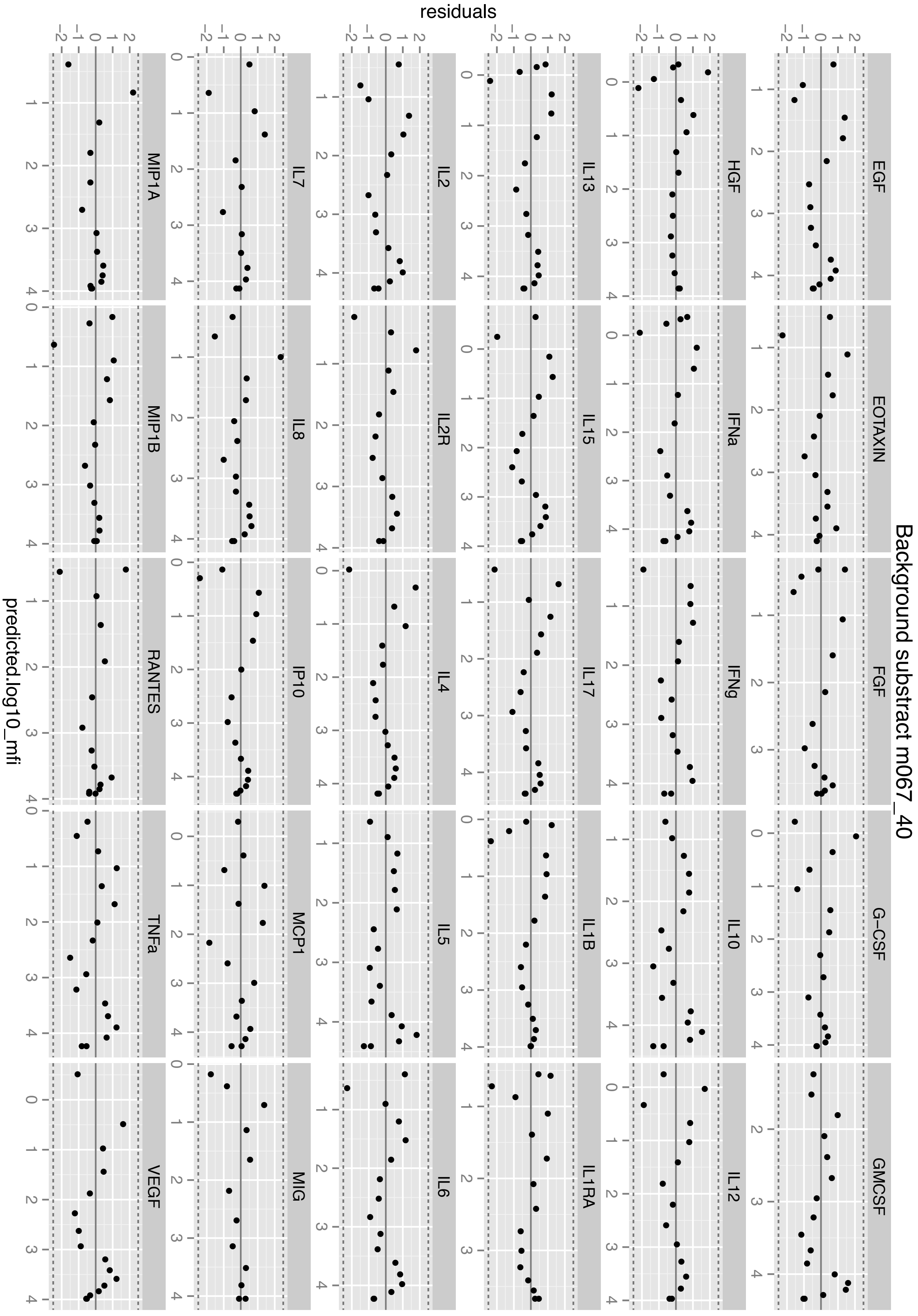

Background substract m067_40
residuals
predicted.log10_mfi
EGF
EOTAXIN
FGF
G−CSF
GMCSF
HGF
IFNa
IFNg
IL10
IL12
IL13
IL15
IL17
IL1B
IL1RA
IL2
IL2R
IL4
IL5
IL6
IL7
IL8
IP10
MCP1
MIG
MIP1A
MIP1B
RANTES
TNFa
VEGF

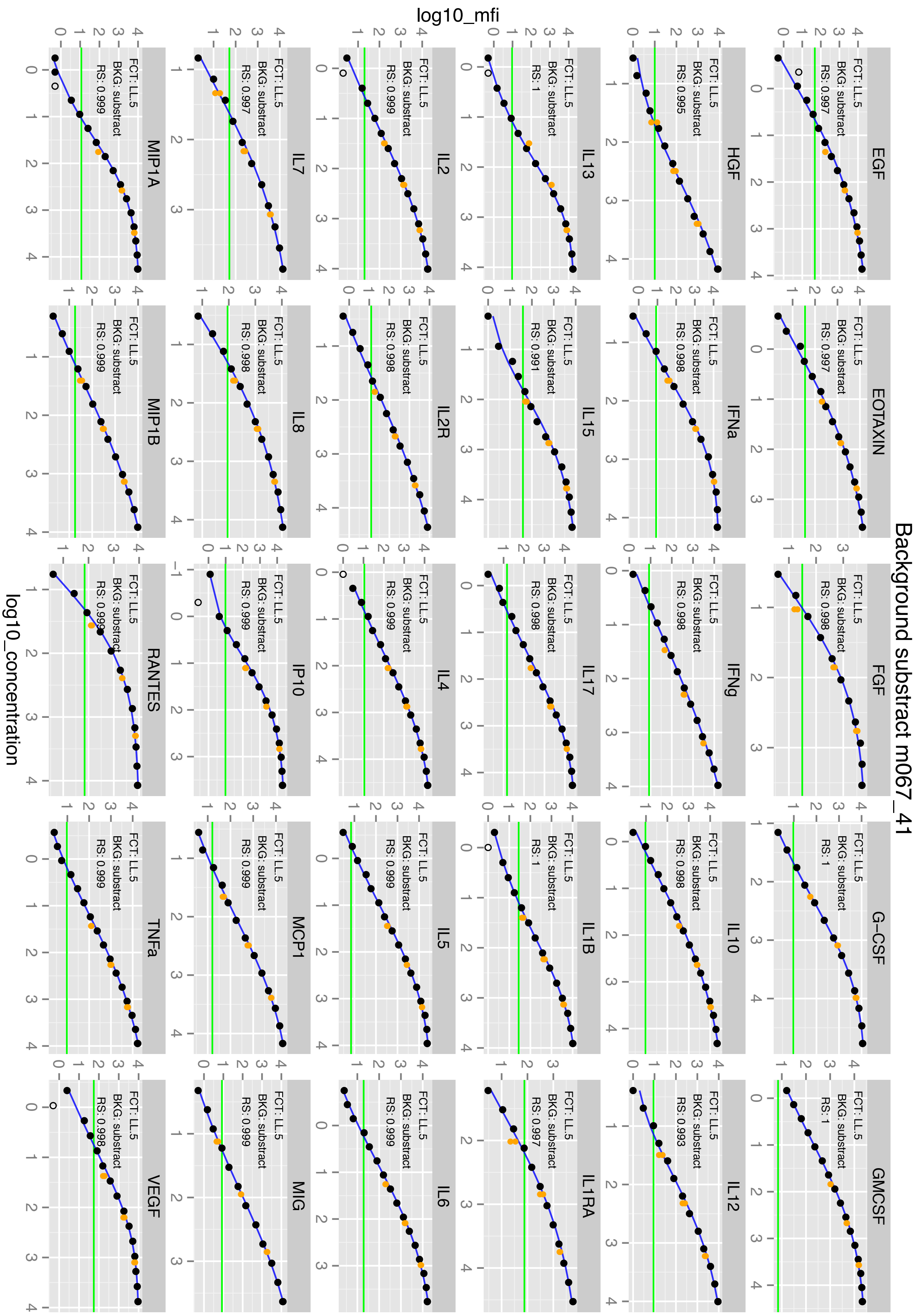
Background substract m067_41
log10_mfi
log10_concentration
EGF
EOTAXIN
FGF
G−CSF
GMCSF
HGF
IFNa
IFNg
IL10
IL12
IL13
IL15
IL17
IL1B
IL1RA
IL2
IL2R
IL4
IL5
IL6
IL7
IL8
IP10
MCP1
MIG
MIP1A
MIP1B
RANTES
TNFa
VEGF

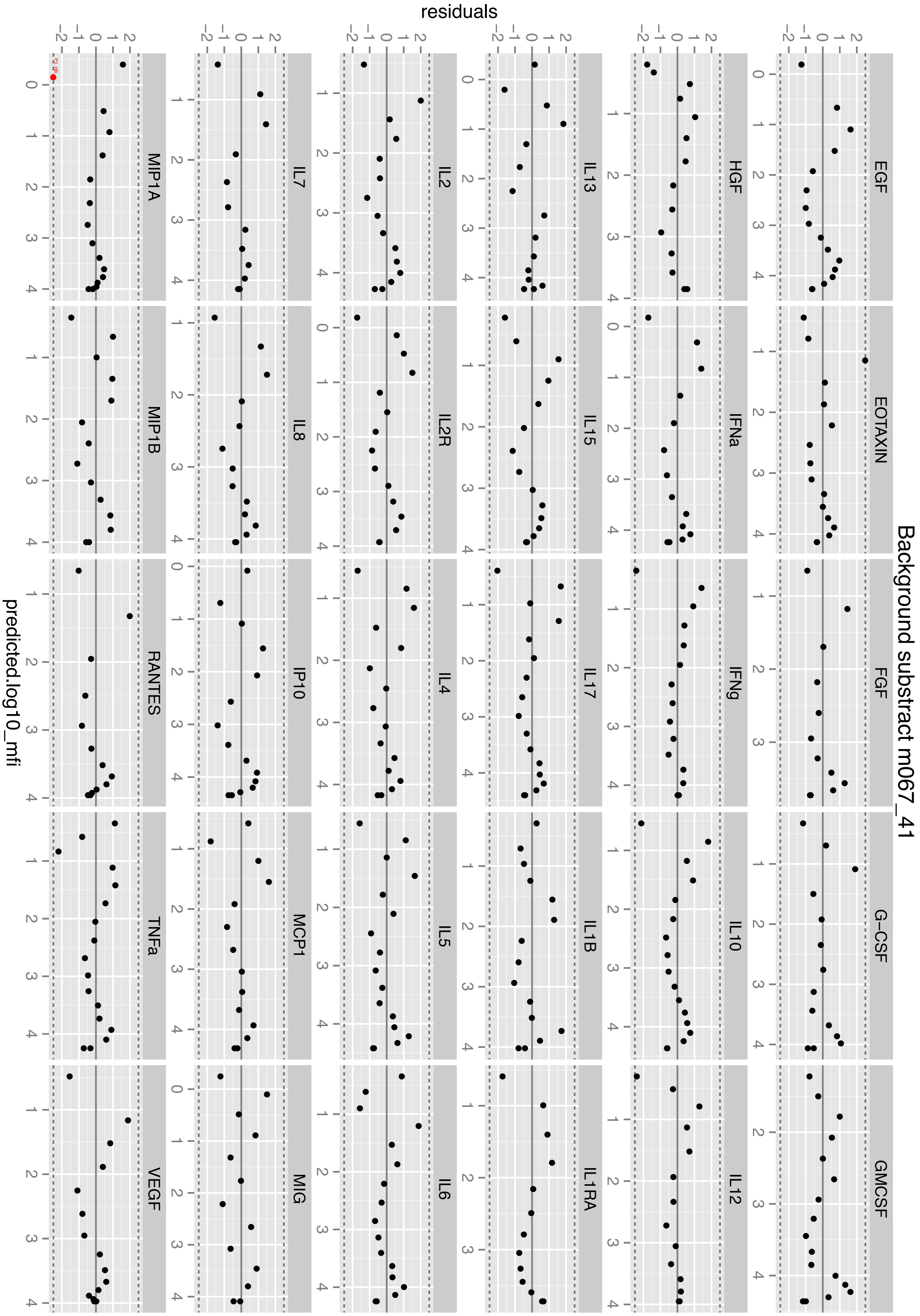
Background substract m067_41
residuals
predicted.log10_mfi
EGF
EOTAXIN
FGF
G−CSF
GMCSF
HGF
IFNa
IFNg
IL10
IL12
IL13
IL15
IL17
IL1B
IL1RA
IL2
IL2R
IL4
IL5
IL6
IL7
IL8
IP10
MCP1
MIG
MIP1A
MIP1B
RANTES
TNFa
VEGF

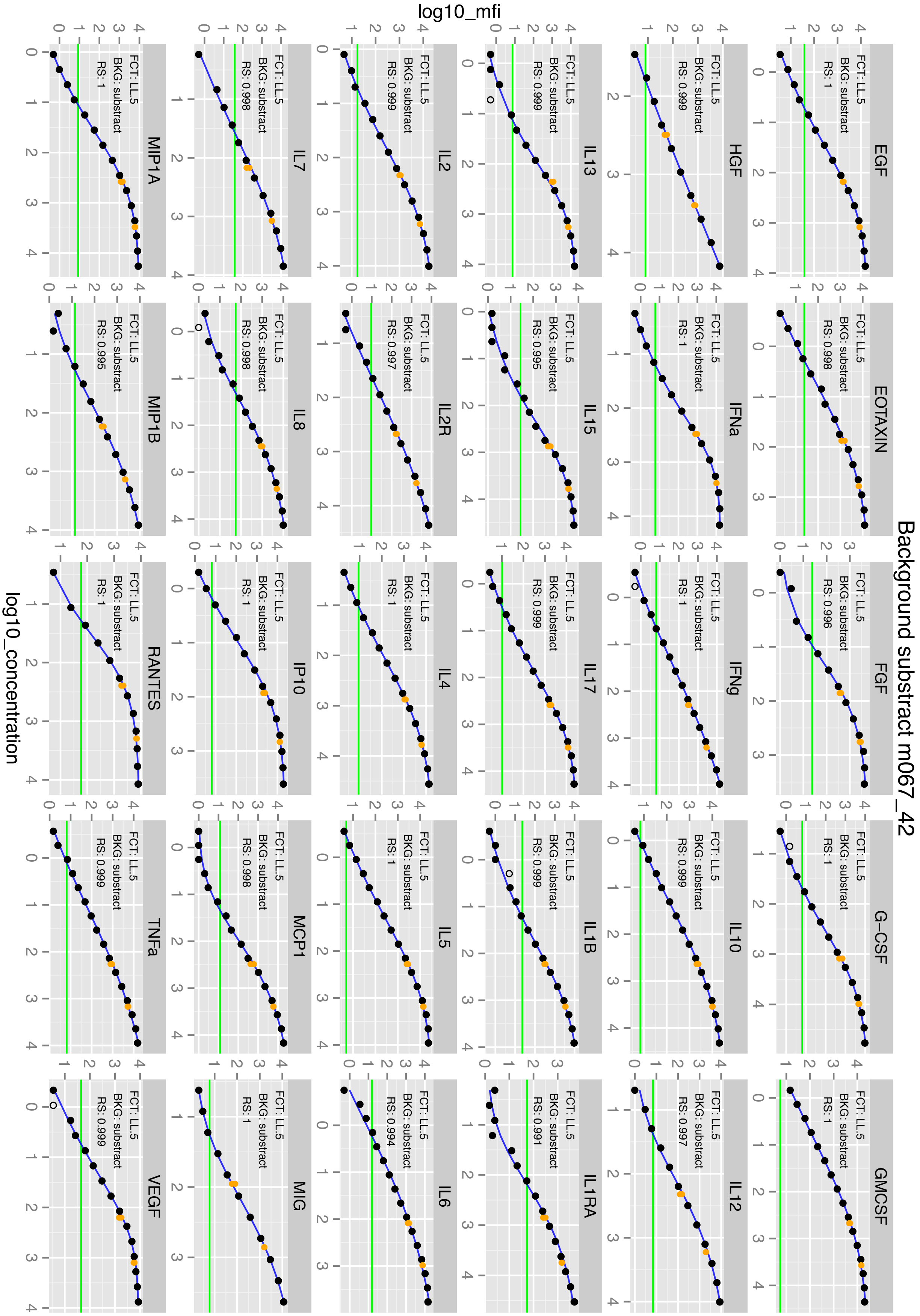
Background substract m067_42
log10_mfi
log10_concentration
EGF
EOTAXIN
FGF
G−CSF
GMCSF
HGF
IFNa
IFNg
IL10
IL12
IL13
IL15
IL17
IL1B
IL1RA
IL2
IL2R
IL4
IL5
IL6
IL7
IL8
IP10
MCP1
MIG
MIP1A
MIP1B
RANTES
TNFa
VEGF

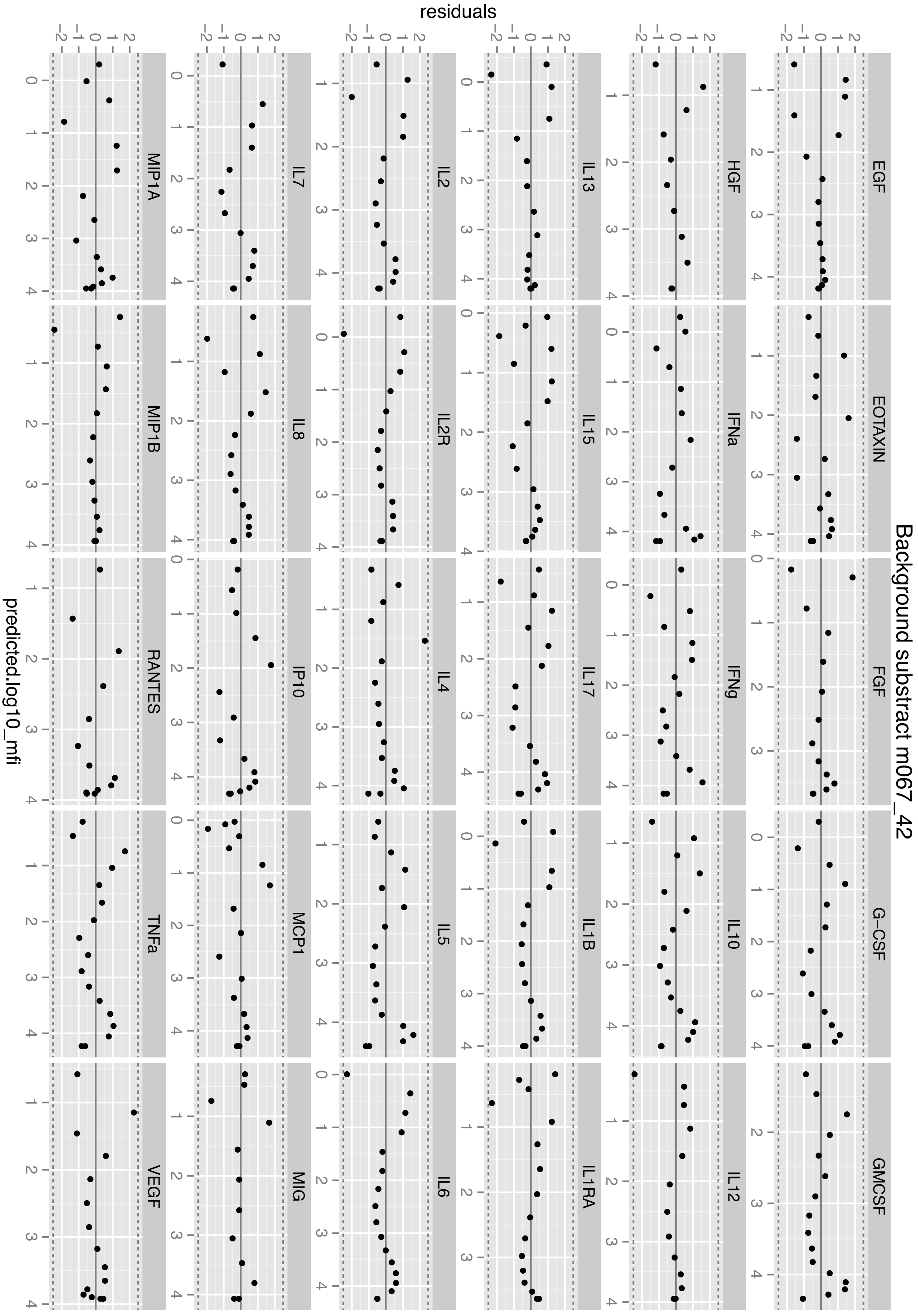
Background substract m067_42
residuals
predicted.log10_mfi
EGF
EOTAXIN
FGF
G−CSF
GMCSF
HGF
IFNa
IFNg
IL10
IL12
IL13
IL15
IL17
IL1B
IL1RA
IL2
IL2R
IL4
IL5
IL6
IL7
IL8
IP10
MCP1
MIG
MIP1A
MIP1B
RANTES
TNFa
VEGF

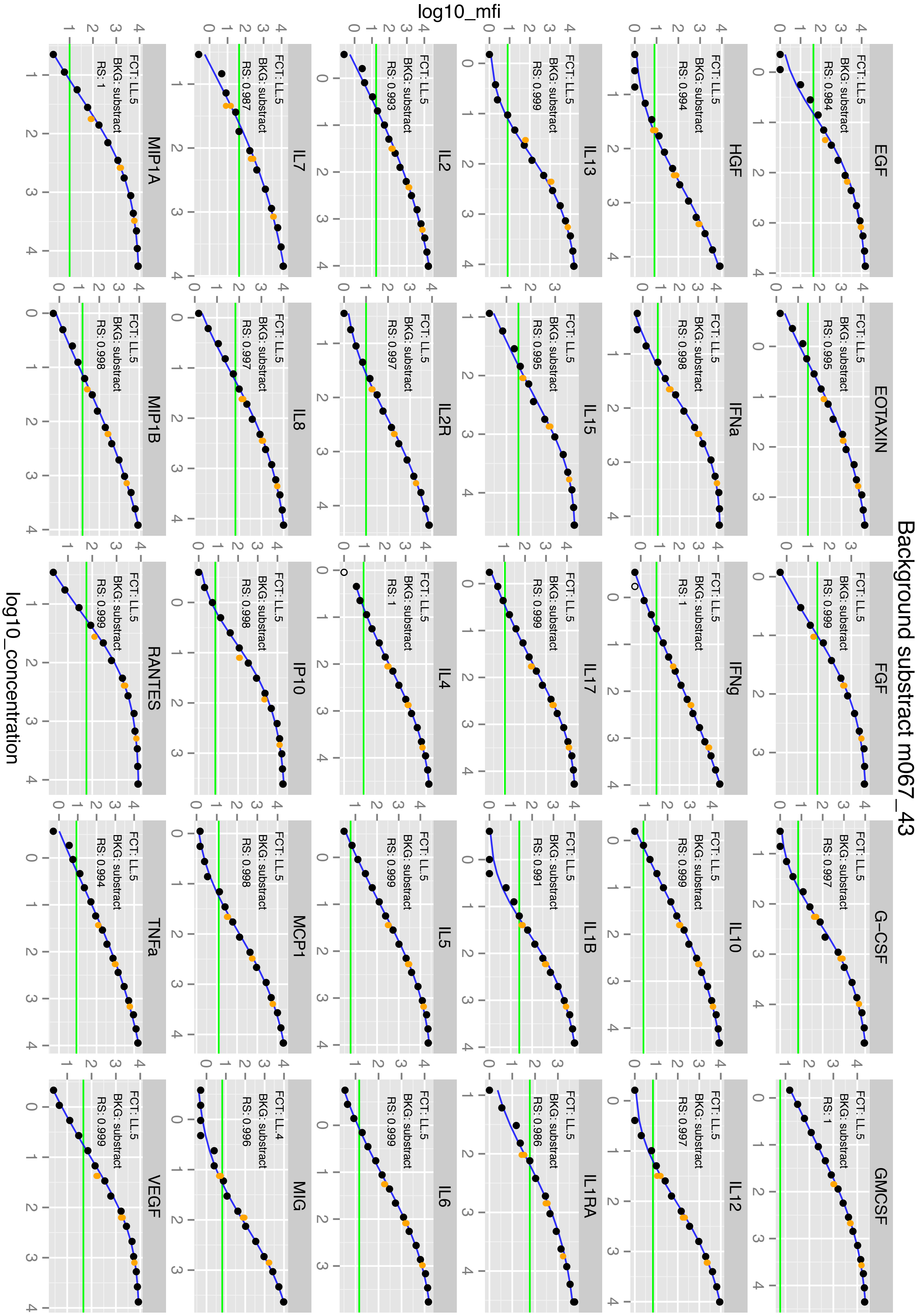
Background substract m067_43
log10_mfi
log10_concentration
EGF
EOTAXIN
FGF
G−CSF
GMCSF
HGF
IFNa
IFNg
IL10
IL12
IL13
IL15
IL17
IL1B
IL1RA
IL2
IL2R
IL4
IL5
IL6
IL7
IL8
IP10
MCP1
MIG
MIP1A
MIP1B
RANTES
TNFa
VEGF

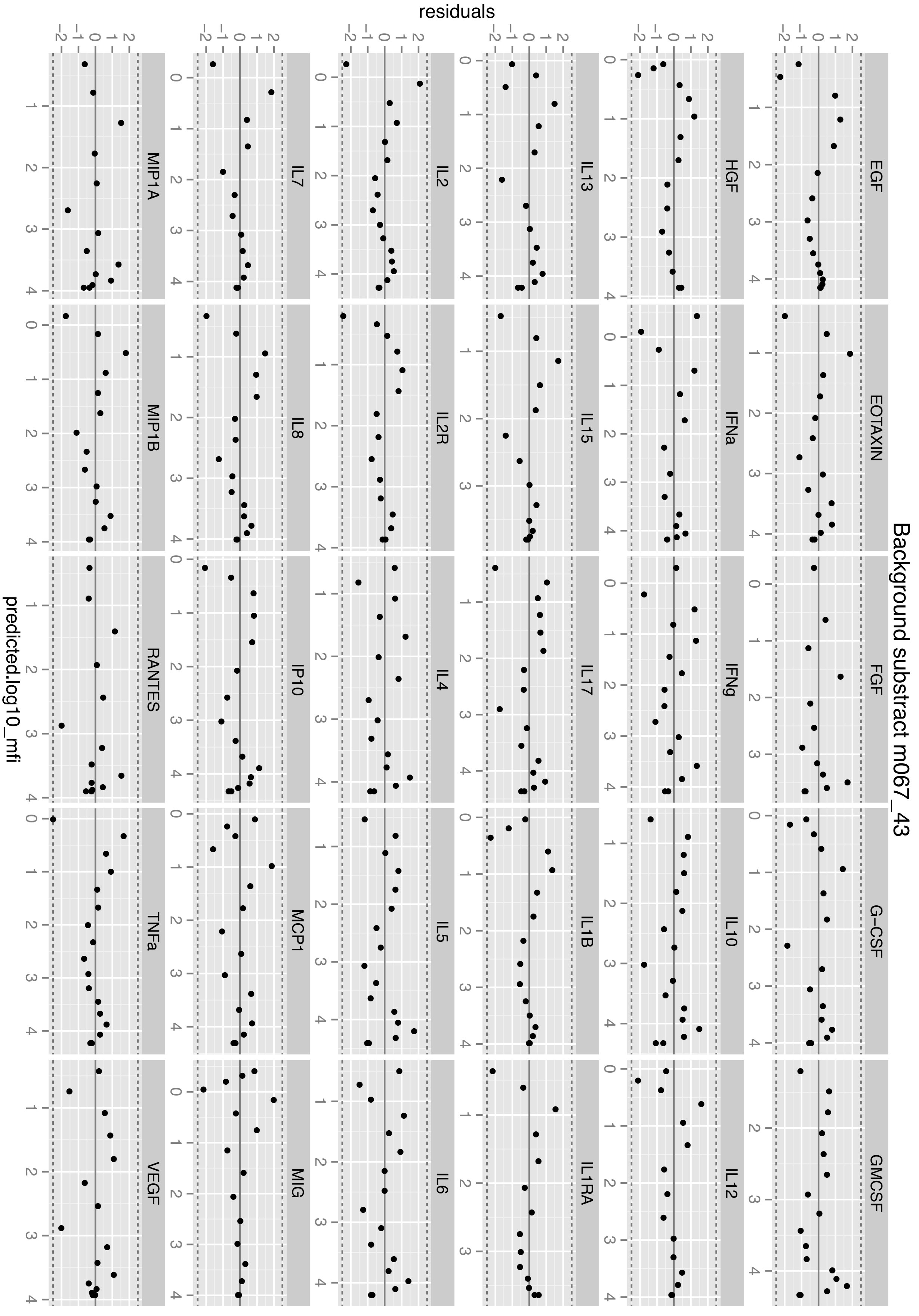
Background substract m067_43
residuals
predicted.log10_mfi
EGF
EOTAXIN
FGF
G−CSF
GMCSF
HGF
IFNa
IFNg
IL10
IL12
IL13
IL15
IL17
IL1B
IL1RA
IL2
IL2R
IL4
IL5
IL6
IL7
IL8
IP10
MCP1
MIG
MIP1A
MIP1B
RANTES
TNFa
VEGF

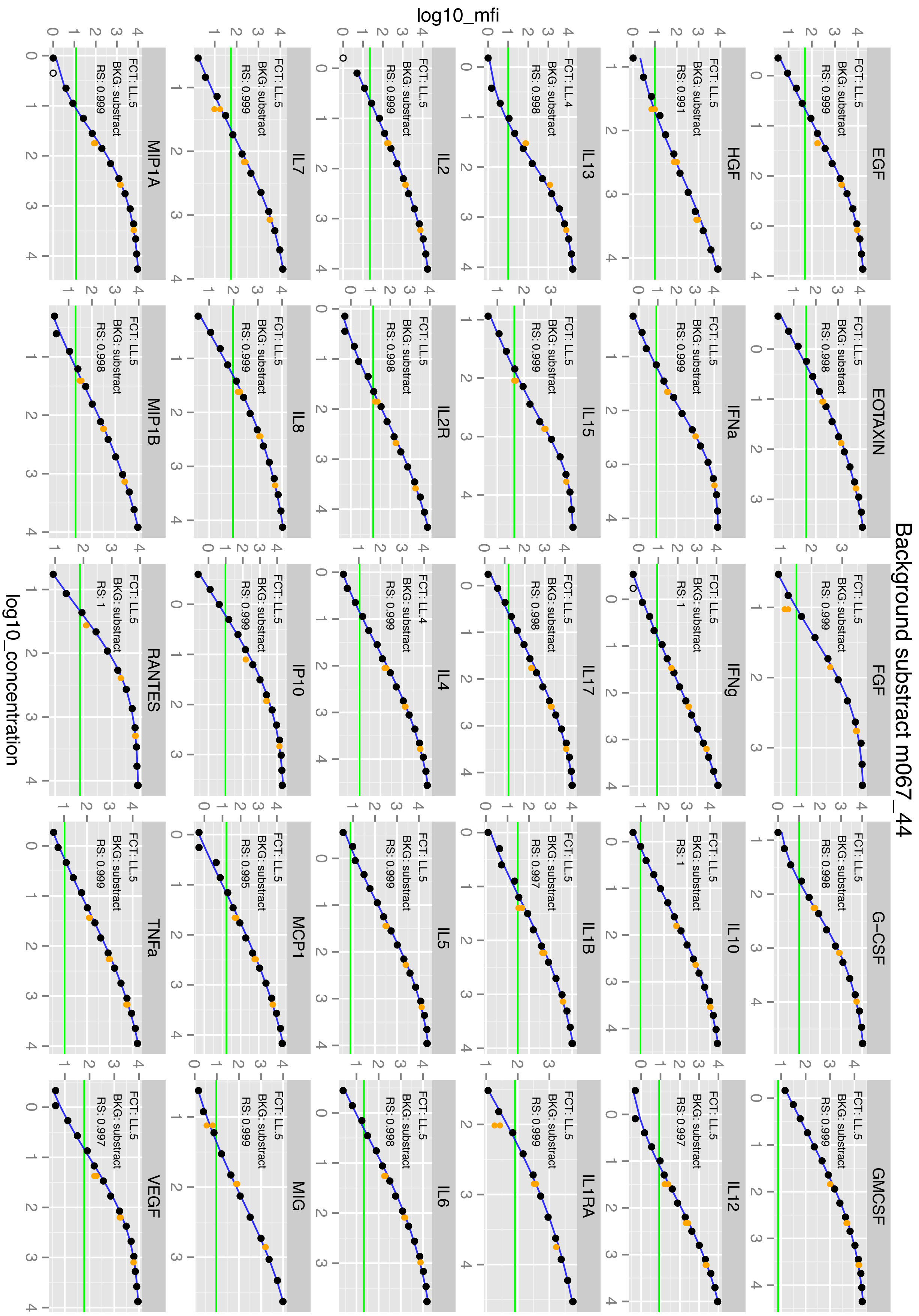
Background substract m067_44
log10_mfi
log10_concentration
EGF
FCT: LL.5
BKG: substract
RS: 0.999
EOTAXIN
FCT: LL.5
BKG: substract
RS: 0.998
FGF
FCT: LL.5
BKG: substract
RS: 0.999
G−CSF
FCT: LL.5
BKG: substract
RS: 0.998
GMCSF
FCT: LL.5
BKG: substract
RS: 0.999
HGF
FCT: LL.5
BKG: substract
RS: 0.991
IFNa
FCT: LL.5
BKG: substract
RS: 0.999
IFNg
FCT: LL.5
BKG: substract
RS: 1
IL10
FCT: LL.5
BKG: substract
RS: 1
IL12
FCT: LL.5
BKG: substract
RS: 0.997
IL13
FCT: LL.4
BKG: substract
RS: 0.998
IL15
FCT: LL.5
BKG: substract
RS: 0.999
IL17
FCT: LL.5
BKG: substract
RS: 0.998
IL1B
FCT: LL.5
BKG: substract
RS: 0.997
IL1RA
FCT: LL.5
BKG: substract
RS: 0.999
IL2
FCT: LL.5
BKG: substract
RS: 0.999
IL2R
FCT: LL.5
BKG: substract
RS: 0.998
IL4
FCT: LL.4
BKG: substract
RS: 0.999
IL5
FCT: LL.5
BKG: substract
RS: 0.999
IL6
FCT: LL.5
BKG: substract
RS: 0.998
IL7
FCT: LL.5
BKG: substract
RS: 0.999
IL8
FCT: LL.5
BKG: substract
RS: 0.999
IP10
FCT: LL.5
BKG: substract
RS: 0.999
MCP1
FCT: LL.5
BKG: substract
RS: 0.995
MIG
FCT: LL.5
BKG: substract
RS: 0.999
MIP1A
FCT: LL.5
BKG: substract
RS: 0.999
MIP1B
FCT: LL.5
BKG: substract
RS: 0.998
RANTES
FCT: LL.5
BKG: substract
RS: 1
TNFa
FCT: LL.5
BKG: substract
RS: 0.999
VEGF
FCT: LL.5
BKG: substract
RS: 0.997

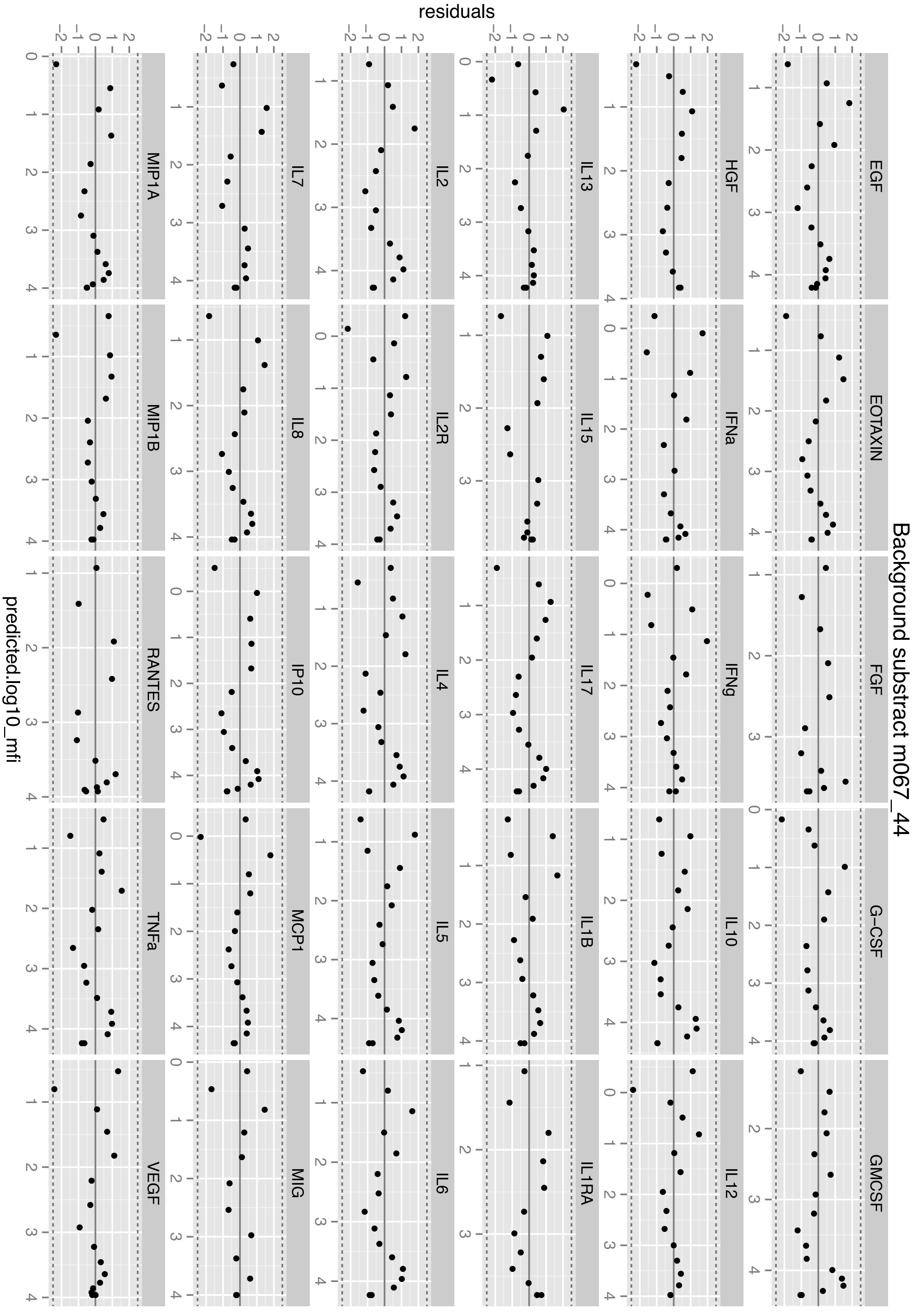

Background substract m067_44
residuals
predicted.log10_mfi
EGF
EOTAXIN
FGF
G−CSF
GMCSF
HGF
IFNa
IFNg
IL10
IL12
IL13
IL15
IL17
IL1B
IL1RA
IL2
IL2R
IL4
IL5
IL6
IL7
IL8
IP10
MCP1
MIG
MIP1A
MIP1B
RANTES
TNFa
VEGF

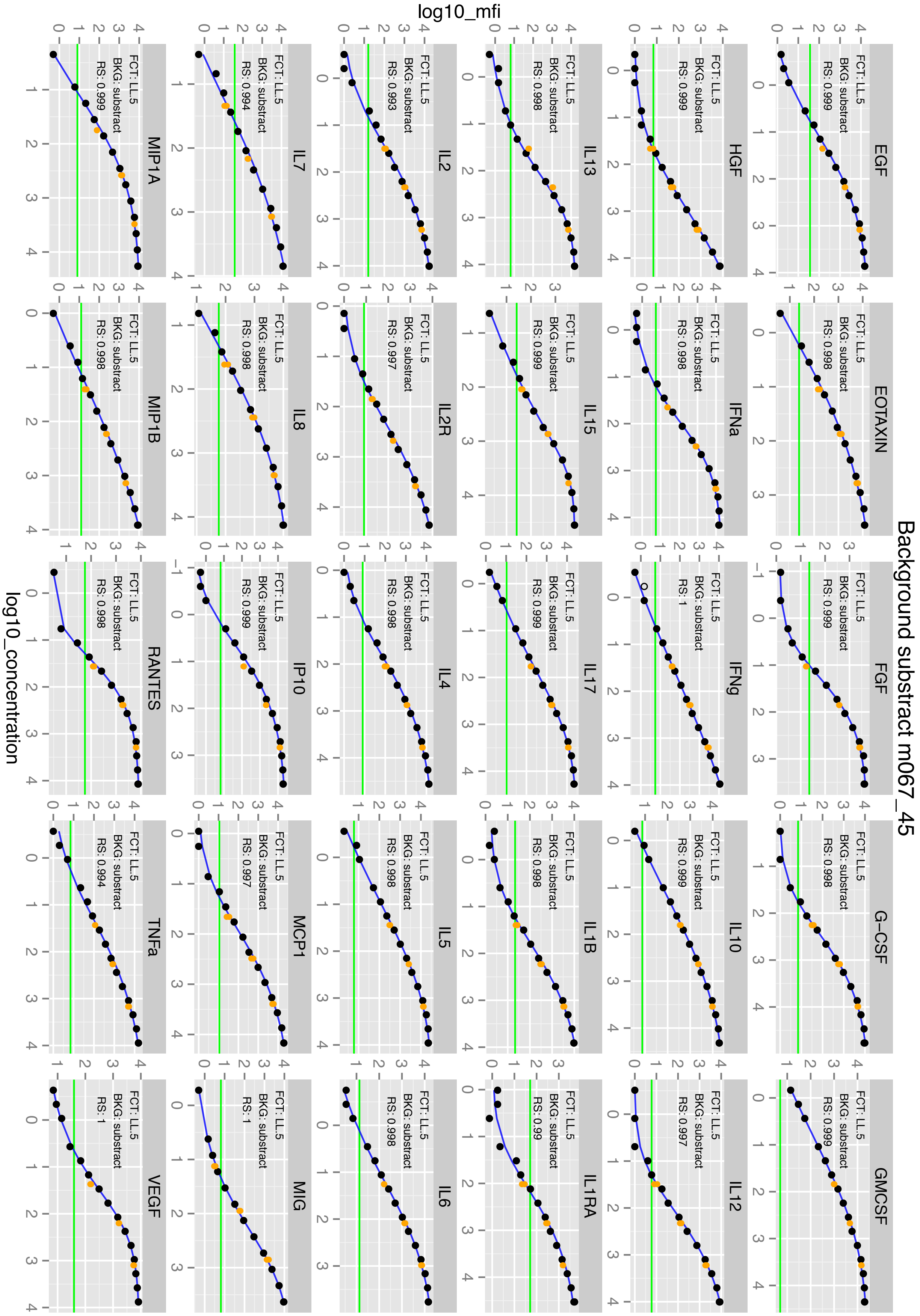
Background substract m067_45
log10_mfi
log10_concentration
EGF
EOTAXIN
FGF
G−CSF
GMCSF
HGF
IFNa
IFNg
IL10
IL12
IL13
IL15
IL17
IL1B
IL1RA
IL2
IL2R
IL4
IL5
IL6
IL7
IL8
IP10
MCP1
MIG
MIP1A
MIP1B
RANTES
TNFa
VEGF

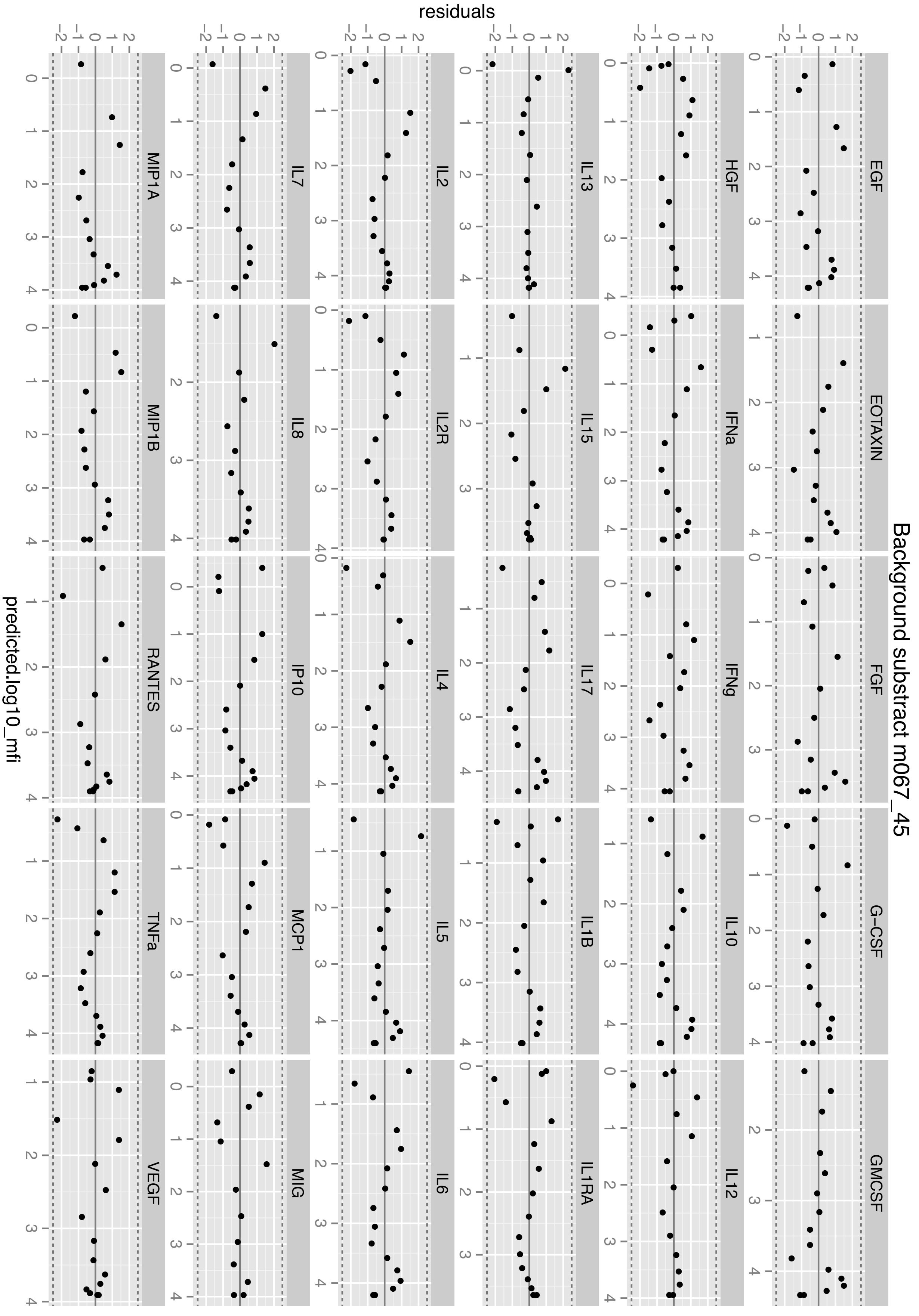
Background substract m067_45
residuals
predicted.log10_mfi
EGF
EOTAXIN
FGF
G−CSF
GMCSF
HGF
IFNa
IFNg
IL10
IL12
IL13
IL15
IL17
IL1B
IL1RA
IL2
IL2R
IL4
IL5
IL6
IL7
IL8
IP10
MCP1
MIG
MIP1A
MIP1B
RANTES
TNFa
VEGF
